# The *C. elegans* Rab Family: Identification, Classification and Toolkit Construction

**DOI:** 10.1371/journal.pone.0049387

**Published:** 2012-11-21

**Authors:** Maria E. Gallegos, Sanjeev Balakrishnan, Priya Chandramouli, Shaily Arora, Aruna Azameera, Anitha Babushekar, Emilee Bargoma, Abdulmalik Bokhari, Siva Kumari Chava, Pranti Das, Meetali Desai, Darlene Decena, Sonia Dev Devadas Saramma, Bodhidipra Dey, Anna-Louise Doss, Nilang Gor, Lakshmi Gudiputi, Chunyuan Guo, Sonali Hande, Megan Jensen, Samantha Jones, Norman Jones, Danielle Jorgens, Padma Karamchedu, Kambiz Kamrani, Lakshmi Divya Kolora, Line Kristensen, Kelly Kwan, Henry Lau, Pranesh Maharaj, Navneet Mander, Kalyani Mangipudi, Himabindu Menakuru, Vaishali Mody, Sandeepa Mohanty, Sridevi Mukkamala, Sheena A. Mundra, Sudharani Nagaraju, Rajhalutshimi Narayanaswamy, Catherine Ndungu-Case, Mersedeh Noorbakhsh, Jigna Patel, Puja Patel, Swetha Vandana Pendem, Anusha Ponakala, Madhusikta Rath, Michael C. Robles, Deepti Rokkam, Caroline Roth, Preeti Sasidharan, Sapana Shah, Shweta Tandon, Jagdip Suprai, Tina Quynh Nhu Truong, Rubatharshini Uthayaruban, Ajitha Varma, Urvi Ved, Zeran Wang, Zhe Yu

**Affiliations:** Department of Biological Sciences, California State University East Bay, Hayward, California, United States of America; Dulbecco Telethon Institute at San Raffaele Scientific Institute, Italy

## Abstract

Rab monomeric GTPases regulate specific aspects of vesicle transport in eukaryotes including coat recruitment, uncoating, fission, motility, target selection and fusion. Moreover, individual Rab proteins function at specific sites within the cell, for example the ER, golgi and early endosome. Importantly, the localization and function of individual Rab subfamily members are often conserved underscoring the significant contributions that model organisms such as *Caenorhabditis elegans* can make towards a better understanding of human disease caused by Rab and vesicle trafficking malfunction. With this in mind, a bioinformatics approach was first taken to identify and classify the complete *C. elegans* Rab family placing individual Rabs into specific subfamilies based on molecular phylogenetics. For genes that were difficult to classify by sequence similarity alone, we did a comparative analysis of intron position among specific subfamilies from yeast to humans. This two-pronged approach allowed the classification of 30 out of 31 *C. elegans* Rab proteins identified here including *Rab31/Rab50*, a likely member of the last eukaryotic common ancestor (LECA). Second, a molecular toolset was created to facilitate research on biological processes that involve Rab proteins. Specifically, we used Gateway-compatible *C. elegans* ORFeome clones as starting material to create 44 full-length, sequence-verified, dominant-negative (DN) and constitutive active (CA) *rab* open reading frames (ORFs). Development of this toolset provided independent research projects for students enrolled in a research-based molecular techniques course at California State University, East Bay (CSUEB).

## Introduction

### The Rab Family of Monomeric GTPases

The RAS superfamily of monomeric GTPases is widely conserved and includes five main families: Ras, Rho, Arf, Ran and Rab. The largest of these, the Rab family, participates in all aspects of vesicular traffic and contributes to endomembrane identity [1], [2]. For example, Rab5 localizes to clathrin-coated vesicles budding from the plasma membrane, early endosomes and transport vesicles in between. These observations reflect Rab5’s specific role in clathrin uncoating and target selection of vesicles travelling from the plasma membrane to the early endosome. Similarly, Rab1 and Rab2 localize to distinct transport vesicles between the ER and golgi as they function during ER to golgi or golgi to ER transport, respectively [2]. Needless to say, all aspects of vesicular trafficking including intracellular transport of proteins, ligand secretion, receptor trafficking and protein degradation is fundamental to the function of the eukaryotic cell. Thus, it is not surprising that mutations in Rab GTPases, their regulators or effectors can lead to inherited and acquired disease including birth defects, mental retardation, type 2 diabetes, cancer and neurodegenerative disease [3]. Moreover, a number of bacterial pathogens express regulators of specific host Rabs to facilitate cell entry and avoid degradation by the lysosome, thereby promoting the process of infection [3].

Rab proteins, like all RAS superfamily members, alternate between active (GTP bound) and inactive (GDP bound) conformational forms [4]. The active form typically binds effector molecules while GTP hydrolysis disrupts this interaction. Interestingly, monomeric GTPases in isolation rarely cycle between active and inactive forms as these proteins are poor GTPases and once GTP hydrolysis occurs, GDP remains tightly bound. Thus, two types of regulatory proteins are essential to speed up cycling between active and inactive states. They include a GTPase activation protein (GAP), which promotes GTP hydrolysis and a Guanine Nucleotide Exchange Factor (GEF), which promotes GDP release. GTP is abundant in the cytoplasm and quickly replaces GDP. Not surprisingly, all members of the RAS superfamily can be identified through a number of conserved sequence motifs involved in guanine nucleotide binding and GTP hydrolysis, the so-called, G boxes (G1 through G5) [4]. These G box motifs, however, do not provide family specific identity. For this, one must identify sequence motifs that mediate interactions with family specific regulators, such as the Rab escort protein (REP), a protein involved in C-terminal prenylation of Rab family members only (see below).

### C-terminal Prenylation

Ras, Rho and Rab family members are typically prenylated at C-terminal cysteines, a modification that plays an essential role in membrane targeting and thus biological activity. Ras and Rho are prenylated directly by farnesyl transferase (FTase) or geranyl geranyl transferase I (GGTase I), enzymes that recognize a C-terminal cysteine in the context of a CA_1_A_2_X box (A_1_ is aliphatic, A_2_ is aliphatic but not aromatic and X is typically S, M, A, Q or L) [5]. By contrast, Rab proteins are substrates of the Rab geranyl geranyl transferase II (Rab GGTase II), which prenylates a motif that typically consists of two cysteines in a variety of contexts including XXXCC, XXCCX, XCCXX, CCXXX and sometimes CXXX (X = any amino acid) [5].

Unlike CAAX-box proteins, the rab prenylation motif is not recognized directly by Rab GGTase II. Instead, this role is “outsourced” to REP, which interacts with Rab proteins through its Rab binding platform and C-terminal binding region (CBR) [6]. The RAB:REP complex then binds to Rab GGTase II allowing prenylation of one or both C-terminal cysteines. The CBR motif interacts with a short hydrophobic patch within the hyper variable C-terminal region of Rab called the CIM (CBR Interaction Motif), which typically consists of an AXA motif (A = aliphatic, typically I, L, V, F and P. X = any amino acid) [6]–[8]. This interaction positions the C-terminal cysteines within close proximity to the geranyl geranyl transferase II active site. Interestingly, the C-terminal tail is not inserted into the GGTase II active site like CAAX-box tails but instead is placed along side. This is consistent with the ability of Rab GGTase II to prenylate C-terminal cysteines within a wide variety of contexts [7].

All Rab proteins share the RAB:REP interaction. Thus, Pereira-Leal et al. hypothesized in 2000 that this family might possess Rab-specific sequence elements that mediate this interaction [9]. Through a bioinformatics approach, they identified five so-called Rab Family (RabF) motifs (RabF1– RabF5). Composite models of two crystal structures (Rab7:REP and REP:GGTase II) are consistent with a role for RabF1–4 motifs in mediating interactions with two Rab family specific regulators, REP and RabGDI [6], [10]. RabGDI functions by regulating the reversible association of Rab proteins with cell membranes. By contrast, RabF5 maps to an internal region of Rab proteins but nonetheless possesses family specific variations and remains helpful in identifying Rab family members.

In this paper, we take a bioinformatics approach to identify the complete *C. elegans* Rab family based on percent identity to RabF motifs and absence of motifs specifically conserved in other Ras superfamily members. Next, we place individual Rab proteins into specific subfamilies based on a phylogenetic analysis with humans. For difficult to classify Rab proteins we also perform an analysis of intron position conservation. Finally, we create an ORFeome-based molecular toolset of mutant *rab* ORFs to be used for *rab* gene function studies in *C. elegans*. The *C. elegans* ORFeome resource was created in 2003 [11] and is a partially-verified, Gateway-compatible library of ORFs representing more than 63% of the proteome [12]. Our molecular toolset includes 44 full-length, sequence-verified, dominant-negative (DN) and constitutive-active (CA) mutant *rab* ORFs. Mutant forms were created in the context of a research-based lab course at California State University, East Bay (CSUEB) as a way to provide an authentic research experience to a large number of students.

Educating the next generation of research scientists has traditionally followed a master-apprenticeship model. While this model is highly effective in providing an authentic research experience, it impacts only a small number of students. To boost the number of students exposed to real research, there have been increasing calls for providing an authentic research experience during the academic year, within so-called research-based lab courses [13]–[15]. Inspired by a pioneer of research-based curricula [16], I (coauthor M.G.) initiated a research-based lab course in 2007. My aim was to provide a sense of adventure, discovery and pride in one’s accomplishments with the knowledge that students can contribute to the overall scientific knowledge base.

This research-based course focused on the Rab family for several reasons: 1) the *C. elegans* Rab family is manageable in size with 31 members [17] and this work. 2) The average *rab* ORF is short (median length: 632 bp). 3) DN and CA mutant forms first described in human Ras [18], [19] have also been used successfully in Rab proteins [20]–[24], [65], [67]. Finally, functional analysis of Rab family members is incomplete despite their vital roles in cell and developmental biology [1], [2].

## Results

### Identification of the Complete *C. elegans* Rab Family

Using a bioinformatics approach, Pereira-Leal and Seabra identified Rab family members in *C. elegans*, *D. melanogaster*, *H. sapiens*, *S. cerevisiae*, *S. pombe* and *A. thaliana* [17]. As this work was published just after completion of the *C. elegans* genome and improvements to ORF annotations have been ongoing [25], we redid a bioinformatics analysis of the *C. elegans* Rab family in this study.

**Figure 1:**
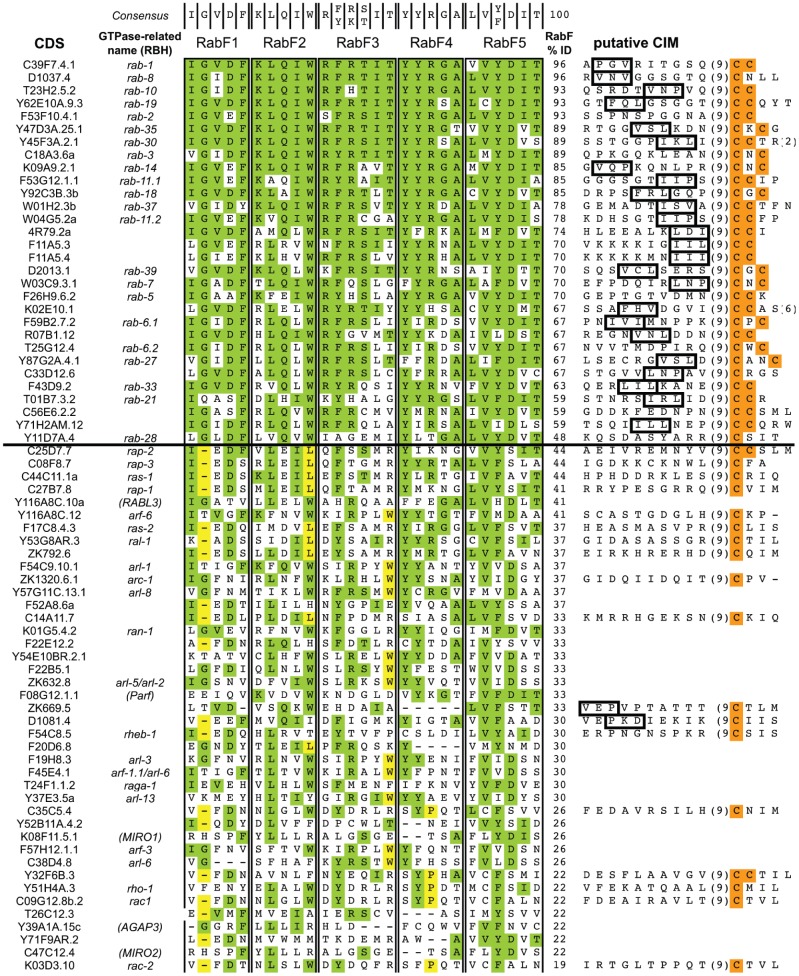
The complete list of PSI-BLAST hits using a sequence alignment of known *C. elegan* Rabs. Genes are listed in descending order of RabF % identity (ID). The consensus sequence used to calculate RabF % ID is listed in the top row. For each hit, the portion of the alignment corresponding to each Rab Family motif (1–5) is provided. By contrast, the C-terminal sequence is only provided for those hits with one or two near terminal cysteines. For each C-terminus shown, the putative CBR interacting motif (CIM) is boxed if present. C-terminal cysteines are shaded orange. RabF consensus matches are shaded green and specific amino acids that suggest inclusion in a nonRab family are shaded yellow. The bold horizontal line separates the cluster of mostly known rabs from other monomeric GTPases. Numbers in parentheses indicate the number of amino acids omitted from the C-termini. RBH =  Reciprocal Best Hit.

To identify new members of the Rab family in *C. elegans*, we first created a multiple sequence alignment (MSA) of the 28 Rab family proteins identified previously [17] using Muscle within MEGA5 [26]. We then used this MSA as a query in a Position-Specific Iterated BLAST (PSI-BLAST) [27]. This approach identified 51 additional “hits” deemed statistically significant (not counting splice variants). With the MSA of the expanded list, we then calculated a single RabF percent identity score based on conservation with the consensus sequences for RabF1 through RabF5 combined (27 amino acids in total). Putative Rab proteins sorted in descending order according to the RabF percent identity score are listed in Figure 1. Not surprisingly, the original 28 Rab proteins cluster at the top of the list with the most distant member (RAB-28) at position 30 with a RabF percent identity score of 48. Above RAB-28, at 59% and 67% are two new Rab proteins, R07B1.12 and Y71H2AM.12, not identified in 2001 although R07B1.12 (a.k.a. GLO-1) was recognized as a Rab protein more recently [28], [29].

All proteins listed above RAB-28 (Figure 1) are likely to be authentic Rabs. The majority of these proteins including R07B1.12 and Y71H2AM.12 possess a typical rab prenylation motif with two terminal (or near terminal) cysteines in addition to a recognizable hydrophobic CIM. Exceptions include RAB-8, RAB-28 and C33D12.6. These proteins contain a CXXX motif instead.

The presence of an atypical Rab prenylation motif in RAB-8 and 28 is well-documented within metazoans where the vast majority of known members possess a single cysteine in a CAAX-box like context [30]–[32]. C33D12.6 *(tag-312)* is a RAB45 ortholog (see below). While most RAB45 orthologs (42 species examined) possess two terminal cysteines in a CCXX context, RAB45 from *Branchiostoma floridae* and *Trichoplax adhaerens* possess a CXXX motif [33].

By contrast, only two proteins listed below RAB-28 have two consecutive cysteines near their C-termini, C25D7.7 and Y32F6B.3. C25D7.7 does not possess a recognizable CIM and its reciprocal best hit is human RAP2. Y32F6B.3 is most closely related to cdc42, a Rho GTPase, and possesses additional sequence elements consistent with its inclusion in the Rho family. In fact, the vast majority of genes listed below RAB-28 possess sequence elements that suggest inclusion in Ras, Arf or Rho families. For example, genes that have a single amino acid deletion in place of the conserved glycine in RABF1 are characteristic of Ras and Rho family members while the presence of a tryptophan within position 6 of RabF3 is characteristic of Arf family members [9]. Additional amino acids that are exclusively found in Ras, Arf, and Rho families but not Rab are highlighted in yellow (Figure 1).

There are only a handful of genes with RabF identity scores less than RAB-28 that also lack amino acids justifying classification as a nonRab. Of these, the vast majority code for a reciprocal best hit (RBH) of a nonRab human protein and lack C-terminal cysteines. One intriguing exception is ZK669.5. ZK669.5 has a 33% RabF identity score and is the only one below RAB-28 that has a C-terminal cysteine and a recognizable CIM. For these reasons, we include ZK669.5 in our phylogenetic analysis described below to gather support for or against placement of ZK669.5 within the Rab family. In summary, our analysis identified a total of 31 Rab proteins including the following new additions: Y71H2AM.12, *glo-1* and possibly ZK669.5.

### A Phylogenetic Comparison of *C. elegans* and Human Rabs

**Figure 2:**
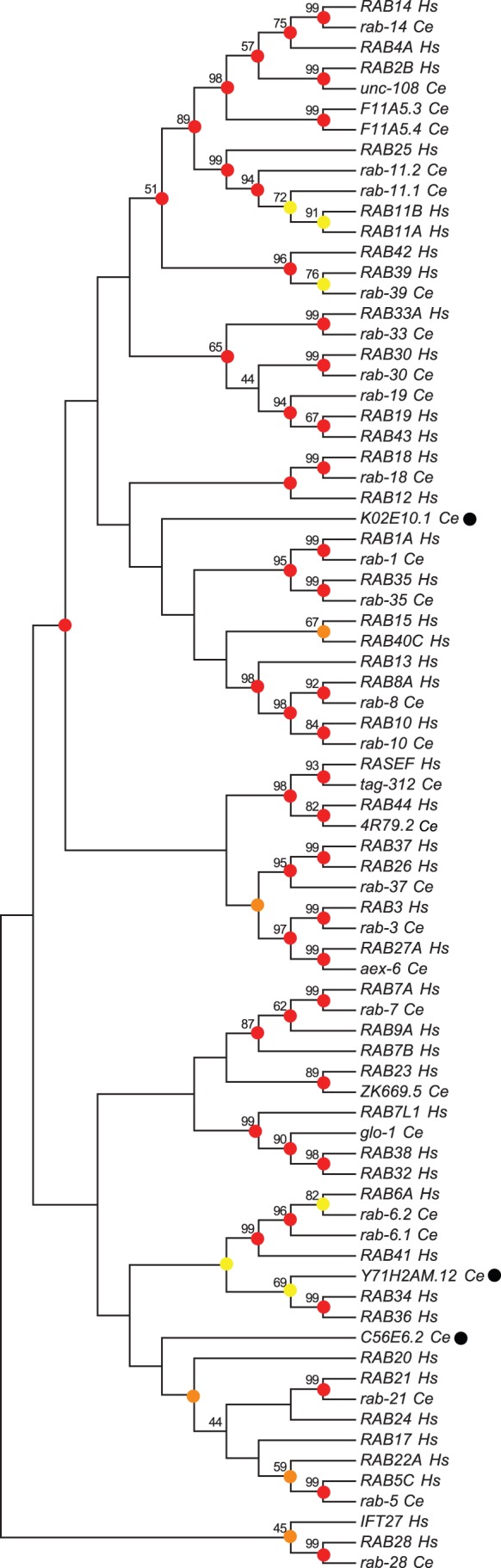
A chladogram of Rab family members from *C. elegans* and *H. sapien*s. The evolutionary history was inferred using the Neighbor-Joining phylogenetic reconstruction method. The tree is rooted with the natural outlying clade, Rab28. The optimal tree is shown with the percentage of replicate trees (>40) in which the associated genes cluster together in the bootstrap test (500 replicates) provided next to each branch. The tree is drawn to emphasize topology. The evolutionary distances were computed using the JTT amino acid substitution method and are in the units of the number of amino acid differences per site. Evolutionary analyses were conducted using MEGA5. Clades marked with red, orange or yellow circles indicate their degree of stability under a variety of phylogenetic reconstruction parameters (see text and methods for details). Red = 14/14, orange = 13/14 and yellow = 12/14 trees. Genes highlighted with black circles represent putative orphan *C. elegan* Rabs (lacking a human ortholog). For simplicity, closely related splice variants and well-supported human-specific clades were deleted (see methods for details).

Human orthologs had been identified for many but not all *C. elegans* Rab proteins [17]. Specifically, human orthologs had not been found for Y71H2AM.12, C33D12.6 (CeRabY1), 4R79.2(CeRabY2), K02E10.1 (CeRabY3), F11A5.4 (CeRabY4), F11A5.3 (CeRabY5), C56E6.2 (CeRabY6) and the putative Rab, ZK669.5. As the Rab family in mammals has grown since 2001, we redid a phylogenetic analysis here to determine if additional orthologous or paralogous clusters might be identified (see methods). In brief, we collected a nonredundant set of *C. elegans* and human Rab protein sequences from NCBI (see also, [34]). For example, if more than one splice variant existed, the one that included a prenylation motif was retained. Similarly, only one subfamily member of human-only clades with bootstrap support >98 in preliminary trees was included. A multiple sequence alignment was then used to create a neighbor-joining tree using the Jones Taylor Thornton (JTT) amino acid substitution method with 500 bootstrap replications (Figure 2). The same tree as an unrooted phylogram is also provided (Figure S2).

The terminal clades we observed are consistent with the previous tree [17] with the following additions/corrections. GLO-1 is grouped with a clade that includes RAB7L1 (RAB29), RAB32 and RAB38 (bootstrap support = 99). Consistent with this position, *glo-1* is the reciprocal best hit (RBH) and likely ortholog of human RAB32. *F11A5.4* (*CeRabY4*) and *F11A5.3* (*CeRabY5*) are paralogous with bootstrap support of 99 and are grouped within a clade that includes Rab14, 4 and 2 with bootstrap support of 98. C33D12.6/TAG-312 (CeRabY1) and 4R79.2 (CeRabY2) are members of the RAB45 (RASEF) and RAB44 subfamilies with bootstrap support of 93 and 82, respectively. Importantly, this phylogenetic analysis used an MSA that excluded the long N-terminal extensions rare in the Rab family but characteristic of these two subfamilies. In addition, human RASEF contains an EF Hand domain within its N-terminal extension, as does C33D12.6. There is also strong bootstrap support (98) for a clade that includes both RAB44 and RAB45 suggesting that these two subfamilies are paralogous and likely formed through a gene duplication event. We also observe moderate bootstrap support (89) for a clade that includes ZK669.5 and human RAB23.

**Figure 3:**
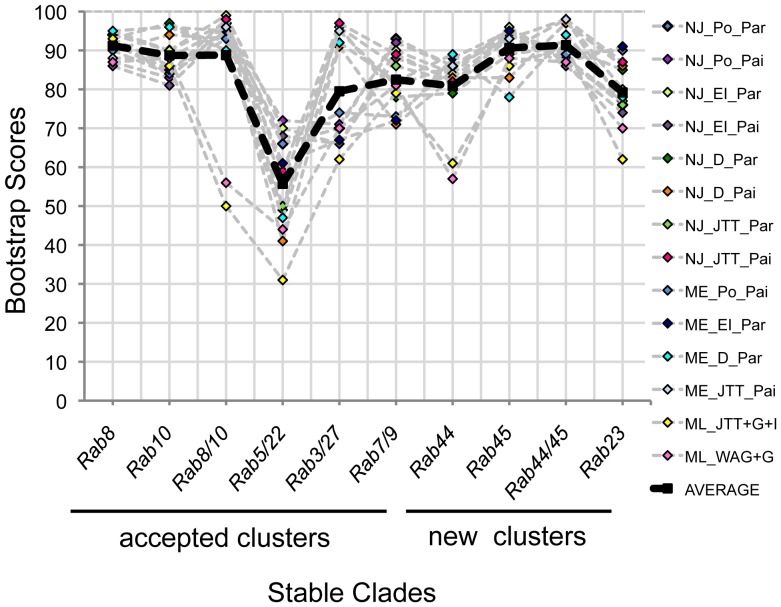
Bootstrap scores for specific terminal clades from 13 additional phylogenetic reconstructions. Thirteen additional phylogenetic analyses were performed using a combination of statistical methods. Phylogenetic reconstruction methods include Neighbor Joining (NJ), maximum likelihood (ML) or minimum evolution (ME). Amino acid substitution methods include Poisson (Po), JTT, Dayhoff (D), Equal Input (EI) and WAG. Gap deletion treatments include partial (Par) or pairwise (Pai) and rates and patterns of evolution include gamma distributed (+G), invariant sites (+I) or uniform (all others). All phylogenetic reconstructions were performed in MEGA5. Specific orthologous and/or paralogous clades include both worm and human Rab proteins. New orthologous clusters include bootstrap scores supporting the Rab44/4R79.2, Rab45/C33D12.6 and Rab23/ZK669.5 pairs. The new paralogous cluster includes the bootstrap scores that support the Rab44,4R79.2,Rab45 and C33D12.6 terminal clade.

The RAB23/ZK669.5 terminal clade is problematic for several reasons. ZK669.5 is only 29% identical to human RAB23 far below the 40% cutoff some use to classify Rab subfamily members [9], [35]. The branch lengths of this terminal clade are long and unequal (Figure S2). Finally, the *Rab23* subfamily (like *Rab28*) is a natural outlier in the Rab family phylogenetic tree [34], [36]. For these reasons, we worry that this cluster may result from long-branch attraction [37]–[40]. Long-branch attraction is sensitive to phylogenetic reconstruction method and choice of outgroup [37]–[39]. To rule out long-branch attraction in this instance, we performed 13 additional phylogenetic reconstructions (Figure 3). Each reconstruction used a unique combination of statistical method, amino acid substitution model, gap deletion treatment and rate and patterns of evolution.

Our results indicate that the original tree in Figure 2 is robust. Most terminal and/or near terminal clades including the Rab23/ZK669.5 cluster are stable in 14/14 trees (Figure 2, red circles). In addition, average bootstrap support for the RAB23/ZK669.5 clade is not significantly different from other clusters where an ancestral relationship is well accepted by other criteria (Figure 3). For example, though Rab3 and Rab27 are both involved in regulated secretion, bind to some of the same effectors and have overlapping function at the synapse in *C. elegans* [41]–[43], bootstrap support for this pair ranges from 63 to 98 with an average of 80. Similarly, though bootstrap support for the Rab5/22 pair ranges from 31 to 72 with an average of 56, an evolutionary relationship between Rab5 and Rab22 is well accepted: several effectors have been identified that bind both Rab proteins and a subset of exon/intron junctions are conserved between the two genes [17], [44]–[46]. Further, the well-accepted paralogs, Rab7 and Rab9 [47], have bootstrap support ranging from 71 to 93 with an average of 83. By comparison, bootstrap support for the ZK669.5/RAB23 clade ranges from 62 to 91 with an average of 79 (Figure 3). The ZK669.5/RAB23 clade is also stable when different outgroups are used including human KRas, Arf1 or a set of genes that include best nonRab hits for ZK669.5 (data not shown). The list of nonRab proteins used for this last analysis is provided (see Methods). Overall, these numbers are consistent with the assertion that bootstrap support above 95 may be too restrictive for this particular family of proteins [40].

As additional support for a RAB23/ZK669.5 clade, the top nonspecific domain hit for ZK669.5 in the Conserved Domain Database at NCBI is *rab23-like* (e value = 2.88e-11). In addition, TreeFam, a database of phylogenetic trees automatically created through the generation of seed trees that are progressively enlarged also classifies ZK699.5 as a Rab23 subfamily member (TreeFam ID: TF317494) [48]. Finally, the long isoform of ZK669.5 (ZK669.5a) contains a CAAX-like motif, an uncommon feature among Rabs in general but conserved within the Rab23 subfamily [31]. Together these results strongly suggest that ZK669.5 is a Rab and that human Rab23 and ZK669.5 share a common ancestor. From this perspective, the long unequal branch lengths observed (Figure S2), low percent identity and moderate bootstrap support suggests that ZK669.5 evolved more rapidly than its human counterpart.

### Classification of Y71H2AM.12 and C56E6.2 by Intron Position Conservation Analysis

*K02E10.1*, *Y71H2AM.12* and *C56E6.2* could not be classified by our molecular phylogenetic analysis (Figure 2). *Y71H2AM.12* clusters with human *RAB34* in 12/14 trees with low average bootstrap support (53) or with human *RAB6* (data not shown). *C56E6.2* and *K02E10.1* branches are long and not supported by bootstrap replicates (<30). These observations suggest two possibilities. *K02E10.1*, *Y71H2AM.12* and/or *C56E6.2* belong to a conserved subfamily lost in humans or rapid sequence divergence in *C. elegans* has obscured their ancestry. To distinguish between these two possibilities, we compared intron positions of *K02E10.1*, *Y71H2AM.12* and/or *C56E6.2* to Rab subfamily members most closely related to these orphan Rabs. Importantly, conservation of intron position has been observed among orthologs even when sequence identity is low and/or evolutionary distance is great [49], [50]. Moreover, multiple instances of conserved intron positions likely reflect common ancestry [51], [52].

**Figure 4:**
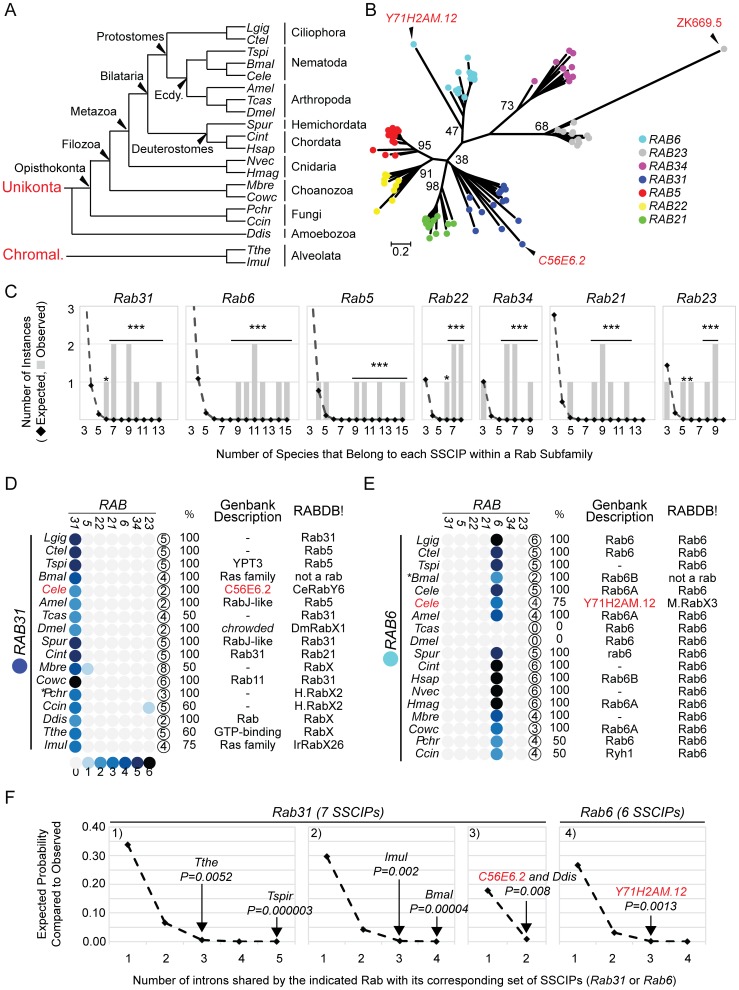
Comparative analysis of intron position among diverse Rab subfamily members. A) Cladogram indicating evolutionary relationships of 18 species examined here [128]–[130]. Ecdy. = Ecdysozoa, Chromal. = Chromalveolata. For species abbreviations see Methods. B) An ML tree of Opisthokonts created from the MSA used to map intron positions. Bootstrap support (100 replicates) is indicated for each subfamily cluster. C) For each subfamily, the number of times a Subfamily Specific Conserved Intron Position (SSCIP) involving the indicated number of species was observed (gray bars), compared to what is expected by chance (black diamonds). The difference between observed and expected is statistically significant where indicated. *P(Monte Carlo) <0.05. ***P(Monte Carlo)≤0.00001. The Rab*31, 6, 5, 22, 34, 21* and *23* subfamilies include 17, 18, 17, 9, 9, 10, 14 and 12 species, respectively. D) and E) Heat map indicating number of introns within *Rab31* (D) or *Rab6* (E) that match SSCIPs from *Rab31, 5, 22, 21, 6, 34* and *23*. The circled number indicates the number of introns present in the MSA for each gene. % equals the percentage of introns that are shared with the true SSCIP. *C56E6.2* (D) and *Y71H2AM.12* (E) are highlighted red. Genbank Descriptions (if any) and RABDB! classifications are included. Classification abbreviations include: HypoRabX1 (H.RabX1), HypoRabX2 (H.RabX2), HypoRabX3 (H.RabX3) and MetazoaRabX3 (M.RabX3). F) A pairwise comparison of intron position conservation between specific genes (Rab31 at left, Rab6 at right) and their corresponding set of SSCIPs. Black diamonds plot the probability that a specific number of intron matches would be expected by chance for each set of conditions. Chart 1 plots a comparison of 5 introns with 7 SSCIPs (5×7). Chart 2∶4×7. Chart 3∶2×7. Chart 4∶4×6. Observed values for a subset of genes are indicated with P values estimated from the Monte Carlo simulation data (See text and methods). Species abbreviations are as in A. C) and F) 72 protosplice sites assumed.

To identify potential orthologs of *K02E10.1*, *Y71H2AM.12* and *C56E6.2*, we used a “space hopping strategy” [53]. Each Rab orphan was first used as a query to identify top hits and/or reciprocal best hits (RBH) within two slowly evolving nematode species, *Trichinella spiralis* and *Brugia malayi* [54], [55]. Slowly evolving species are more likely to retain ancestral introns [56]. High quality hits were then used as queries in subsequent BLAST searches to identify top hits and/or RBHs within more distantly related species from the Opisthokonta. By analyzing disparate members of the Opisthokonta (Figure 4A), we hoped to distinguish ancestral introns from introns that are species or lineage-specific. Once all sequences were identified and tentatively classified, Rab subfamilies were individually aligned by Muscle with subfamily-specific gaps and nonconserved termini deleted. Next, all aligned and trimmed subfamilies were grouped and re-aligned to create one, large multiple sequence alignment (MSA) of 167 amino acids. Introns were mapped to this MSA and a Maximum Likelihood (ML) phylogenetic tree was created (see methods for details). Importantly, only introns that mapped within the MSA block were considered. Overall, this analysis involved 96 Rab sequences, 400 introns and 7 Rab subfamilies including *Rab5*, *Rab6*, *Rab21*, *Rab22*, *Rab23*, *Rab31* and *Rab34*. These subfamilies were included either because they were identified in the “space hopping strategy” described above or because they would serve as negative controls. Negative control sequences were necessary to determine whether intron position conservation is indeed subfamily specific.

The topology of the phylogenetic tree (Figure 4B) demonstrates the success of the “sequence hopping strategy”. Putative members of each subfamily formed monophyletic clusters. An enlarged view of each subfamily-specific clade is also provided (Figure S3).

Next, we identified subfamily-specific conserved intron positions (SSCIPs). An SSCIP is defined as an intron position that is conserved among subfamily members in three or more disparate species of the Opisthokonta suggesting a presence within an ancestor to metazoans (Figure 4A). An intron position is defined as conserved if it is located at an identical position and within the same phase of the codon. As defined, we found 5 SSCIPs in *Rab22*, 6SSCIPs in *Rab5*, *Rab6*, *34, 21* and *23* and 7 SSCIPs in *Rab31*. We then counted the number of introns that each individual Rab gene shared with each set of SSCIPs. Results for Rab31 and Rab6 subfamilies are displayed as an array of filled circles where darker shades correspond to higher numbers of shared introns and columns correspond to each subfamily included in this study (Figure 4D, E). The MSA including the relative intron positions of all subfamilies is also provided (Figure S4).

Overall, this analysis demonstrates that Rab subfamily members possess remarkable conservation of intron positions over long evolutionary distances. 87% of the 400 introns within the MSA map to SSCIPs. In other words, only 13% of the introns are specific to a single species or lineage. Moreover, none of the SSCIPs identified for *Rab5*, *6*, *21*, *22*, *23*, *31* and *34* map to the same location with the exception of two shared between *Rab5* and *Rab22*. This last observation is consistent with a previous report [17].

To quantify this level of conservation, a Monte Carlo simulation with 100,000 iterations was performed. In brief, all introns within each subfamily-specific MSA were randomly shuffled and the number of instances of conserved introns involving exactly 3, 4, 5 etc. species was counted. The data generated from this analysis was then used to estimate P values to assess statistical significance between what was observed and what could have occurred by chance (see Methods). Of the 41 SSCIPs identified only 5 could have occurred by chance with *P(Monte Carlo) >0.05. By contrast, 33 SSCIPs were extremely significant with ***P(Monte Carlo) <0.00001 (Figure 4C).

This analysis also allowed the unambiguous classification of *Y71H2AM.12* and *C56E6.2* to the *Rab6* and *Rab31* subfamilies, respectively. *Y71H2AM.12* possesses four introns within the MSA block of which three map to *Rab6* SSCIPs previously deemed statistically significant at the 0.00001 level. By contrast, none match intron positions of any other *Rab* gene analyzed including species and lineage-specific introns. The likelihood that at least three of the four introns in *Y71H2AM.12* might match *Rab6* SSCIPs by chance is extremely low with *P(Monte Carlo*) = 0.00001. This P value assumes that every nucleotide position within the MSA is a potential intron insertion site (501 sites total). If instead, one assumes that introns insert into genes at nonrandom positions called protosplice sites [51], the likelihood remains low with *P(Monte Carlo*) = 0.0013 (Figure 4F). In the latter analysis, the number of protosplice sites was set at 72 corresponding to one site per seven nucleotides [51]. This is likely an underestimate as the MSA block created here boasts 71 unique intron positions while only including a small fraction of the known *Rab* subfamilies (Figure S4). Interestingly, the uniquely-positioned intron in *Y71H2AM.12* is only three nucleotides away from another *rab6*-specific CIP and may represent a phenomenon known as intron sliding [57].

*C56E6.2* has two introns within the MSA block. Both map to *Rab31* SSCIPs previously deemed statistically significant at the 0.0001 level. By contrast, neither match intron positions of any other Rab gene analyzed. Again, the likelihood that both introns might map to *Rab31* SSCIPs by chance is low, with P values equaling 0.00016 or 0.008 depending on the number of protosplice sites assumed: 501 or 72 (Figure 4F). Moreover, C56E6.2 is the reciprocal best hit of XP_003374270.1 and XP_001901062.1, *Rab31* orthologs from *T. spiralis* and *B. malayi*, respectively. As predicted for slowly evolving species, intron position conservation of Rab31 from these nematode species is higher than in *C. elegans*. Specifically, 5/5 introns within *Rab31* from *T. spiralis* and 4/4 introns within *Rab31* from *B. malayi* match *Rab31* SSCIPs. Needless to say, these observations are statistically significant (Figure 4F).

By contrast, introns in K02E10.1 do not match any of the SSCIPs present in subfamilies that clustered nearby (Figure 2 and data not shown) including *rab1, 8, 10, 12, 15, 18, 35* and *40* (data not shown). It is worth noting that this Rab gene is atypical. It contains a methionine instead of glutamine at position 70 (Q70M) suggesting that it may not function as a GTPase [58]. Moreover, an attempt to isolate an ORFeome cDNA corresponding to this ORF failed [12] suggesting the possibility that this locus codes for a pseudogene or has been mis-annotated. Thus, the evolutionary history of K02E10.1 remains mysterious.

Also, all 3 introns present within the conserved portion of ZK669.5 are uniquely positioned (Figure S4). Importantly, an absence of intron conservation with *Rab23* SSCIPs does not indicate that its classification by molecular phylogenetics is incorrect. There are numerous instances where clear orthologs do not possess introns that map to the expected SSCIPs. This is particularly true of rapidly evolving species within the Ecdysozoa including *C. elegans* and *D. melanogaster* [56]. Examples within this dataset include *rab-5* from *C. elegans*. Despite overwhelming bootstrap support for its classification as *Rab5* (Figure 2), none of its four introns are conserved with the 5 *Rab5* SSCIPs identified here (Figure S4). Similarly, the single intron within *Rab23* of *D. melanogaster* fails to match any of the 6 *Rab23* SSCIPs. Ultimately, the classification of ZK669.5 as a *RAB23* subfamily member will require additional evidence that does not solely rely on sequence or intron position conservation (i.e. functional data). To date, its function is poorly understood. There are no mutant alleles that map to ZK669.5 and high-throughput RNAi screens have not identified obvious abnormalities [59], [60].

### *C56E6.2/Rab31* Belongs to an Ancient Rab Subfamily

*Rab31* is a poorly characterized subfamily previously identified in a small number of Opisthokonts [33]. Confusing matters, many Rab proteins have been erroneously annotated as *Rab31* in Genbank and Ensembl (see Discussion). To learn more about the evolutionary origins of *Rab31* we searched for orthologs in a small number of more distantly related species including *Dictyostelium discoideum* (an Amoebozoa), *Tetrahymena thermophila* and *Ichthyophthirius multifiliis* (two ciliates from the supergroup Chromalveolata). Specifically, we used *Rab31* subfamily members from the Opisthokonta (14 total) as queries in BLASTP. XP_642644.1 from *D. discodeum* was the top hit by BLASTP for 9 of the 14 Rab31 members tested. Moreover, it was the RBH for *Rab31* subfamily members from *S. purpuratus*, *A. mellifera, M. brevicollis* and *C. owczarzaki.* Consistent with its classification as a *Rab31* subfamily member, both of its introns match *Rab31* SSCIPs (Figure 4D), an observation that is statistically significant (Figure 4F).

Using a similar strategy, paralogs XP_001020942.1 and XP_001025858.2 were identified as putative *Rab31* subfamily members from *T. thermophila*. Importantly, they possess an identical exon-intron structure (Figure S4). In addition, 3/5 introns map to *Rab31* SSCIPs. Finally, EGR30930.1 from *I. multifiliis* was identified as a RBH of XP_001020942.1 from *T. thermophila.* Its corresponding Rab gene has 4 introns. All are conserved with *Rab31* members from *T. thermophila* and three match *Rab31* SSCIPs (Figure 4D). Again, these observations are statistically significant by Monte Carlo simulation (Figure 4F).

Eukaryotes can be subdivided into 5 or 6 so-called supergroups [61], [62]. The tree of life includes Chromalveolates, Unikonts, Rhizaria, Excavata and Plantae [62] if 5 supergroups are counted. To make 6 supergroups, Unikonts are split into Amoebazoa and Opisthokonts [61]. Our intron position data identified Rab31 in Opisthokonts, Amoebazoa and Chromalveolata. These results suggest that *Rab31* is an ancient Rab subfamily that arose before the split of the Opisthokonta, Amoebazoa and Chromalveolata. While the topology of the tree remains murky at its base [62], the presence of Rab31 in 2 or 3 supergroups suggests that it may have been present in the LECA.

### Independent Verification of Rabifier, an Automated Rab Classification Pipeline

In 2011, Diekmann et al. published Rabifier, an automated bioinformatics pipeline for the identification and classification of Rab proteins [33]. This tool was validated against three manually curated *Rab* families from *Trypanosoma brucei*, *Entamoeba histolytica* and *Monosiga brevicollis*. They documented 99% accuracy for Rab family identification and 71% to 90% (high confidence) accuracy for subfamily classification.

A comparison of the *C. elegans* Rab family identified and classified by “Rabifier” with the manually curated family described here is consistent with their published rate of accuracy. Only three differences were observed. Specifically, strong phylogenetic and/or intron position data presented here suggest that ZK669.5, Y71H2AM.12 and C56E6.2 should be classified as *rab23*, *rab6* and *rab31*, respectively. Instead, Rabifier classifies these proteins to undefined subfamilies HypoRabX1, MetazoaRabX3 and CeRabY6 (www.rabdb.org). Interestingly, this high rate of success for Rab protein classification may not extend to all subfamilies. Rabifier correctly classifies only four of the 17 *Rab31* genes manually identified here, a success rate of 24%.

### Identification of Error-free ORFeome-based WT Rab Clones

In 2007, students of a Molecular Techniques course at California State University, East Bay initiated the production of an ORFeome-based toolkit that attempted to include all 29 members of the Rab family known at the time (not including Y71H2AM.12 or ZK669.5). Such a toolkit will not only be useful for studying Rab function *in vitro* and *in vivo* but can also be used to manipulate or label specific regions within the endomembrane system for a wide variety of purposes.

The first step in creating an ORFeome-based toolkit was to identify error-free WT isolates from the ORFeome library. Importantly, ORFeome clones were originally created by PCR amplification of individual ORFs with Gateway-tailed ORF-specific primers. Each amplicon was then recombined into a pDONR vector, transformed into *E. coli* then 50–1000 colonies were frozen *en masse* for distribution. Thus, glycerol stocks for each ORF-containing entry clone are polyclonal [11]. Polyclonal pools were intended to capture alternate splice forms thereby increasing the utility of the library, however, they also captured clones with primer synthesis and PCR-based errors. Ultimately, errors were observed at a rate of 1 in 1232 bp [11].

**Figure 5:**
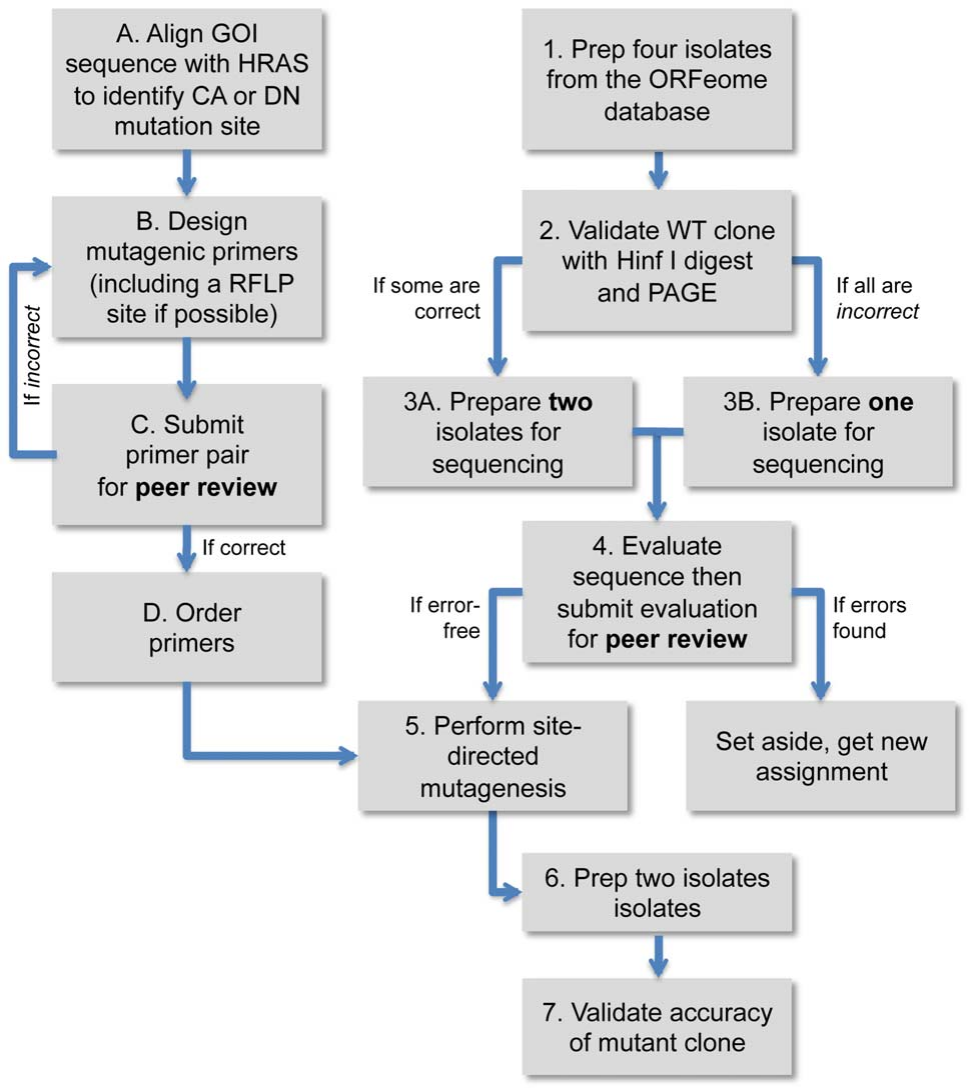
A flow chart describing the lab module involving verification and modification of ORFeome Rab clones. Steps 1 through 4 were done in parallel to steps A through D. Two peer-review steps at 3 and D were included to minimize mistakes in primer design and sequence analysis of WT ORFeome clones. Abbreviations: Gene of Interest (GOI), Constitutive Active (CA), Dominant Negative (DN), Restriction Fragment Length Polymorphism (RLFP), Polyacrylamide Gel Electrophoresis (PAGE), Human Ras (HRAS), Wild-Type (WT).

To identify error-free WT ORFeome clones, a pair of students followed steps one through four outlined in Figure 5. Specifically, each pair purified four isolates of a given *rab* ORFeome clone. Two of the four isolates with the expected HinfI restriction fragment pattern by Polyacrylamide Gel Electrophoresis (PAGE) were then sequenced. If at least one clone was error-free, this isolate was used as a template to create the two mutant clones (DN and CA). If neither “WT” clones were error-free, the gene was set aside for a future class to try again with an additional four isolates. If the restriction fragment pattern for all four isolates was abnormal, one isolate was sequenced to rule out (or in) the possibility that the exon/intron structure had been mispredicted. A maximum of 8 isolates were analyzed. If all 8 isolates contained errors, the clone was not studied further.

Ultimately, this strategy identified 22 WT Rab ORFs to be used as template for site-directed mutagenesis. Specifically, 19 Rab ORFs had 100% identity to Refseq protein sequences (excluding start and stop codons). Two Rab ORFs had minor differences to their corresponding Refseq and one Rab ORF had an exon/intron structure distinct from what had been predicted (Table 1).

**Table 1:** A list and description of Rab isolates created for the *C. elegans* ORFeome-based toolkit.

Other Name(s)	Sequence Name	Rab Subfamily	Refseq with Best Match(% Identity)	WT	DN	CA
*rab-1*	C39F7.4	Rab1	NP_503397.1 **(100)**	IK3-1	IK20-1	PP50-1
*unc-108/rab-2*	F53F10.4	Rab2	NP_491233.1 **(100)**	GC5-1	GC33-2	SM15-1
*rab-3*	C18A3.6	Rab3	NP_001021974.1 **(100)**	AP2-1	NG33-1	CG7-1
*rab-5*	F26H9.6	Rab5	NP_492481.1 **(100)**	PD3-1	NH11-1	MJ21-1
*rab-6.1*	F59B2.7	Rab6	NP_498993.1 **(100)**	SDS6-1	PRS33-2	SDS34-1
*rab-6.2*	T25G12.4	Rab6	NP_510790.1 **(100)**	AV3-2	NJ16-1	AV11-1
*rab-7*	W03C9.3	Rab7	NP_496549.1 **(100)**	MG2-1	SI46-4	SI28-5
*rab-8*	D1037.4	Rab8	NP_491199.2 **(100)**	MB5-2	DK26-1	MB38-1
*rab-10*	T23H2.5	Rab10	NP_491857.1 **(100)**	MB10-1	SBA42-1	CN28-1
*rab-11.1*	F53G12.1	Rab11	NP_490675.1 **(100)**	ZY2-2	PRI8-7	ZY10-1
*rab-11.2*	W04G5.2	Rab11	N/A	OST indicates retention of intron one		
*rab-14*	K09A9.2	Rab14	NP_510572.1 **(100)**	SP6-1	MHB43-1	SP51-1
*rab-18*	Y92C3B.3	Rab18	N/A	ORFeome clone is a *rab-18*::*mlc-3* fusion		
*rab-19*	Y62E10A.9	Rab43	NP_502576.1 **(100)**	NM7-2	KM29-1	DJM14-1
*rab-21*	T01B7.3	Rab21	NP_495854.1 **(100)**	LAK5-1	JS31-4	DS25-1
*aex-6/rab-27*	Y87G2A.4	Rab27	NP_493376.1 **(100)**	JP6-1	JP38-1	SAN46-1
*rab-28*	Y11D7A.4	Rab28	NP_501609.1 **(100)**	SVP6-1	ST21-1	SVP57-2
*rab-30*	Y45F3A.2	Rab30	NP_499328.1 **(100)**	MEL4-1	TT18-1	SMJ11-1
*rab-33*	F43D9.2	Rab33	NP_499314.1 **(100)**	TR6-1	TR34-1	MAH25-1
*rab-35*	Y47D3A.25	Rab35	N/A	ORFeome clone contains a *rab-27* insert.		
*rab-37*	W01H2.3	Rab37	NP_001041293 **(100)**	RV3-0	ZW10-1	KLD50-1
*rab-39*	D2013.1	Rab39	N/A	ORFeome clone lacks the C-terminus.		
*CeRabY1/tag-312*	C33D12.6	Rab45	NP_508523.1 **(96)**	DVD8-2	DVD36-2	AMT92-13
*CaRabY2*	4R79.2	Rab44	NP_503120.1 **(100)**	LGK3-2	LGK29-1	AMT92-5
*CeRabY3*	K02E10.1	*orphan*	N/A	OST does not confirm ORF		
*CeRabY4*	F11A5.4	Rab2-like	NP_507084.1 **(99)**	KK9-2	AMT92-1	AMT92-9
*CeRabY5*	F11A5.3	Rab2-like	NP_507083.1 **(100)**	EB8-2	MR49-1	PM16-1
*CeRabY6*	C56E6.2	Rab31/Rab50	N/A	ORFeome clone did not grow.		
*glo-1*	R07B1.12	Rab32	N/A	ORFeome clone not in the database.		
*N/A*	Y71H2AM.12	Rab6-like	N/A	Not examined		
*N/A*	ZK669.5	Rab23-like	N/A	Not examined		

WT, DN and CA clones included in the Rab Toolkit are given isolate names otherwise an explanation for its absence is provided. Subfamily classifications are based on Diekmann et al. 2011 [33] and/or data presented here. The majority of *C. elegans rab* genes are predicted to have only one splice variant with the following exceptions: WormBase describes two splice variants for *rab-3* that code for proteins 233 and 219 amino acids (aa) in length. ORFeome project primers were designed to amplify the shorter isoform only. WormBase describes two splice variants for *4R79.2 (Rab44)* that code for proteins 311 aa and 395 aa in length. ORFeome project primers were designed to amplify the longer isoform only. Names listed under “other” are from WormBase or Pereira-Leal and Seabra (2001). Finally, while *rab-37* shows 100% identity with the Refseq protein NP_001041293 it contains an additional 5 amino acids at its N-terminus. See text for details.

The remaining seven Rab ORFs were not included in the toolkit for a variety of reasons. *glo-1* (R07B1.12) was not present in the ORFeome database. C56E6.2 failed to produce viable colonies. K02E10.1 and W04G5.2 ORFs were not confirmed by their ORF sequence tag (OST). Finally, ORFeome clones for *rab-18 (Y92C3B.3)*, *rab-35 (Y47D3A.25)* and *rab-39 (D1013.1)* were found by the students of the research-based course to be unusable. Specifically, the *rab-18* ORFeome clone contained a *rab-18::mlc-3* gene fusion. The *rab-35* ORFeome clone contains *rab-27* and the *rab-39* ORFeome clone lacks the true C-terminus. Importantly, the C-terminus of *rab-39* described in NP_495984 is supported by cDNA evidence (data not shown) while the C-terminus of the *rab-39* ORFeome clone is not. All ORFeome clones that are included in the toolkit but deviate from their reference sequence are described in more detail below.

All four ORFeome isolates of *F11A5.4* and *rab-37* differed from their corresponding Refseq protein accession sequences but were retained in the toolkit to be used with caution. The F11A5.4 ORF has a cysteine to tyrosine missense mutation at position 180 (C180Y), the result of a G539A transition. This missense mutation is also found in its OST, suggesting that it results from an early PCR error or error in the sequenced *C. elegans* genome. Importantly, the only experimental evidence confirming this ORF is the OST from ORFeome. In addition, the cysteine at position 180 falls outside of the Rab domain and is not conserved with its closely related paralog, F11A5.3. The significance of this missense mutation is not known. For *rab-37*, ORFeome project primers were designed to amplify a protein described in AAB52888 now deemed obsolete. This isoform is identical to the short isoform of *rab-37* (NP_001041293) except that it contains five additional amino acids (MFLKV) at its N-terminus. This addition is not expected to impact *rab-37* function as N-terminal Rab fusions are well-tolerated [63]–[65].

**Figure 6:**
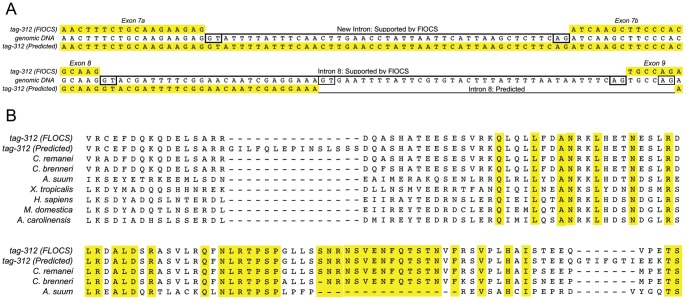
Full-length ORF sequence of *tag-312* (Rab45) isolates reveals a different splice pattern than predicted. A) A nucleotide alignment of *tag-312* (FlOCS), genomic DNA and the predicted *tag-312* ORF at two regions where exon/intron splice junction differences were found. In the top alignment, FlOCS reveals a new intron, splitting predicted exon 7 into exons7a and 7b. In the bottom alignment, FlOCS reveals an alternate 5′ splice donor and 3′ splice acceptor for intron 8. Compare FlOCS-supported 5′ and 3′ splice sites boxed in bold to predicted 5′ and 3′ splices sites (boxed, not bold). B) Two multiple sequence alignments of Rab45 subfamily members spanning the two regions described in 5A above demonstrate the impact of FLOCS-supported gene structure differences. The alignment includes proteins from the nematodes, *Caenhorabditis elegans*, *tag-312*(FlOCS) and NP508523.1, *Caenhorabditis brenneri, Caenhorabditis remanei* and *Ascaris suum* in addition to *Xenopus laevis* (frog), *Homo sapiens* (human), *Monodelphis domestica* (opossum) and *Anolis carolinensis* (lizard). The intron that splits exon 7 into two creates a 15 amino acid deletion that is conserved among all species examined (top). The alternate intron 8 creates an Indel in a region of Rab45 that is conserved among nematodes only.

The Full-length ORFeome Clone Sequence (FlOCS) of *CeRabY1(C33D12.6)*, the putative ortholog of human RAB45 (RASEF) indicates the existence of a different exon/intron structure from what had been predicted (Figure 6A). Specifically, exon 7 (predicted) is interrupted by a 45 bp intron creating a 15 amino acid in-frame deletion conserved across diverse phyla including humans (Figure 6B). In addition, intron 8 is shifted 5′ and enlarged creating an in-frame InDel involving 10 amino acids (Figure 6A). While the Indel falls in a region conserved only among other nematodes (Figure 6B), the 5′ and 3′ splice sites (SS) of intron eight (FlOCS) match the *C. elegans* SS consensus sequence equal to or better than the predicted intron 8 [66]. Importantly, all four isolates were identical by HinfI digestion and PAGE analysis suggesting that the gene structure was likely a misprediction, not an example of alternative splicing (data not shown). The only other Rab gene not previously supported by complete EST or OST evidence was *rab-33*. In this instance, FlOCS data confirms the accuracy of the internal exon/intron boundaries of *rab-33*. For FlOCS data of all 22 WT ORFeome clones described here see Figure S5 (Accession numbers are also provided in the Materials and Methods).

### Generation of Dominant Negative and Constitutive Active Clones

Each student was tasked to create an ORF coding for the Q70L constitutive active (CA) or T17N dominant negative (DN) mutant form, initially characterized in HRas [18], [19]. Importantly, these missense mutations have also been shown to transform Rab GTPase family members into DN or CA mutant forms in a variety of organisms including yeast, humans, *Drosophila*, and *C. elegans* [20]–[24].

To generate mutant clones, students first followed steps A through D outlined in Figure 5. In brief, each student used Clustal W to create a multiple sequence alignment that included the conceptual translation of their assigned *rab* ORF to identify the worm amino acid that corresponds to either Q70 or T17 in HRas. Once found, each student designed mutagenic primers then performed a Quikchange site-directed mutagenesis reaction (Agilent Technologies) using a sequence verified WT *rab* clone as template. A list of mutagenic primer sequences used is provided in Table S1. Since the vast majority of mutagenic primers were designed to create or destroy a restriction enzyme site, successful clones were first identified by restriction enzyme digestion then single isolates with the correct pattern of fragments were sequenced. In this way, DN and CA mutants were created for all 22 WT (or near WT) *rab* ORFs. During the course of this work, we witnessed phenomenal accuracy with Quikchange site-directed mutagenesis, which created no errors out of 52,408 nt sequenced (data not shown). Furthermore, the attL sites flanking each ORF are functional as each entry clone was successfully used in a recombinational cloning reaction with LR clonase (data not shown). Importantly, isolate MB38-1 (rab-8 CA) functions as expected when expressed within ciliated neurons to disrupt vesicle transport to the cilia [67].

## Discussion

Consistent with recent results [33], we expand the size of the *C. elegans* Rab family by three to 31 members. New members include, Glo-1, Y71H2AM.12 and ZK669.5. We also provide strong to moderate bootstrap support for the orthologous pairing of C33D12.6 (CeRabY1) with human RAB45, 4R79.2 (CeRabY2) with human RAB44 and ZK669.5 with human RAB23. Finally, a comparative analysis of intron position also allowed the classification of Y71H2AM.12 and C56E6.2 as Rab6 and Rab31, respectively. Only K02E10.2, a possible pseudogene, remains an orphan.

29 of the 30 classified Rabs are assigned to subfamilies also found in humans. One exception is *Rab31*, a subfamily that is poorly understood, often misclassified and mostly overlooked. Data presented here indicates that *Rab31* is present within the Opisthokonta, Amoebozoa and supergroup Chromalveolata. By some standards [33], [68], this suggests that *Rab31* was present in the last eukaryotic common ancestor or LECA. This conclusion is consistent with work done in parallel by Elias et al. 2012. One important difference, Rab31 is named Rab50 [68]. We support this name change as many Rab proteins classified as Rab31 in Genbank and Ensembl are in fact members of the Rab22 subfamily (this work and [33]). For example, the human Rab protein (NP_006859.2) designated as *RAB31* by the HUGO Gene Nomenclature Committee (HGNC) clusters with *Rab22* (Figure S3) and possesses introns that match *Rab22* SSCIPs (Figure S4). Until a name change is accepted we will refer to this subfamily as *Rab31/Rab50*.

*Drosophila’s chrowded* (*chrw*) is the only *Rab31/Rab50* subfamily member whose function has been described [69]. *chrw* was identified in a forward genetic screen for genes required for peripheral nervous system (PNS) development. Specifically, the PNS of mutant animals harboring a revertible transposon positioned directly 5′ to the *chrw* coding sequence (CDS) are disorganized with thick axons. More recently, Elias et al. 2012 used a novel high resolution phylogenetic approach called ScrollSaw to place the *Rab31/Rab50* subfamily within a well-supported, higher-order clade that includes *Rab21, 24, 20, 5* and *22* suggesting that Rab31/Rab50 may have been part of the core endocytic pathway within the LECA [68].

While Rab31/Rab50 is the only ancient subfamily in *C. elegans* that is absent in humans, many are present in humans but absent in *C. elegans* including Rabs 4, 9, 20, 22, 24, 29, 34, 40 and IFT27. Most of these Rab proteins are also absent in *Drosophila melanogaster*, another member of the Ecdysozoa [54]. These observations are consistent with whole genome studies that indicate that *D. melanogaster* and *C. elegans* are highly derived with widespread gene loss [70], [71]. A few, however, are present in *D. melanogaster* but lost or divergent in *C. elegans* including Rabs *4, 9, 23* and *40* (this paper and [33]). Of these, Rabs 4, 40 and 23 function in part by regulating the transport of specific developmental control proteins [65], [72]–[77]. It is worth noting that the Ecdysozoa is the largest and most diverse of the superphylum within animalia with over 4.5 million species [78]. Perhaps then, it is not surprising that dramatic changes and/or gene loss has been observed among these Rab proteins. Indeed, loss of specific developmental control genes and pathways within *C. elegans* but not *Drosophila* has been well-documented [79]–[81].

### Conservation of Intron Position and the Classification of Rab Proteins

In this work, we used conservation of intron position to classify Rab subfamilies that were otherwise difficult to classify using molecular phylogenetics. Our success with the classification of Y71H2AM.12 as Rab6 and C56E6.2 as Rab31/Rab50 validates this approach and highlights inherent limitations with the molecular phyologenetic approach, which relies on bootstrap support above a specific value. For example, the Rab31 subfamily cluster from the Opisthokonta examined here is supported by a bootstrap score of only 38 and the Rab6 subfamily cluster including Y71H2AM.12 is supported by a bootstrap score of only 47 (Figure 4B). By all criteria this level of support is unacceptable. It’s not surprising then that previous work employing phylogenetic analysis alone each missed specific *C. elegans* Rab proteins. Diekmann et al. failed to classify C56E6.2 (Rab31/50), ZK669.5(Rab23) and Y71H2AM.12(Rab6) [33]. Elias et al. 2012 failed to classify Y71H2AM.12 and misclassified two Rab31/Rab50 proteins from *Tetrahymena thermonila* as Rab22 [68]. Finally, TreeFam failed to classify C56E6.2 (Rab31/Rab50) (TreeFam ID = TF352282) [48]. While molecular phylogenetics is quick, powerful and mostly accurate, we argue that an intron position analysis may facilitate the classification of poorly supported clades. Moreover, the utility of intron position data is not limited to the identification of subfamily members. It can also be used to answer important evolutionary questions including but not limited to mechanisms underlying intron evolution and/or formation of the LECA.

One defining feature of the eukaryote is its large, endomembrane system comprised of multiple compartments and complex mechanisms of vesicle transport. In one model [82], the endomembrane system evolved from a single internal compartment regulated by a small set of proteins including one or a few Rab proteins. In an iterative process that occurred pre-LECA, compartment expansion occurred alongside gene duplication, sequence diversification and specialization of protein function. In support of this hypothesis molecular phylogenetic analyses indicate that the LECA is comprised of up to 23 Rab subfamily members [33], [68]. Because this expansion occurred pre-LECA, a comprehensive analysis of intron position conservation among Rab family members present within the LECA can complement existing efforts to garner insight into the evolution of the endomembrane system pre-LECA [68], [83]. For example, in one model, intron invasion is concomitant to eukaryogenesis [82]. In our small dataset of 7 Rab subfamilies, none of the SSCIPs overlap except for two between Rab22 and Rab5. This is particularly interesting in light of recent results by Elias et al. 2012 that provide phylogenetic evidence to support a super-clade within the LECA that includes a subset of the Rab subfamilies analyzed here (Rab21, Rab50/31, Rab5 and Rab22). One intriguing possibility is that Rab family expansion and thus expansion of endomembrane complexity occurred prior to the invasion of introns. Again, this hypothesis and others will require a more comprehensive analysis of intron position conservation among Rab proteins. Until then, alternative explanations cannot be excluded. For example, initial intron invasion may have been far more extensive than previously thought followed by variable rates of intron loss within specific clades. Nonetheless, such an analysis will benefit from the careful selection of species with low rates of intron gains and/or losses [56], [84], [85].

### Verified WT and Mutant *C. elegans* ORFeome Clones Facilitate Rab Function Studies

The *C. elegans* ORFeome Project, the semi-automated cloning of a near-complete set of full-length *C. elegans* ORFs has filled important gaps in our knowledge of gene structure, genome organization, variation and evolution. Combined with recombinational cloning strategies, this Gateway-compatible ORF collection has also been used in semi-automated, large-scale gene function studies with great success [59], [86], [87]. With the astounding amount of data collected for each of these large-scale studies, there has also been an inevitable loss of data that occurs when nonfunctional clones are unknowingly included in an experiment. This loss is not only tolerated but also expected. By contrast, those that use one or a small number of ORFeome clones in low-throughput study [88]–[90] cannot tolerate any level or error. With the toolkit developed here, not only have the students from Advanced Molecular Techniques at California State University, East Bay (CSUEB) created a set of useful mutant clones (CA and DN) of 22 *rab* genes but they have also generated a set of fully-verified WT isolates.

The ORFeome-based toolkit described here is Gateway-compatible. Thus each WT, DN and/or CA *rab* ORF is ready for recombinational cloning into a wide-variety of available destination vectors for biochemical and/or genetic analysis including the expression of Rab fusions in *C. elegans* [91]–[93], *E. coli* [94], insect [95] and/or yeast cells [86]. For example, with the expression of Rab fusions in *E. coli*, insect or yeast cells one can identify proteins that interact with *C. elegans* Rabs with pull down assays and/or yeast two-hybrid screens. We expect that the CA form will be particularly useful to identify Rab protein effectors as this form is stuck in the active conformation [96]. Expression of Rab fusions (i.e. to GFP) in *C. elegans* will also be useful for studying the morphology and dynamics of specific subcellular structures [97], [98], in addition to probing both loss- and/or gain-of-function phenotypes in a cell or tissue of interest [67].

For *in vivo* analysis of *rab* gene function, we recommend that the stop codon absent in *C. elegans* ORFeome clones be restored by site-directed mutagenesis prior to use in recombinational cloning. For maximum versatility, the ORFeome clones were intentionally designed to lack the A of the ATG and the last two nucleotides of the stop codon [11]. Thus, expression clones created through Gateway recombination of destination vectors with ORFeome entry clones express fusion proteins that include (at a minimum) a peptide sequence of nine amino acids at both the N and C-termini due to the absence of the stop and start codons and the presence of attB sites (25 bp in length). It is not clear how this extra peptide sequence will impact Rab prenylation as the C-termini of Rab proteins are positioned alongside the active site in a bent conformation [8]. In fact, Wu et al. do not observe a reduction in prenylation when up to 5 arbitrarily chosen amino acids are added to the C-terminus but they do notice that hydrophobic patches (i.e. a CIM mimic) within a C-terminal extension can be inhibitory. A conceptual translation of attB shows the presence of a putative CIM (underlined): Y P A F L Y K V V. Furthermore, Rab proteins that possess a single cysteine in a CAAX-box-like context may be prenylated by FTase and/or GGTase *in vivo* [31], [32], [99], [100]. These enzymes require the insertion of the CAAX-box tail into a binding pocket suggesting that CAAX-box proteins would not tolerate the nine amino acid extension.

To recreate the erstwhile stop codon, we recommend inserting the necessary nucleotides by site-directed mutagenesis (SDM) so to leave the 3′ attL site untouched. Our experience reassures us that Quikchange SDM is not likely to incorporate unwanted point mutations, thus sequence confirmation may not be essential. Alternatively, one can amplify Rab ORFs using primers containing in-frame start and stop codons and 5′ restriction enzyme sites for use in traditional cloning. This strategy was used successfully to express isolate MB38-1 (rab-8 CA) within ciliated neurons to disrupt vesicle transport to the cilia [67].

### The Importance of Research-based Lab Courses

As mentioned previously, the toolkit developed here was created in the context of a research-based lab course. One clear benefit of this pedagogical approach is its ability to provide an authentic research experience to a large number of students. While the traditional master-apprenticeship model has been successful it can exclude many due to infrastructure limitations of the host institution and large numbers of biology students. As a result, participants in extracurricular research typically involve a small number of self-selected students. Specifically, these students are aware that research opportunities exist, are highly motivated to participate, can afford to volunteer time outside of the classroom and fully appreciate its value.

To complement the traditional approach, many science educators now suggest bringing an authentic research experience into the classroom [13]–[15], [101]. Proponents of this approach argue that research-based lab courses can capture and possibly inspire the largest number of students, including those who had never envisioned a career in research [102]–[105]. This demand has been echoed by the National Science Education Standards that urge STEM disciplines to alter or replace cookbook lab courses for ones that emphasize inquiry, discovery and the development of a research mindset [106]. Moreover, the Committee on Undergraduate Biology Education to Prepare Research Scientists for the 21st Century argue that research-based lab courses are valuable because they are inherently interdisciplinary [107]. Not only do students gain scientific knowledge, but they also gain experience with experimental design, quantitative analysis and written and oral communication. By bringing scientific research into the classroom, educators demonstrate that scientists deal with unanswered questions on a daily bases and help students develop skills that are difficult to teach including critical thinking and scientific reasoning.

In a research-based laboratory course, ideally each student (or student pair) is provided a unique project where the outcome and path to completion is unknown, even to the course instructor. As described by Weaver et al. [13], there should be “no information in any textbook, laboratory manual or journal article about their expected results.” Individual students should have numerous opportunities to make decisions in experimental design and execution, in data analysis and in forming conclusions. While guidance can be provided by both peers and the instructor, ultimate success is the sole responsibility of the individual or student pair.

In practice, research-based curriculum can be logistically complex and expensive. It can be difficult to come up with a large number of unique projects year after year, to prep the lab with equipment and reagents to support all ongoing projects not to mention supervising a large number of inexperienced, wet lab scientists doing research. These challenges are particularly daunting at institutions that lack the funds to hire teaching assistants to help course instructors.

I (co-author M.G.) initiated a research-based lab course in 2007 that attempted to maximize the benefits of research-based curricula but minimize the challenges. To this end, I exploited the availability of the *C. elegans* ORFeome resource, a collection of ORF entry clones corresponding to the majority of ORFs identified within the C. elegans genome [11], [12]. In brief, each student was given an entry clone from the ORFeome library as starting material (kindly provided by Kang Shen). From there each student took an interdisciplinary approach to accomplish the aims outlined in Figure 5. Specifically, all students learned wet lab skills beneficial to the molecular biologist but also had to master online databases, sequence analysis tools and additional software including GenBank, PubMed, OMIM, BLAST, Clustal W, A Plasmid Editor (M. Wayne Davis), Image J (NIH), Excel and PowerPoint (Microsoft).

The use of the ORFeome library as starting material combined with the creation of two mutant forms for each gene was instrumental in overcoming many of the challenges outlined above. At the beginning of each course, student pairs were assigned a unique gene. Importantly each student still conducted his/her own work but this strategy allowed for the creation of backup reagents, as we quickly discovered that some DNA isolates were unusable. Then each student was assigned a unique mutant form allowing the opportunity to work independently. Importantly, during both phases of the course, students used a similar set of computer tools and molecular techniques in any given week allowing for conservation of reagents and instruction. Moreover, the projects were similar enough that peer review could be used to double check experimental design and/or interpretation of results so that this task was not left entirely up to the instructor. In fact, once peer review became a formal part of the course, the number of student and instructor errors declined dramatically (unpublished data). Finally, the use of ORFeome clones allowed ample opportunity for discovery. For example, during the process of purifying and analyzing “wild type” isolates students discovered a new splice form and identified and characterized ORFeome clone errors. In addition, students took pride in the knowledge that they were creating new reagents for the scientific community, work that might get published in a peer-reviewed journal. In fact, the possibility of publication had the most dramatic impact on both the student and the instructor in terms of creating an authentic research experience. As the instructor, I cared deeply that the lab presentations were clear, results were analyzed correctly and reagents and lab notebooks were organized and documented properly. We were united in our effort to produce and document our research accurately.

For science educators interested in designing a similar course to this one or others that have been described recently [105], [108]–[110], Gateway-compatible ORFeome libraries are now available for numerous species in addition to *C. elegans* including humans and *Schizosaccharomyces pombe* among others [111]–[117]. Within these libraries there are many genes and/or gene families that could benefit from tools allowing *in vitro* or *in vivo* analysis of dominant negative and/or constitutive active forms. For example, transcription factors can often be converted to dominant negative forms by deleting protein-protein interaction domains while leaving DNA binding domains intact [118]. Protein kinases can often be converted to dominant, kinase-dead forms by mutating the universally conserved lysine residue within the ATP-binding domain [119]–[121]. Alternatively, one can alter kinase effectors by mutating putative phosphorylation sites to mimic dephosphorylation and/or constitutive phosphorylation [122]. These types of projects would be particularly useful if done in collaboration with a lab interested in using the reagents upon completion. Ultimately, the creation of tools that facilitate gene function studies can help reduce the so-called bottleneck of genes that have been identified by sequence but still lack clear function.

In closing, it is important to note that this class is offered as a required class in the Biotech Certificate Program (BCP) at California State University, East Bay. It typically enrolls post baccalaureate students. To date, only a small number of undergraduates have taken the course. While I have not attempted to offer this class to undergraduates, I imagine it could be done with simple modifications. For example, I would likely provide more guidance in experimental design but leave all opportunities for data analysis and oral presentations as is. A more comprehensive description of the course and additional suggestions for implementation will be published elsewhere.

## Materials and Methods

### Rab Protein Identification

To manually identify the complete set of Rab proteins from *C. elegans*, 28 Rab proteins identified by Pereira-Leal and Seabra (2001) were first aligned by Muscle using MEGA5 [26]. Once aligned, these sequences were trimmed at the N- and C-termini to include the Rab domain plus the N-terminal RabSF1 motif. This multiple sequence alignment was then used as a query in PSI-BLAST using the bioinformatics toolkit [27]. The complete list (excluding splice variants) of significant hits identified by PSI-BLAST was aligned by Muscle again with obvious alignment errors fixed manually. The alignment of each RabF domain (1–5) was then exported to Microsoft Excel in order to calculate the RabF percent identity to consensus sequences of the five RabF motifs combined [9] (Co-author M.G.).

### Molecular Phylogenetics

To construct the phylogenetic trees of Human and Worm Rab proteins described in Figures 2, 3 and S2, a single copy of each Rab subfamily was retrieved from NCBI and combined with all *C. elegans* Rab proteins identified as described in the text. Again, to simplify the list of genes, all splice variants save one were removed. The one that remained contained a Rab prenylation motif if present. Also, all human Rab subfamily members that formed a species-specific clade with bootstrap support >99 were reduced to a single member. Then, the full-length human and worm Rab proteins were aligned by Muscle using MEGA5. Once aligned, sequences were trimmed of their variable N- and C-termini leaving sequence from RabSF1 through RabSF4 and the prenylation motif (including the first cysteine and the sequence that followed). Finally, a variety of trees were created with MEGA5 (co-author M.G.). Phylogenetic reconstruction methods used included Neighbor Joining, maximum likelihood or minimum evolution. Amino acid substitution models included Poisson, Equal Input, Jones Thornton Taylor (JTT), Whelan and Goldman (WAG) and Dayhoff. Gap deletion treatments included partial or pairwise and rates and patterns of evolution include gamma distributed (+G), invariant sites (+I) or uniform. For any given tree, the specific combination used is described in the Figure legend and text. The bootstrap test was used to calculate the percentage of replicate trees in which the associated genes cluster together (100 replicates for Maximum Likelihood and 500 for all others). The list of genes (including accession numbers) and the alignment used to create the phylogenetic trees is also provided (Figure S1).

Phylogenetic trees containing human *RAB23*, worm *ZK669.5* and a group of human and worm nonRab top hits of ZK669.5 were done as described above. The list of nonRab human genes included Ras-like protein family member 12 (NP_057647.1), RERG/RAS-like (AAH42888.1), BAB55008.1, Ras-like protein family member 11A (NP_996563.1), Ras-related associated with diabetes (AAH57815.1), GEM (NP_859053.1), RAS (RAD and GEM)-like (AAV38882.1), RAS (RAD and GEM)-like GTP binding 2 (AAH35663.1), Rap-2c (NP_067006.3), R-ras2 (NP_036382.2), Rap-1b (NP_056461.1), M-Ras (NP_001078518.1), RalA (NP_005393.2), and Rheb (NP_005605.1). The list of nonRab worm genes included Y71F9AR.2 (NP_491082.2), rap-1 (NP_501549.1), rap-2 (NP_506707.2), rheb-1 (NP_499079.1), ras-2 (NP_497972.1), ral-1 (NP_497689.1), C08F8.7 (WP:CE40190) and C44C11.1a (WP:CE24846).

The phylogenetic reconstruction in Figure 4B was created by the Maximum Likelihood method based on the Whelan and Goldman model (WAG) [123] with a discrete Gamma distribution (+G) to model evolutionary rate difference among sites (5 categories, G = 1.7896) and allowing for some sites (3.0941%) to be evolutionarily invariant (+I) (co-author M.G.). This reconstruction was suggested by the MEGA5 model test analyzing 48 different amino acid substitution models. Since gaps and variable termini were deleted during the alignment process, no further deletions were made during the reconstruction. There were a total of 167 amino acids in the final data set.

### Comparative Analysis of Intron Position

Subfamilies analyzed by intron position included *Rab31*, *Rab5*, *Rab22*, *Rab21*, *Rab23*, *Rab6* and *Rab34*. Species analyzed came mostly from the Opisthokonta including two Lophotrochozoans: *Caliptella teleta* (Ctel) and *Lottia gigantean* (Lgig); three Arthropods *Apis mellifera* (Amel), *Tribolium castaneum* (Tcas), and *Drosophila melanogaster* (Dmel); three Nematodes *Brugia malayi* (Bmal), *Caenorhabditis elegans* (Cele) and *Trichinella spiralis* (Tspi)*;* one Echinoderm, *Strongylocentrotus purpuratus* (Spur)*;* two Chordates *Ciona intestinalis* (Cint), and *Homo sapien*s (Hsap); two Cnidarians *Hydra magnipapillata* (Hmag) and *Nematostella vectensis* (Nvec)*;* one Choanozoan, *Monosiga brevicollis* (Mbre); one unranked Opisthokant, *Capsaspora owczarzaki* (Cowc)*;* and two intron-rich fungal species [85], *Coprinopsis cinerea okayama* (Ccin) and *Phanerochaete chrysosporium* (Pchr). For the *Rab31* subfamily analysis, one additional species came from the Amoebazoan, *Dictyostelium discoideum* (Ddis) and two additional species came from the supergroup Chromalveolata, phylum Ciliophora: *Ichthyophthirius multifiliis* (Imul) and *Tetrahymena thermophile* (Tthe). Species substitutions were necessary on three occasions where gene sequence was of low quality or could not be found by the methods employed here. Specifically, alternate Hymenopterans, *Apis florea* (Aflo) and Camponotus floridanus (Cflo) substituted for *Apis mellifera Rab5 and Rab34,* respectively. An alternate dipteran, *Anopheles gambiae* (Agam), substituted for *Drosophila melanogaster Rab34*. Members of each subfamily were identified from the species listed above through a “sequence space hopping” strategy previously described [53]. When possible, reciprocal best hits were identified by searching the reference sequence database at Genbank using default parameters in Basic Local Alignment Search Tool (BLAST) at NCBI otherwise the nonredundant (nr) protein database was searched. Sequence for *Lottia gigantea*, *Capitella teleta* and *Nematostella vectensis* were obtained by BLAST at the Joint Genome Institute (JGI). The complete list of accession numbers corresponding to the sequences used in the comparative analysis of intron position is provided in Figure S4.

Members of each subfamily were first aligned independently using Muscle in MEGA5. Within each subfamily, species-specific insertions (present in only one species) were deleted along with nonconserved terminal regions. Terminal regions chosen for deletion failed to produce stable alignments, contained gaps involving multiple species and consistently fell below an arbitrary overall 50% amino acid identity cutoff. Subfamily alignments were then sequentially combined into one file and realigned with each new addition by Muscle in MEGA5. The MSA was then exported to excel. This alignment was used to create the ML reconstruction in Figure 4B and for the intron position analysis (co-author M.G.).

Intron positions were determined using a variety of methods depending on sequence type. For reference sequences from NCBI (i.e. XP_ and NP_), SPLIGN was used (NCBI). For nonreference sequences from NCBI (i.e. EFW_) an annotated text map of the exon intron boundaries was created by A plasmid Editor (Wayne Davis) from the Genbank file (.gb) corresponding to the gene of interest. For sequences from the JGI database, an annotated text map of the 3-frame translation for each gene model was examined (co-author M.G.). Intron position information was then mapped onto the excel file with intron phase information retained (Figure S4). For analysis of intron conservation, intron position was defined as conserved if the 5′ and/or 3′ splice site was at an identical position and phase in at least two species.

### Monte Carlo Simulations of Intron Positions

Two types of Monte Carlo simulations were performed using Python scripts created in TextWrangler (co-author S.B.). One compares a gene of interest to a single set of subfamily specific conserved intron positions (SSCIPs). It analyzes the likelihood that a specific number of intron positions of a given Rab might match the set of SSCIPs by chance. This python script randomly generates X whole numbers (nonrepeating) within the range from 1 to Y, for the gene and subfamily of interest, where X is the number of introns and Y the number of possible intron insertion sites in that particular species (protosplice sites). For each replicate, data sets corresponding to each are freshly generated and compared. Numbers that occur in both data sets in a pairwise comparison is indicative of a shared intron insertion site or a coincidence. When complete the Monte Carlo simulation provides the sum of all types of coincidences observed. In other words, the number of times 0, 1, 2, 3 etc. coincidences were observed in a single pairwise comparison. This script requires the following inputs: 1) The number of introns present in the Rab gene of interest, 2) the number of SSCIPs present in the Rab subfamily of interest, 3) the hypothetical number of protosplice sites in the pairwise alignment and 4) the total number of replicates performed (100,000). Assuming that all nucleotide positions within the alignment are potential intron insertion sites, the protosplice site number was set at 501. Assuming that protosplice sites are present on average every 7 nucleotides [51], the protosplice site number was set at 72.

Another Monte Carlo simulation analyzes the likelihood that intron insertion sites randomly match across N number of species, each containing a specific number of introns (co-author S.B.). The above-mentioned algorithm was scaled up to perform “multiwise” comparisons of N species, for this purpose. For each replicate, randomly generated data sets corresponding to each of the N species are compared and the number of shared intron positions that involve exactly 2, 3, 4, 5, etc. species are counted. When complete, this Monte Carlo simulation provides the total number of instances where conserved intron positions involved exactly 2, 3, 4, 5 etc. species. This script requires the following inputs: 1) The number of introns present in each Rab gene analyzed in the MSA, 2) the hypothetical number of protosplice sites present within the MSA (see above) and 3) the total number of replicates performed (100,000).

The output created by either method was used to estimate P values (co-author S.B.). Specifically, P values were estimated using the formula, P value = r/n where n equals the number of replicates and r equals the number of instance where a specific value *equal to or greater than* a specific value of interest (i.e. the number of times that *at least* 7 species possessed a shared intron position) was observed [124].

### Site Directed Mutagenesis

To create mutant clones by site directed mutagenesis, the published protocol for Quikchange II XL was followe (Agilent Technologies, Cat # 200522) with a few noted exceptions (all co-authors excluding M.G.). Specifically, desalted primers were instead of PAGE purified with no loss in efficiency (data not shown). In addition, half the suggested reaction mix was used. To generate mutagenic primers that might create or destroy a restriction enzyme site the now obsolete program, Primer Generator [125] was initially used. Now SiteFind [126] is used to create restriction enzyme sites (where possible) and “A plasmid Editor” is used to destroy restriction enzyme sites (where possible) [127]. Once a mismatch region is chosen, primers are designed manually by student co-authors following the criteria outlined in the Quikchange protocol except that the Tm is calculated with the equation designed for creating insertions or deletions (Tm = 81.5+0.41(%GC)-675/N) where N = primer length (not including the mismatch region) and percent GC is a whole number. Again, the mismatch region is ignored. Importantly, the mismatch region is defined as the number of nucleotides that are different from the WT sequence including internal nucleotides that might otherwise match. For example, AG**TTT**GA has a mismatch of 3 even though the WT sequence is AG**CTC**GA.

### GenBank Accession Numbers

The accession numbers for full-length ORFeome clone sequences of WT rab isolates described in the text and Table 1 are as follows: IK3-1 = JQ235180, GC5-1 = JQ235181, AP2-1 = JQ235182, PD3-1 = JQ235183, SDS6-1 = JQ235184, AV3-2 = JQ235185, MG2-1 = JQ235186, MB5-2 = JQ235187, MB10-1 = JQ235188, ZY2-2 = JQ235189, SP6-1 = JQ235190, NM7-2 = JQ235191, LAK5-1 = JQ235192, JP6-1 =  JQ235193, SVP6-1 = JQ235194, MEL4-1 = JQ235195, TR6-1 = JQ235196, RV3-1 = JQ235197, DVD8-2 = JQ235198, LGK3-2 = JQ235199, KK9-2 = JQ235200, EB8-2 = JQ235201.

## Supporting Information

Figure S1 **The multiple sequence alignment used to create the tree described in** **Figures 2** **and S2.**
(XLS)

Figure S2 **The cladogram in** **figure 2** **shown as an unrooted phlyogram with relative branch lengths restored.**
(EPS)

Figure S3 **Individual clusters from** **Figure 4B** **enlarged with each branch labeled with species name and accession number.**
(PDF)

Figure S4 **Intron positions of Rab subfamily members within the conserved portion of the multiple sequence alignment (MSA).** Yellow squares correspond to phase 1 introns. Green squares correspond to phase 2 introns and red squares correspond to phase 3 introns (intron is positioned *after* the indicated codon). Intron free columns within the MSA were deleted. Numbering in the top row corresponds to the amino acid position of Rab6 from *Lottia gigantea*. For species abbreviations, see Figure 4 legend. Stars mark the position of each SSCIP as defined in the text. Black stars correspond to SSCIPs that are statistically significant at P(Monte Carlo <0.00001). Dark gray stars correspond to SSCIPs that are statistically significant at P(Monte Carlo <0.05). Light gray stars correspond to SSCIPs that are not statistically significant.
(PDF)

Figure S5 **Full-length ORFeome Clone Sequence (FlOCS) for each isolate (WT, DN and CA) described in** **Table 1** **.**
(DOC)

Table S1 **A list of mutagenic primers (forward only) used in site-directed mutagenesis of mutant Rab forms.** The mismatch region is highlighted in bold and all caps. For a definition of mismatch and the equation used to calculate Tm, see methods. Where applicable, the diagnostic enzyme and the form it digests is indicated. m = mutant, wt = wild type.
(DOC)
