# Supplementary figures and images for: The C. elegans Rab Family: Identification, Classification and Toolkit Construction
Source: PLoS One. 2012 Nov 21;7(11):e49387. doi: 10.1371/journal.pone.0049387 (PMC3504004; doi:10.1371/journal.pone.0049387)

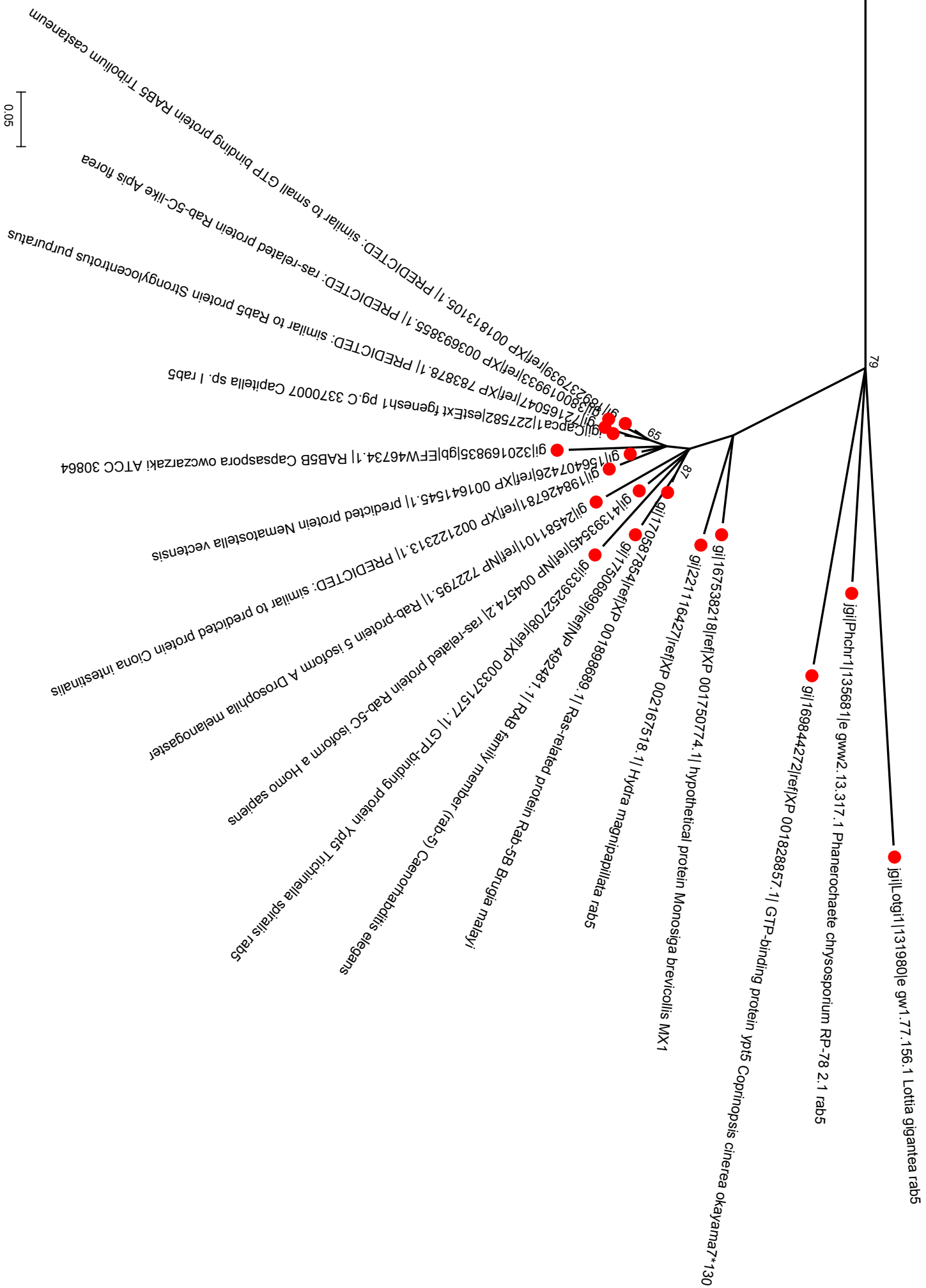

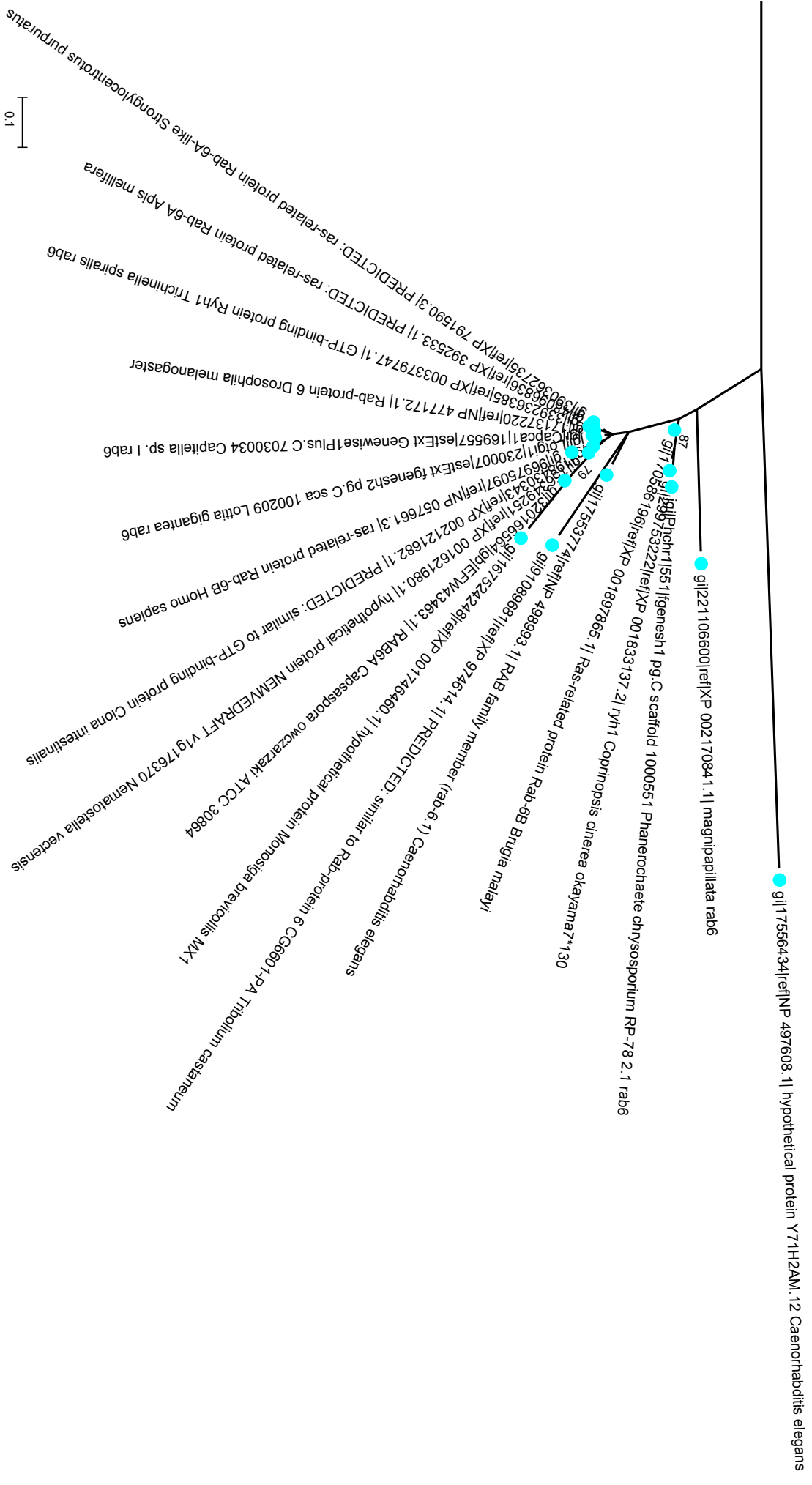

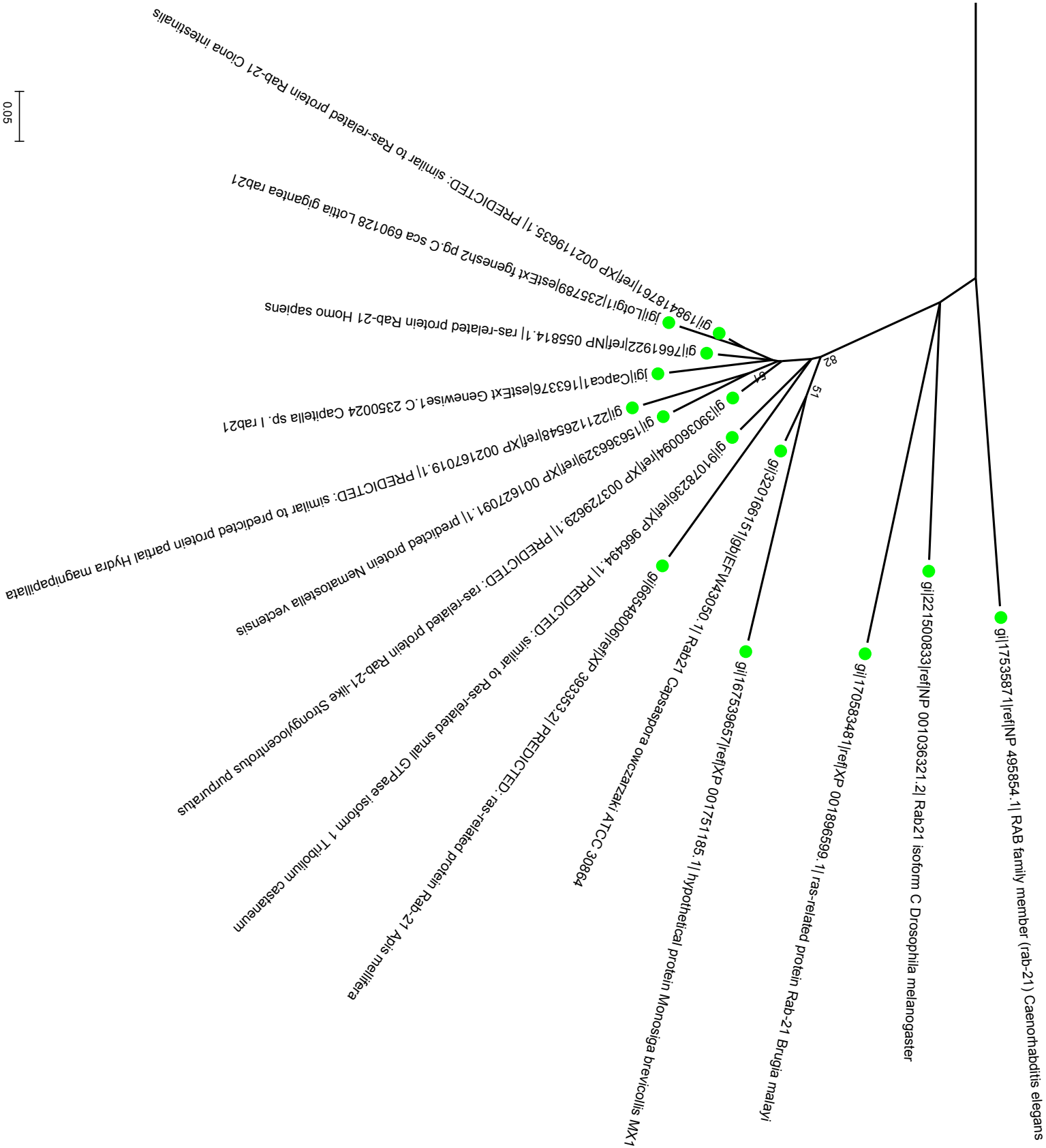

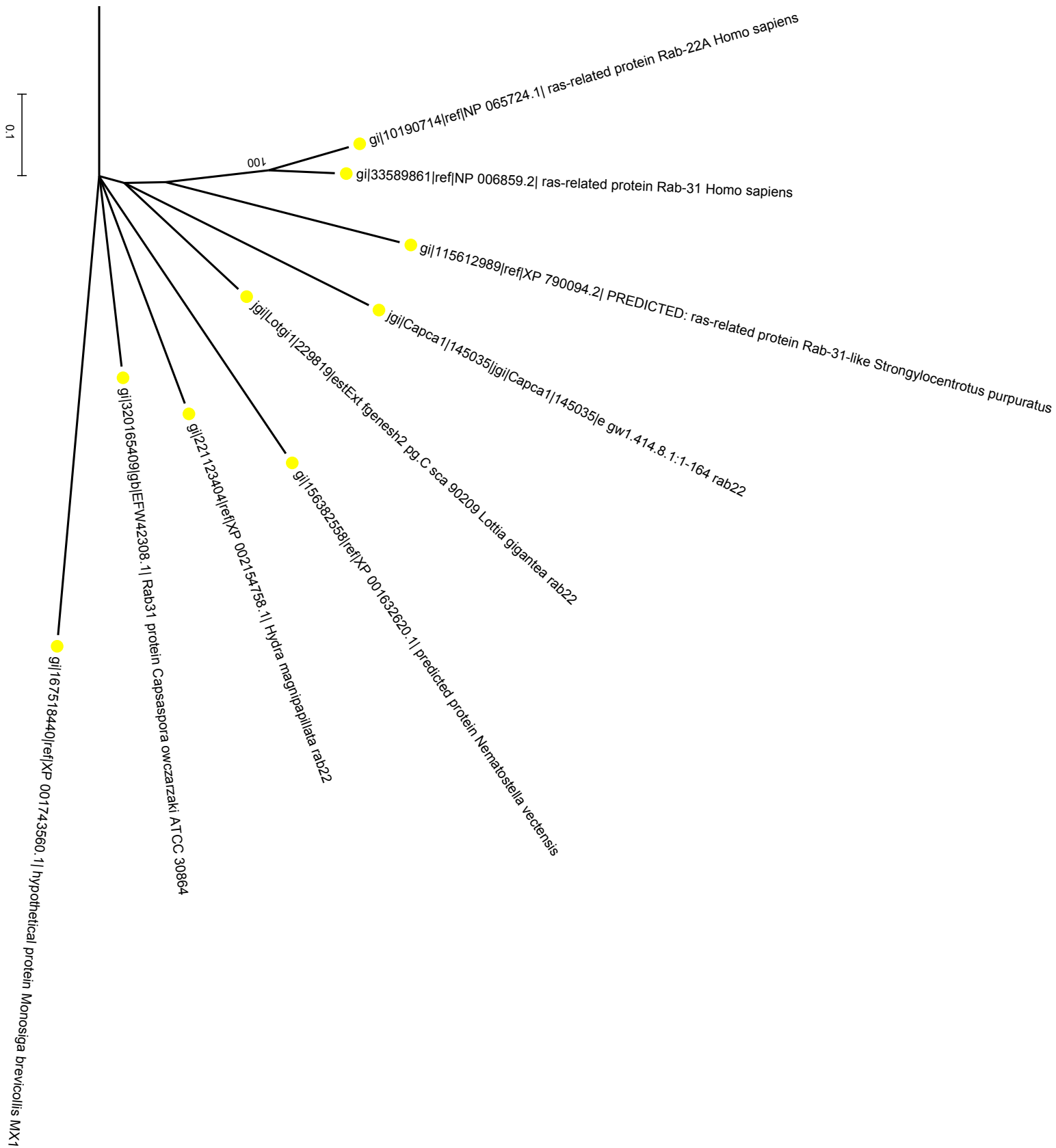

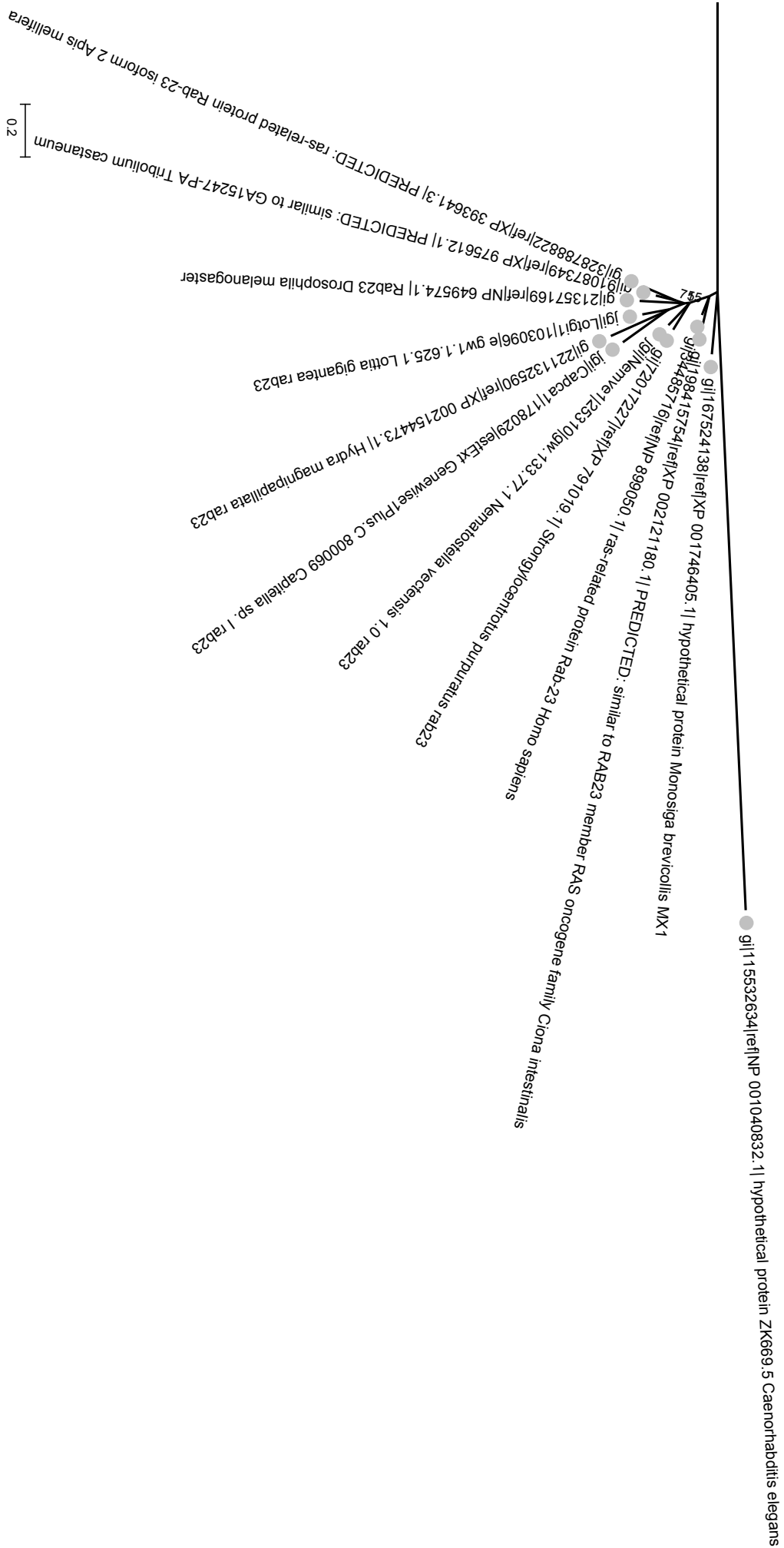

0.1

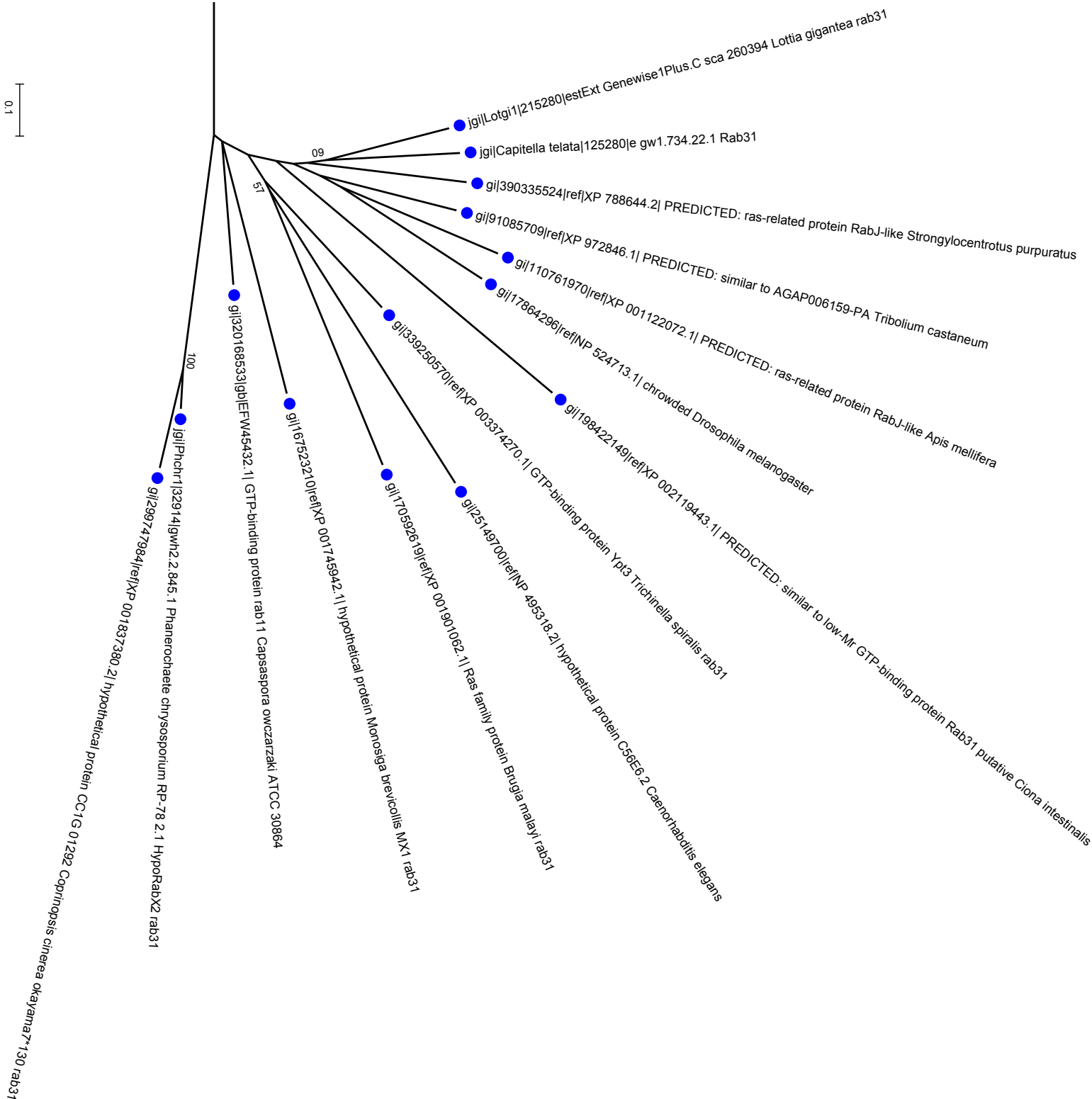

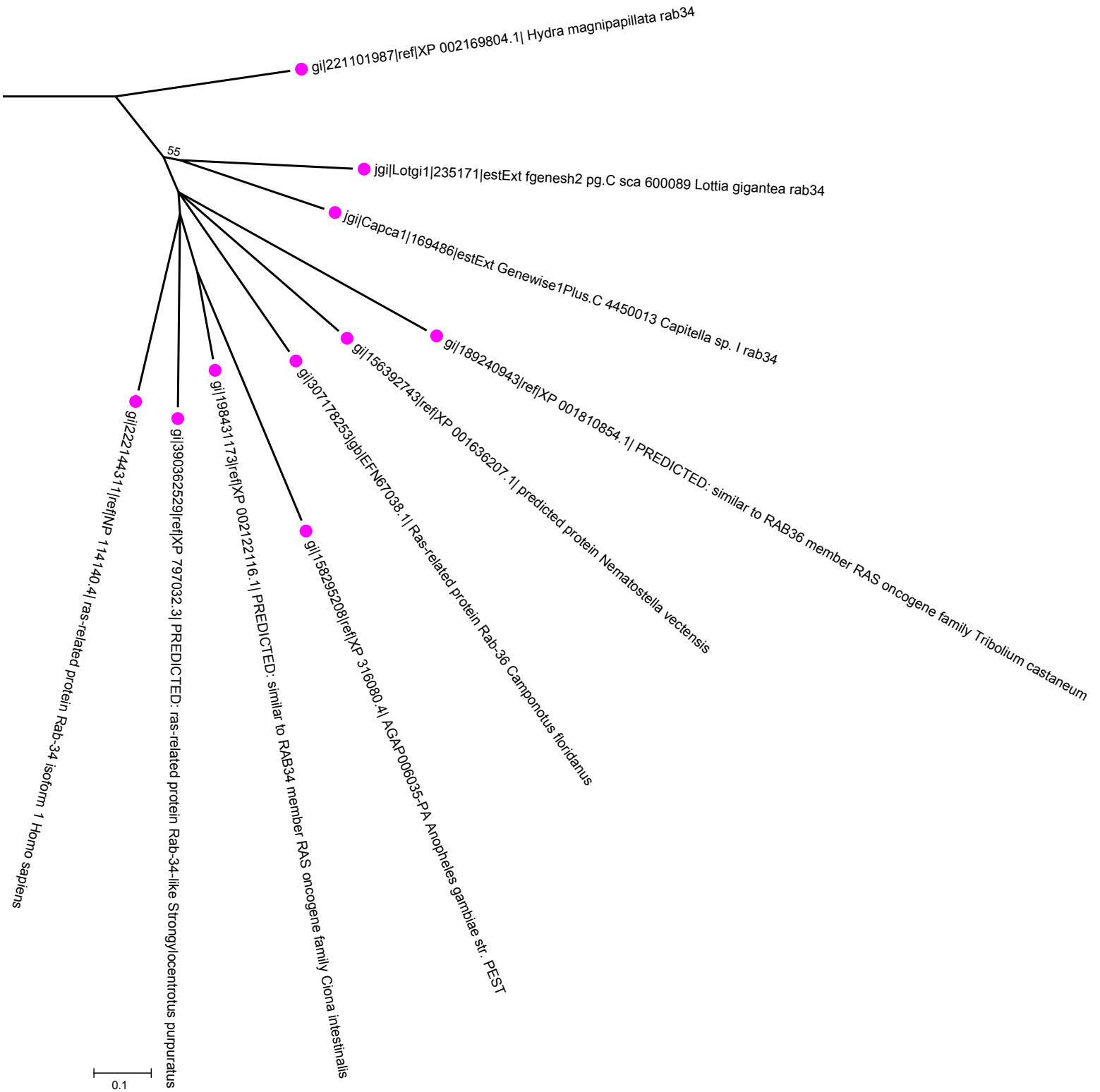

Supplement: Figure S3 — Individual clusters from Figure 4B enlarged with each branch labeled with species name and accession number. (PDF) [file pone.0049387.s003.pdf]

**Figure S4.**

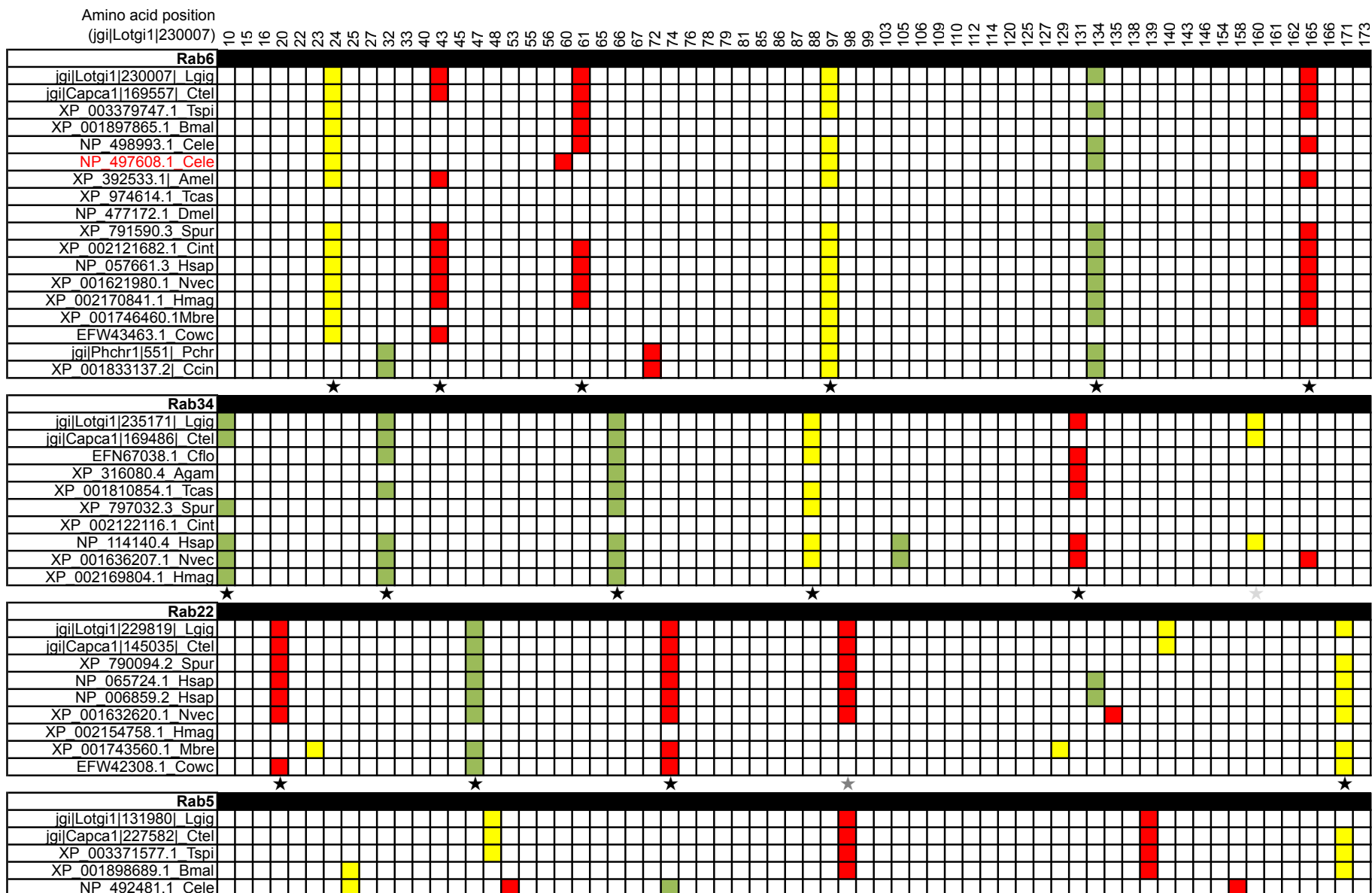



[illegible]

Supplement: Figure S4 — Intron positions of Rab subfamily members within the conserved portion of the multiple sequence alignment (MSA). Yellow squares correspond to phase 1 introns. Green squares correspond to phase 2 introns and red squares correspond to phase 3 introns (intron is positioned after the indicated codon). Intron free columns within the MSA were deleted. Numbering in the top row corresponds to the amino acid position of Rab6 from Lottia gigantea. For species abbreviations, see Figure 4 legend. Stars mark the position of each SSCIP as defined in the text. Black stars correspond to SSCIPs that are statistically significant at P(Monte Carlo <0.00001). Dark gray stars correspond to SSCIPs that are statistically significant at P(Monte Carlo <0.05). Light gray stars correspond to SSCIPs that are not statistically significant. (PDF) [file pone.0049387.s004.pdf]
