# Supplementary material for: The C. elegans Rab Family: Identification, Classification and Toolkit Construction
Source: PLoS One. 2012 Nov 21;7(11):e49387. doi: 10.1371/journal.pone.0049387 (PMC3504004; doi:10.1371/journal.pone.0049387)
Supplement: Figure S5 — Full-length ORFeome Clone Sequence (FlOCS) for each isolate (WT, DN and CA) described in Table 1 . (DOC) [file pone.0049387.s005.doc]

**Figure S5.**

>FlOCS_IK3-1_rab-1(WT)

GGGCCCAATAATGATTTTATTTTGACTGATAGTGACCTGTTCGTTGCAACAAATTGATGAGCAATGCTTTTTTATAATGCCAACTTTGTACAAAAAAGCAGGCTTGGCAGCAATGAACCCTGAATACGACTACCTCTTCAAACTCCTCCTCATCGGAGATTCTGGCGTCGGAAAATCGTGCCTGTTGCTGCGTTTCGCCGATGACACATACACTGAATCATACATCTCCACTATCGGAGTTGACTTTAAAATCCGTACTATTGAGCTCGACGGCAAAACTATCAAGCTTCAAATCTGGGACACTGCTGGTCAGGAACGTTTCCGCACAATTACATCGAGCTACTACCGCGGAGCTCACGGAATTATCGTTGTCTACGATATCACTGACCAAGAGACATTCAACAACGTGAAGCAGTGGCTTCAGGAAATCGATAGGTACGCATGTGAGAACGTCAACAAGCTGTTGGTCGGAAACAAATGCGATTTGACAGCGAAGAGAGCTGTGGAGACTCAAGCTGCCCAGGATTATGCTGGACAACTCGGAATCCCGTTCCTCGAGACATCCGCCAAGTCATCGACGAATGTTGAACAGGCCTTCCTTACCATGGCTTCGGAAATCAAATCACGTATGGGACCAGTTCAAGGAGCCGGAGGTGCTCCAGGTGTGCGTATCACGGGAAGTCAACCGGTTCAAGACAAGAAGAGCGGTGGATGTTGTCACCCAGCTTTCTTGTACAAAGTTGGCATTATAAGAAAGCATTGCTTATCAATTTGTTGCAACGAACAGGTCACTATCAGTCAAAATAAAATCATTATTTGCCATCCAGCTGCAGCTCTGGCCCGTGTCTCAAAATCTCTGATGTTACATTGCACAAGATAAAAATATATCATCATGAACAATAAAACTGTCTGCTTACATAAACAGTAATACAAGGGGTGTTATGAGCCATATTCAACGGGAAACGTCGAGGCCGCGATTAAATTCCAACATGGATGCTGA

>FlOCS_IK20-1_rab-1(DN)

TTCGTTGCAACAAATTGATGAGCAATGCTTTTTTATAATGCCAACTTTGTACAAAAAAGCAGGCTTGGCAGCAATGAACCCTGAATACGACTACCTCTTCAAACTCCTCCTCATCGGAGATTCTGGCGTCGGAAAAAACTGCCTGTTGCTGCGTTTCGCCGATGACACATACACTGAATCATACATCTCCACTATCGGAGTTGACTTTAAAATCCGTACTATTGAGCTCGACGGCAAAACTATCAAGCTTCAAATCTGGGACACTGCTGGTCAGGAACGTTTCCGCACAATTACATCGAGCTACTACCGCGGAGCTCACGGAATTATCGTTGTCTACGATATCACTGACCAAGAGACATTCAACAACGTGAAGCAGTGGCTTCAGGAAATCGATAGGTACGCATGTGAGAACGTCAACAAGCTGTTGGTCGGAAACAAATGCGATTTGACAGCGAAGAGAGCTGTGGAGACTCAAGCTGCCCAGGATTATGCTGGACAACTCGGAATCCCGTTCCTCGAGACATCCGCCAAGTCATCGACGAATGTTGAACAGGCCTTCCTTACCATGGCTTCGGAAATCA

>FlOCS_PP50-1_rab-1(CA)

ATGATTTTATTTTGACTGATAGTGACCTGTTCGTTGCAACAAATTGATGAGCAATGCTTTTTTATAATGCCAACTTTGTACAAAAAAGCAGGCTTGGCAGCAATGAACCCTGAATACGACTACCTCTTCAAACTCCTCCTCATCGGAGATTCTGGCGTCGGAAAATCGTGCCTGTTGCTGCGTTTCGCCGATGACACATACACTGAATCATACATCTCCACTATCGGAGTTGACTTTAAAATCCGTACTATTGAGCTCGACGGCAAAACTATCAAGCTTCAAATCTGGGACACTGCTGGTCTCGAACGTTTCCGCACAATTACATCGAGCTACTACCGCGGAGCTCACGGAATTATCGTTGTCTACGATATCACTGACCAAGAGACATTCAACAACGTGAAGCAGTGGCTTCAGGAAATCGATAGGTACGCATGTGAGAACGTCAACAAGCTGTTGGTCGGAAACAAATGCGATTTGACAGCGAAGAGAGCTGTGGAGACTCAAGCTGCCCAGGATTATGCTGGACAACTCGGAATCCCGTTCCTCGAGACATCCGCCAAGTCATCGACGAATGTTGAACAGGCCTTCCTTACCATGGCTTCGGAAATCAAATCACGTATGGGACCAGTTCAAGGAGCCGGAGGTGCTCCAGGTGTGCGTATCACGGGAAGTCAACCGGTTCAAGACAAGAAGAGCGGTGGATGTTGTCACCCAGCTTTCTTGTACAAAGTTGGCATTATAAGAAAGCATTGCTTATCAATTTGTTGCAACGAACAGGTCACTATCAGTCAAAATAAAATCATTATTTGCCATCCAGCTGCAGCTCTGGCCCGTGTCTCAAAATCTCTGATGTTACATTGCACAAGATAAAAATATATCATCATGAACAATAAAACTGTCTGCTTACATAAACAGTAATAC

>FlOCS_GC5-1_rab-2(WT)

CGGGCCCAATAATGATTTTATTTTGACTGATAGTGACCTGTTCGTTGCAACAAATTGATGAGCAATGCTTTTTTATAATGCCAACTTTGTACAAAAAAGCAGGCTTGTCATATGCCTACCTTTTCAAGTACATTATCATCGGGGATACTGGAGTAGGAAAATCCTGCTTGCTCCTTCAGTTTACCGACAAACGTTTCCAGCCAGTTCATGATTTGACGATTGGCGTCGAATTCGGAGCCCGTATGGTGACAATTGACGGAAAGCAGATCAAACTTCAAATTTGGGACACAGCCGGACAAGAATCATTCCGCTCCATCACTCGTTCCTATTATCGTGGAGCCGCCGGAGCTCTTCTCGTCTACGACATTACACGACGCGACACATTCAATCATTTGACATCTTGGCTTGAGGATGCCAGGCAGCACAGTAATTCCAATATGGTTATTATGTTGATTGGAAATAAGAGTGACCTGGAAGCCCGTCGCGAAGTGAAACGTGAAGAGGGAGAAGCATTCGCACGAGAGCACGGACTCGTATTCATGGAGACATCTGCCAAGACGGCTGCCAACGTGGAAGAGGCGTTCATCGACACTGCCAAAGAGATCTACCGTAAGATTCAAGAAGGCGTGTTCGACATTAACAATGAGGCAAACGGAATCAAGTTGGGACCACAGCACTCTCCAAGCTCACCAAATTCTCCAGGTGGAAATGCGACGGGAGGATTGGGCGGTGGATCTGGATGCTGTTACCCAGCTTTCTTGTACAAAGTTGGCATTATAAGAAAGCATTGCTTATCAATTTGTTGCAACGAACAGGTCACTATCAGTCAAATAAAATCATTATTTGCCATCCAGCTGCAGCTCTGGCCCGTGTCTCAAAATCTCTGATGTTACATTGCACAAGATAAAAATATATCATCATGAACAATAAAACTGTCTGCTTACATAAACAGTAATACAAGGGGGGTTATGAGCCATATTCACGGGAAACGTC

>FlOCS_GC33-2_rab-2(DN)

AATGATTTTATTTTGACTGATAGTGACCTGTTCGTTGCAACAAATTGATGAGCAATGCTTTTTTATAATGCCAACTTTGTACAAAAAAGCAGGCTTGTCATATGCCTACCTTTTCAAGTACATTATCATCGGGGATACTGGAGTAGGAAAAAACTGCTTGCTCCTTCAGTTTACCGACAAACGTTTCCAGCCAGTTCATGATTTGACGATTGGCGTCGAATTCGGAGCCCGTATGGTGACAATTGACGGAAAGCAGATCAAACTTCAAATTTGGGACACAGCCGGACAAGAATCATTCCGCTCCATCACTCGTTCCTATTATCGTGGAGCCGCCGGAGCTCTTCTCGTCTACGACATTACACGACGCGACACATTCAATCATTTGACATCTTGGCTTGAGGATGCCAGGCAGCACAGTAATTCCAATATGGTTATTATGTTGATTGGAAATAAGAGTGACCTGGAAGCCCGTCGCGAAGTGAAACGTGAAGAGGGAGAAGCATTCGCACGAGAGCACGGACTCGTATTCATGGAGACATCTGCCAAGACGGCTGCCAACGTGGAAGAGGCGTTCATCGACACTGCCAAAGAGATCTACCGTAAGATTCAAGAAGGCGTGTTCGACATTAACAATGAGGCAAACGGAATCAAGTTGGGACCACAGCACTCTCCAAGCTCACCAAATTCTCCAGGTGGAAATGCGACGGGAGGATTGGGCGGTGGATCTGGATGCTGTTACCCAGCTTTCTTGTACAAAGTTGGCATTATAAGAAAGCATTGCTTATCAATTTGTTGCAACGAACAGGTCACTATCAGTCAAAATAAAATCATTATTTGCCATCCAGCTGCA

>FlOCS_SM15-1_rab-2(CA)

AATGATTTTATTTTGACTGATAGTGACCTGTTCGTTGCAACAAATTGATGAGCAATGCTTTTTTATAATGCCAACTTTGTACAAAAAAGCAGGCTTGTCATATGCCTACCTTTTCAAGTACATTATCATCGGGGATACTGGAGTAGGAAAATCCTGCTTGCTCCTTCAGTTTACCGACAAACGTTTCCAGCCAGTTCATGATTTGACGATTGGCGTCGAATTCGGAGCCCGTATGGTGACAATTGACGGAAAGCAGATCAAACTTCAAATTTGGGACACAGCCGGCCTAGAATCATTCCGCTCCATCACTCGTTCCTATTATCGTGGAGCCGCCGGAGCTCTTCTCGTCTACGACATTACACGACGCGACACATTCAATCATTTGACATCTTGGCTTGAGGATGCCAGGCAGCACAGTAATTCCAATATGGTTATTATGTTGATTGGAAATAAGAGTGACCTGGAAGCCCGTCGCGAAGTGAAACGTGAAGAGGGAGAAGCATTCGCACGAGAGCACGGACTCGTATTCATGGAGACATCTGCCAAGACGGCTGCCAACGTGGAAGAGGCGTTCATCGACACTGCCAAAGAGATCTACCGTAAGATTCAAGAAGGCGTGTTCGACATTAACAATGAGGCAAACGGAATCAAGTTGGGACCACAGCACTCTCCAAGCTCACCAAATTCTCCAGGTGGAAATGCGACGGGAGGATTGGGCGGTGGATCTGGATGCTGTTACCCAGCTTTCTTGTACAAAGTTGGCATTATAAGAAAGCATTGCTTATCAATTTGTTGCAACGAACAGGTCACTATCAGTCAAAATAAAATCATTATTTGCCATCCAGCTGCAGCTCTGGCCCGTGTCTCAAAATCTCTGATGTTACATTGCACAAGATAAAAATATATCATCATGAACAATAAAACTGTCTGCTTACATAAACAGTAATAC

>FlOCS_AP2-1_rab-3(WT)

CGGGCCCAATAATGATTTTATTTTGACTGATAGTGACCTGTTCGTTGCAACAAATTGATGAGCAATGCTTTTTTATAATGCCAACTTTGTACAAAAAAGCAGGCTTGGCGGCTGGCGGACAACCTCAAGGCGCTACACCGGGACAACCCGATCAGAACTTTGACTACATGTTCAAGCTCCTGATAATCGGAAATTCATCAGTTGGAAAAACATCATTCCTCTTCCGTTACTGTGATGATTCATTCACTTCTGCCTTCGTCTCTACTGTCGGAATCGATTTCAAAGTGAAAACTGTGTTCCGTGGAGACAAACGAGTCAAACTTCAAATCTGGGATACCGCCGGACAGGAGAGGTACCGTACCATCACCACCGCCTACTATCGTGGAGCAATGGGATTCATTCTGATGTATGACATCACTAATGAAGAGTCTTTTAATAGTGTTCAGGATTGGTGCACTCAAATCAAGACATACTCATGGGAAAATGCTCAAGTTGTTTTGGTTGGAAATAAATGTGATATGGACTCTGAAAGAGTTGTATCTATGGATAGGGGACGCCAACTTGCTGATCAACTTGGTTTGGAATTCTTCGAAACATCAGCCAAGGAGAACATTAATGTAAAGGCAGTTTTTGAGAAGTTGGTGGAGATTATTTGTGATAAGATGGCAGAGAGTTTGGATAAGGACCCACAGCAACAGCCAAAAGGACAGAAGCTCGAAGCGAATCCGACCCAAAAGCCTGCTCAACAGCAATGCAATTGCTACCCAGCTTTCTTGTACAAAGTTGGCATTATAAGAAAGCATTGCTTATCAATTTGTTGCAACGAACAGGTCACTATCAGTCAAAATAAAATCATTATTTGCCATCCAGCTGCAGCTCTGGCCCGTGTCTCAAAATCTCTGATGTTACATTGCACAAGATAAAAATATATCATC

>FlOCS_NG33-1_rab-3(DN)

CAATAATGATTTTATTTTGACTGATAGTGACCTGTTCGTTGCAACAAATTGATGAGCAATGCTTTTTTATAATGCCAACTTTGTACAAAAAAGCAGGCTTGGCGGCTGGCGGACAACCTCAAGGCGCTACACCGGGACAACCCGATCAGAACTTTGACTACATGTTCAAGCTCCTGATAATCGGAAATTCATCAGTTGGAAAGAATTCATTCCTCTTCCGTTACTGTGATGATTCATTCACTTCTGCCTTCGTCTCTACTGTCGGAATCGATTTCAAAGTGAAAACTGTGTTCCGTGGAGACAAACGAGTCAAACTTCAAATCTGGGATACCGCCGGACAGGAGAGGTACCGTACCATCACCACCGCCTACTATCGTGGAGCAATGGGATTCATTCTGATGTATGACATCACTAATGAAGAGTCTTTTAATAGTGTTCAGGATTGGTGCACTCAAATCAAGACATACTCATGGGAAAATGCTCAAGTTGTTTTGGTTGGAAATAAATGTGATATGGACTCTGAAAGAGTTGTATCTATGGATAGGGGACGCCAACTTGCTGATCAACTTGGTTTGGAATTCTTCGAAACATCAGCCAAGGAGAACATTAATGTAAAGGCAGTTTTTGAGAAGTTGGTGGAGATTATTTGTGATAAGATGGCAGAGAGTTTGGATAAGGACCCACAGCAACAGCCAAAAGGACAGAAGCTCGAAGCGAATCCGACCCAAAAGCCTGCTCAACAGCAATGCAATTGCTACCCAGCTTTCTTGTACAAAGTTGGCATTATAAGAAAGCATTGCTTATCAATTTGTTGCAACGAACAGGTCACTATCAGTCAAAATAAAATCATTATTTGCCATCCAGCTGCAGCTCTGGCCCGTGTCTCAAATCTCTGATGTTACATTGCACAAGATAAAAATATATCATCATGAACAATAAAACTGTCTGCTTACATAACAGTAATACA

>FlOCS_CG7-1_rab-3(CA)

AATGATTTTATTTTGACTGATAGTGACCTGTTCGTTGCAACAAATTGATGAGCAATGCTTTTTTATAATGCCAACTTTGTACAAAAAAGCAGGCTTGGCGGCTGGCGGACAACCTCAAGGCGCTACACCGGGACAACCCGATCAGAACTTTGACTACATGTTCAAGCTCCTGATAATCGGAAATTCATCAGTTGGAAAAACATCATTCCTCTTCCGTTACTGTGATGATTCATTCACTTCTGCCTTCGTCTCTACTGTCGGAATCGATTTCAAAGTGAAAACTGTGTTCCGTGGAGACAAACGAGTCAAACTTCAAATCTGGGATACCGCCGGACTCGAGAGGTACCGTACCATCACCACCGCCTACTATCGTGGAGCAATGGGATTCATTCTGATGTATGACATCACTAATGAAGAGTCTTTTAATAGTGTTCAGGATTGGTGCACTCAAATCAAGACATACTCATGGGAAAATGCTCAAGTTGTTTTGGTTGGAAATAAATGTGATATGGACTCTGAAAGAGTTGTATCTATGGATAGGGGACGCCAACTTGCTGATCAACTTGGTTTGGAATTCTTCGAAACATCAGCCAAGGAGAACATTAATGTAAAGGCAGTTTTTGAGAAGTTGGTGGAGATTATTTGTGATAAGATGGCAGAGAGTTTGGATAAGGACCCACAGCAACAGCCAAAAGGACAGAAGCTCGAAGCGAATCCGACCCAAAAGCCTGCTCAACAGCAATGCAATTGCTACCCAGCTTTCTTGTACAAAGTTGGCATTATAAGAAAGCATTGCTTATCAATTTGTTGCAACGAACAGGTCACTATCAGTCAAAATAAAATCATTATTTGCCATCCAGCTGCAGCTCTGGCCCGTGTCTCAAAATCTCTGATGTTACATTGCACAAGATAAAAATATATCATCATGAACAATAAAACTGTCTGCTTACATAAACAGTAATACA

>FlOCS_PD3-1_rab-5(WT)

AATAATGATTTTATTTTGACTGATAGTGACCTGTTCGTTGCAACAAATTGATGAGCAATGCTTTTTTATAATGCCAACTTTGTACAAAAAAGCAGGCTTGGCCGCCCGAAACGCAGGAACCGCCCGGCCTGGAGGTCCAAACAGAACGTGCCAGTTCAAACTCGTTCTTCTCGGAGAAAGTGCTGTAGGCAAATCATCTCTCGTATTGCGATTCGTCAAGGGACAATTTCACGAGTATCAGGAGTCGACTATAGGTGCTGCGTTCCTCACTCAAACTGTGTGCCTTGACGATGCAACAATTAAATTCGAAATCTGGGATACTGCAGGACAAGAAAGATATCATTCATTGGCACCAATGTACTATCGAGGAGCTCAAGCGGCTATTGTTGTCTACGATATTACCAATCAAGAATCTTTCCAAAAAGCGAAAAATTGGGTAAAAGAACTGCAGCGACAGGCATCTCCAAATATTGTGATGGCATTGGCTGGAAACAAGGCTGATGTTGCCAATAAGCGAACTGTTGAGTATGAAGAAGCTAATGCTTATGCTGAGGACAATGCTCTCTTGTTCATGGAGACATCTGCGAAGACCTCAATGAACGTGAACGATATTTTCATGGCAATAGCTAAAAAGTTGCCAATCGGACCAGCACAGGGTGAACCAACGGGAACTGTCGATATGAACCAACCACAACAGCAACAAAAGGGTTCATGCTGTAAATACCCAGCTTTCTTGTACAAAGTTGGCATTATAAGAAAGCATTGCTTATCAATTTGTTGCAACGAACAGGTCACTATCAGTCAAAATAAAATCATTATTTGCCATCCAGCTGCAGCTCTGGCCCGTGTCTCAAAATCTCTGATGTTACATTGCACAAGATAAAAATATATCATCATGAACAATAAAACTGTCTGCTTACATAAACAGTAATACAAGGGTGTTATGAGCCATATTCAACGGGAAACGTCGAGGCCGCGATTAAATTCCAACATGG

>FlOCS_NH11-1_rab-5(DN)

GATTTTATTTTGACTGATAGTGACCTGTTCGTTGCAACAAATTGATGAGCAATGCTTTTTTATAATGCCAACTTTGTACAAAAAAGCAGGCTTGGCCGCCCGAAACGCAGGAACCGCCCGGCCTGGAGGTCCAAACAGAACGTGCCAGTTCAAACTCGTTCTTCTCGGAGAAAGTGCTGTAGGCAAGAATTCTCTCGTATTGCGATTCGTCAAGGGACAATTTCACGAGTATCAGGAGTCGACTATAGGTGCTGCGTTCCTCACTCAAACTGTGTGCCTTGACGATGCAACAATTAAATTCGAAATCTGGGATACTGCAGGACAAGAAAGATATCATTCATTGGCACCAATGTACTATCGAGGAGCTCAAGCGGCTATTGTTGTCTACGATATTACCAATCAAGAATCTTTCCAAAAAGCGAAAAATTGGGTAAAAGAACTGCAGCGACAGGCATCTCCAAATATTGTGATGGCATTGGCTGGAAACAAGGCTGATGTTGCCAATAAGCGAACTGTTGAGTATGAAGAAGCTAATGCTTATGCTGAGGACAATGCTCTCTTGTTCATGGAGACATCTGCGAAGACCTCAATGAACGTGAACGATATTTTCATGGCAATAGCTAAAAAGTTGCCAATCGGACCAGCACAGGGTGAACCAACGGGAACTGTCGATATGAACCAACCACAACAGCAACAAAAGGGTTCATGCTGTAAATACCCAGCTTTCTTGTACAAAGTTGGCATTATAAGAAAGCATTGCTTATCAATTTGTTGCAACGAACAGGTCACTATCAGTCAAAATAAAATCATTATTTGCCATCCAGCTGCAGCTCTGGCCCGTGTCTCAAAATCTCTGATG

>FlOCS_MJ21-1_rab-5(CA)

ATTTTGACTGATAGTGACCTGTTCGTTGCAACAAATTGATGAGCAATGCTTTTTTATAATGCCAACTTTGTACAAAAAAGCAGGCTTGGCCGCCCGAAACGCAGGAACCGCCCGGCCTGGAGGTCCAAACAGAACGTGCCAGTTCAAACTCGTTCTTCTCGGAGAAAGTGCTGTAGGCAAATCATCTCTCGTATTGCGATTCGTCAAGGGACAATTTCACGAGTATCAGGAGTCGACTATAGGTGCTGCGTTCCTCACTCAAACTGTGTGCCTTGACGATGCAACAATTAAATTCGAAATCTGGGATACTGCAGGACTCGAAAGATATCATTCATTGGCACCAATGTACTATCGAGGAGCTCAAGCGGCTATTGTTGTCTACGATATTACCAATCAAGAATCTTTCCAAAAAGCGAAAAATTGGGTAAAAGAACTGCAGCGACAGGCATCTCCAAATATTGTGATGGCATTGGCTGGAAACAAGGCTGATGTTGCCAATAAGCGAACTGTTGAGTATGAAGAAGCTAATGCTTATGCTGAGGACAATGCTCTCTTGTTCATGGAGACATCTGCGAAGACCTCAATGAACGTGAACGATATTTTCATGGCAATAGCTAAAAAGTTGCCAATCGGACCAGCACAGGGTGAACCAACGGGAACTGTCGATATGAACCAACCACAACAGCAACAAAAGGGTTCATGCTGTAAATACCCAGCTTTCTTGTACAAAGTTGGCATTATAAGAAAGCATTGCTTATCAATTTGTTGCAACGAACAGGTCACTATCAGTCAAAATAAAATCATTATTTGCCATCCAGCTGCAGCTCTGGCCCGTGTCTCAA

>FlOCS_SDS6-1_rab-6.1(WT)

GATTTTATTTTGACTGATAGTGACCTGTTCGTTGCAACAAATTGATGAGCAATGCTTTTTTATAATGCCAACTTTGTACAAAAAAGCAGGCTTGGCTGATTTCACAAATAACGCTCTCAAAAAGTTTAAGCTCGTTTTTCTCGGCGAACAGAGTGTGGGAAAAACTTCTATCATCACCAGATTCATGTATGATTCGTTTGACAACACCTATCAAGCCACCATTGGAATTGACTTTCTCAGCAAGACTATGTATCTTGAAGACAGAACCATTCGCCTTCAATTGTGGGATACCGCTGGCCAGGAACGTTTCCGATCTTTGATTCCATCTTACATTCGTGATTCGAGTGTTGCCGTTGTCGTGTATGATATTACTAATGCCAATTCCTTCCATCAAACAACAAAGTGGGTTGATGATGTTCGCAACGAACGTGGATGTGACGTCATTATCGTGCTCGTCGGAAATAAAACCGATTTGGCTGATAAAAGACAAGTTTCGACGGAGGATGGAGAAAAGAAGGCTCGCGATTTGAATGTGATGTTCATTGAAACTTCTGCAAAAGCTGGCTACAACGTCAAACAGCTCTTCCGCAAAATCGCTACGGCACTTCCAGGAATTGTCCAAGAGGAGACGCCTGAGCAGCCCAACATCGTTATCATGAATCCTCCTAAAGACGCGGAAGAGAGCCAAGGCCGTCAATGTCCGTGTTACCCAGCTTTCTTGTACAAAGTTGGCATTATAAGAAAGCATTGCTTATCAATTTGTTGCAACGAACAGGTCACTATCAGTCAAAATAAAATCATTATTTGCCATCCAGCTGCAGCTCTGGCCCGTGTCTCAAAATCTCTGATGTTACATTGCACAAGATAAAAATATATCATCATGAACAATAAAACTGTCTGCTTACATAAACAGTAATACA

>FlOCS_PRS33-2_rab-6.1(DN)

GATTTTATTTTGACTGATAGTGACCTGTTCGTTGCAACAAATTGATGAGCAATGCTTTTTTATAATGCCAACTTTGTACAAAAAAGCAGGCTTGGCTGATTTCACAAATAACGCTCTCAAAAAGTTTAAGCTCGTTTTTCTCGGCGAACAGAGTGTGGGAAAGAATTCTATCATCACCAGATTCATGTATGATTCGTTTGACAACACCTATCAAGCCACCATTGGAATTGACTTTCTCAGCAAGACTATGTATCTTGAAGACAGAACCATTCGCCTTCAATTGTGGGATACCGCTGGCCAGGAACGTTTCCGATCTTTGATTCCATCTTACATTCGTGATTCGAGTGTTGCCGTTGTCGTGTATGATATTACTAATGCCAATTCCTTCCATCAAACAACAAAGTGGGTTGATGATGTTCGCAACGAACGTGGATGTGACGTCATTATCGTGCTCGTCGGAAATAAAACCGATTTGGCTGATAAAAGACAAGTTTCGACGGAGGATGGAGAAAAGAAGGCTCGCGATTTGAATGTGATGTTCATTGAAACTTCTGCAAAAGCTGGCTACAACGTCAAACAGCTCTTCCGCAAAATCGCTACGGCACTTCCAGGAATTGTCCAAGAGGAGACGCCTGAGCAGCCCAACATCGTTATCATGAATCCTCCTAAAGACGCGGAAGAGAGCCAAGGCCGTCAATGTCCGTGTTACCCAGCTTTCTTGTACAAAGTTGGCATTATAAGAAAGCATTGCTTATCAATTTGTTGCAACGAACAGGTCACTATCAGTCAAAATAAAATCATTATTTGCCATCCAGCTGCAGCTCTGGCCCGTGTCTCAAAATCTCTGATGTTACATTGCACAAGATAAAAATATATCATCATGAACAATAAAACTGTCTGCTTACATAAACAGTAATAC

>FlOCS_SDS34-1_rab-6.1(CA)

GATTTTATTTTGACTGATAGTGACCTGTTCGTTGCAACAAATTGATGAGCAATGCTTTTTTATAATGCCAACTTTGTACAAAAAAGCAGGCTTGGCTGATTTCACAAATAACGCTCTCAAAAAGTTTAAGCTCGTTTTTCTCGGCGAACAGAGTGTGGGAAAAACTTCTATCATCACCAGATTCATGTATGATTCGTTTGACAACACCTATCAAGCCACCATTGGAATTGACTTTCTCAGCAAGACTATGTATCTTGAAGACAGAACCATTCGCCTTCAATTGTGGGATACCGCTGGCTTAGAACGTTTCCGATCTTTGATTCCATCTTACATTCGTGATTCGAGTGTTGCCGTTGTCGTGTATGATATTACTAATGCCAATTCCTTCCATCAAACAACAAAGTGGGTTGATGATGTTCGCAACGAACGTGGATGTGACGTCATTATCGTGCTCGTCGGAAATAAAACCGATTTGGCTGATAAAAGACAAGTTTCGACGGAGGATGGAGAAAAGAAGGCTCGCGATTTGAATGTGATGTTCATTGAAACTTCTGCAAAAGCTGGCTACAACGTCAAACAGCTCTTCCGCAAAATCGCTACGGCACTTCCAGGAATTGTCCAAGAGGAGACGCCTGAGCAGCCCAACATCGTTATCATGAATCCTCCTAAAGACGCGGAAGAGAGCCAAGGCCGTCAATGTCCGTGTTACCCAGCTTTCTTGTACAAAGTTGGCATTATAAGAAAGCATTGCTTATCAATTTGTTGCAACGAACAGGTCACTATCAGTCAAAATAAAATCATTATTTGCCATCCAGCTGCAGCTCTGGCCCGTGTCTCAAAATCTCTGATGTTACATTGCACAAGATAAAAATATATCATCATGAACAATAAAACTGTCTGCTTACATAAACAGTAATAC

>FlOCS_AV3-2_rab-6.2(WT)

GATTTTATTTTGACTGATAGTGACCTGTTCGTTGCAACAAATTGATGAGCAATGCTTTTTTATAATGCCAACTTTGTACAAAAAAGCAGGCTTGTCGGACTTTGGTAATCCGTTGAAGAAATTCAAGCTTGTGTTCCTCGGGGAACAAAGTGTCGGAAAAACCTCCCTAATCACTCGCTTCATGTACGACTCCTTCGACAACACCTACCAGGCCACCATCGGAATCGACTTCCTGAGTAAGACCATGTACTTGGAGGACCGAACCGTTCGTCTTCAACTTTGGGACACCGCTGGACAGGAACGTTTCCGCTCACTCATCCCATCCTACATCCGTGATTCTACAGTTGCTGTTGTGGTTTATGACATCACCAACTCCAACTCTTTCCATCAAACTTCCAAGTGGATTGACGACGTCCGTACTGAGCGTGGAAGTGATGTAATCATCATGTTGGTCGGCAACAAGACCGATCTTTCCGATAAGAGGCAAGTCACCACCGACGAAGGAGAGAGAAAGGCCAAGGAGCTTAATGTGATGTTCATCGAGACTTCTGCCAAGGCCGGCTACAATGTGAAGCAGCTTTTCCGTCGTATCGCCGGAGCCCTACCAGGAATCATCAAGGACGACCCAGTGGAGCCACCAAATGTGGTCACGATGGACCCAATCCGTCAGCGTCAAATTGTCACCGACGAGGGATCGTGCTGGTGCTACCCAGCTTTCTTGTAAAAGTTGGCATTATAAGAAAGCATTGCTTATCAATTTGTTGCAACGAACAGGTCACTATCAGTCAAAATAAAATCATTATTTGCCATCCAGCTGCAGCTCTGGCCCGTGTCTCAAAATCTCTGATGTTACATTGCACAAGATAAAAATATATCATCATGAACAATAAAACTGTCTGCTTACATAAACAGTAATAC

>FlOCS_NJ16-1_rab-6.2(DN)

ATGATTTTATTTTGACTGATAGTGACCTGTTCGTTGCAACAAATTGATGAGCAATGCTTTTTTATAATGCCAACTTTGTACAAAAAAGCAGGCTTGTCGGACTTTGGTAATCCGTTGAAGAAATTCAAGCTTGTGTTCCTCGGGGAACAAAGTGTCGGAAAAAACTCCCTAATCACTCGCTTCATGTACGACTCCTTCGACAACACCTACCAGGCCACCATCGGAATCGACTTCCTGAGTAAGACCATGTACTTGGAGGACCGAACCGTTCGTCTTCAACTTTGGGACACCGCTGGACAGGAACGTTTCCGCTCACTCATCCCATCCTACATCCGTGATTCTACAGTTGCTGTTGTGGTTTATGACATCACCAACTCCAACTCTTTCCATCAAACTTCCAAGTGGATTGACGACGTCCGTACTGAGCGTGGAAGTGATGTAATCATCATGTTGGTCGGCAACAAGACCGATCTTTCCGATAAGAGGCAAGTCACCACCGACGAAGGAGAGAGAAAGGCCAAGGAGCTTAATGTGATGTTCATCGAGACTTCTGCCAAGGCCGGCTACAATGTGAAGCAGCTTTTCCGTCGTATCGCCGGAGCCCTACCAGGAATCATCAAGGACGACCCAGTGGAGCCACCAAATGTGGTCACGATGGACCCAATCCGTCAGCGTCAAATTGTCACCGACGAGGGATCGTGCTGGTGCTACCCAGCTTTCTTGTAAAAGTTGGCATTATAAGAAAGCATTGCTT

>FlOCS_AV11-1_rab-6.2(CA)

GGGCCCAATAATGATTTTATTTTGACTGATAGTGACCTGGTTCGTTGCAACAAATTGATGAGCAATGCTTTTTTATAATGCCAACTTTGTACAAAAAAGCAGGCTTGTCGGACTTTGGTAATCCGTTGAAGAAATTCAAGCTTGTGTTCCTCGGGGAACAAAGTGTCGGAAAAACCTCCCTAATCACTCGCTTCATGTACGACTCCTTCGACAACACCTACCAGGCCACCATCGGAATCGACTTCCTGAGTAAGACCATGTACTTGGAGGACCGAACCGTTCGTCTTCAACTTTGGGACACCGCTGGACTGGAACGTTTCCGCTCACTCATCCCATCCTACATCCGTGATTCTACAGTTGCTGTTGTGGTTTATGACATCACCAACTCCAACTCTTTCCATCAAACTTCCAAGTGGATTGACGACGTCCGTACTGAGCGTGGAAGTGATGTAATCATCATGTTGGTCGGCAACAAGACCGATCTTTCCGATAAGAGGCAAGTCACCACCGACGAAGGAGAGAGAAAGGCCAAGGAGCTTAATGTGATGTTCATCGAGACTTCTGCCAAGGCCGGCTACAATGTGAAGCAGCTTTTCCGTCGTATCGCCGGAGCCCTACCAGGAATCATCAAGGACGACCCAGTGGAGCCACCAAATGTGGTCACGATGGACCCAATCCGTCAGCGTCAAATTGTCACCGACGAGGGATCGTGCTGGTGCTACCCAGCTTTCTTGTACAAAGTTGGCATTATAAGAAAGCATTGCTTATCAATTTGTTGCAACGAACAGGTCACTATCAGTCAAAATAAAATCATTATTTGCCATCCAGCTGCAGCTCTGGCCCGTGTCTCAAATCTCTGATGTTACAT

>FlOCS_MG2-1_rab-7(WT)

ATTTTGACTGATAGTGACCTGTTCGTTGCAACAAATTGATGAGCAATGCTTTTTTATAATGCCAACTTTGTACAAAAAAGCAGGCTTGTCGGGAACCAGAAAGAAGGCGCTGCTCAAAGTGATCATTCTCGGAGATTCGGGCGTTGGAAAAACATCTTTGATGAATCAATATGTAAATCGGCGATTTAGTAATCAATATAAGGCAACGATTGGAGCCGACTTCCTCACACGCGACGTAAACATTGACGACAGAACCGTCACTCTTCAGATCTGGGATACAGCCGGCCAGGAACGTTTCCAATCGCTCGGAGTCGCTTTTTATCGTGGAGCCGATTGCTGTGTGCTGGCTTTTGACGTCACAAATGCCGCCTCGTTCAAATCGCTTGACTCGTGGCGTGACGAGTTTCTGATTCAGGCTAGCCCCCGCGATCCAGACCATTTCCCGTTTGTCCTGCTCGGAAACAAGGTGGATTTGGAGTCACAGCGTGCCGTTTCGTCAAAACGTGCTCAATCTTGGTGCCAGACGAAGGGAAATATTCCGTATTACGAGGTTTCTGCCAAGGAAGCTCTCAACGTTGAAGCCGCATTTTTGGCAATTGCTCGTGATGCGTTGGCTAGAGAATCTCAGGAAACCAACGATTTCCCAGAGTTTCCCGACCAAATCCGTCTCAACCCAAATCAACAGAACCAGCAGAATTCGGGATGCAATTGTTACCCAGCTTTCTTGTACAAAGTTGGCATTATAAGAAAGCATTGCTTATCAATTTGTTGCAACGAACAGGTCACTATCAGTCAAAATAAAATCATTATTTGCCATCCAGCTGCAGCTCTGGCCCGTGTCTCAAAATCTCTGATGTTACATTGCACAAGATAAAAATATATCATC

>FlOCS_SI46-4_rab-7(DN)

GATTTTATTTTGACTGATAGTGACCTGTTCGTTGCAACAAATTGATGAGCAATGCTTTTTTATAATGCCAACTTTGTACAAAAAAGCAGGCTTGTCGGGAACCAGAAAGAAGGCGCTGCTCAAAGTGATCATTCTCGGAGATTCGGGCGTTGGAAAGAATTCTTTGATGAATCAATATGTAAATCGGCGATTTAGTAATCAATATAAGGCAACGATTGGAGCCGACTTCCTCACACGCGACGTAAACATTGACGACAGAACCGTCACTCTTCAGATCTGGGATACAGCCGGCCAGGAACGTTTCCAATCGCTCGGAGTCGCTTTTTATCGTGGAGCCGATTGCTGTGTGCTGGCTTTTGACGTCACAAATGCCGCCTCGTTCAAATCGCTTGACTCGTGGCGTGACGAGTTTCTGATTCAGGCTAGCCCCCGCGATCCAGACCATTTCCCGTTTGTCCTGCTCGGAAACAAGGTGGATTTGGAGTCACAGCGTGCCGTTTCGTCAAAACGTGCTCAATCTTGGTGCCAGACGAAGGGAAATATTCCGTATTACGAGGTTTCTGCCAAGGAAGCTCTCAACGTTGAAGCCGCATTTTTGGCAATTGCTCGTGATGCGTTGGCTAGAGAATCTCAGGAAACCAACGATTTCCCAGAGTTTCCCGACCAAATCCGTCTCAACCCAAATCAACAGAACCAGCAGAATTCGGGATGCAATTGTTACCCAGCTTTCTTGTACAAAGTTGGCATTATAAGAAAGCATTGCTTATCAATTTGTTGCAACGAACAGGTCACTATCAGTCAAAATAAAATCATTATTTGCCATCCA

>FlOCS_SI28-5_rab-7(CA)

GATGATATATTTTTATCTTGTGCAATGTAACATCAGAGATTTTGAGACACGGGCCAGAGCTGCAGCTGGATGGCAAATAATGATTTTATTTTGACTGATAGTGACCTGTTCGTTGCAACAAATTGATAAGCAATGCTTTCTTATAATGCCAACTTTGTACAAGAAAGCTGGGTAACAATTGCATCCCGAATTCTGCTGGTTCTGTTGATTTGGGTTGAGACGGATTTGGTCGGGAAACTCTGGGAAATCGTTGGTTTCCTGAGATTCTCTAGCCAACGCATCACGAGCAATTGCCAAAAATGCGGCTTCAACGTTGAGAGCTTCCTTGGCAGAAACCTCGTAATACGGAATATTTCCCTTCGTCTGGCACCAAGATTGAGCACGTTTTGACGAAACGGCACGCTGTGACTCCAAATCCACCTTGTTTCCGAGCAGGACAAACGGGAAATGGTCTGGATCGCGGGGGCTAGCCTGAATCAGAAACTCGTCACGCCACGAGTCAAGCGATTTGAACGAGGCGGCATTTGTGACGTCAAAAGCCAGCACACAGCAATCGGCTCCACGATAAAAAGCGACTCCGAGCGATTGGAAACGTTCCAAGCCGGCTGTATCCCAGATCTGAAGAGTGACGGTTCTGTCGTCAATGTTTACGTCGCGTGTGAGGAAGTCGGCTCCAATCGTTGCCTTATATTGATTACTAAATCGCCGATTTACATATTGATTCATCAAAGATGTTTTTCCAACGCCCGAATCTCCGAGAATGATCACTTTGAGCAGCGCCTTCTTTCTGGTTCCCGACAAGCCTGCTTTTTTGTACAAAGTTGGCATTATAAAAAA

>FlOCS_MB5-2_rab-8(WT)

ATGCTTTTTTATAATGCCAACTTTGTACAAAAAAGCAGGCTTGGCAAAAACTTACGACTACTTGTTTAAGCTCTTGCTTATTGGAGACAGTGGAGTCGGCAAGACGTGCGTACTGTTCCGGTTCTCAGATGATTCGTTCAATAACTCGTTCATTTCAACAATTGGAATCGACTTCAAAATCCGTACGATTGAGCTCGATGGGAAGAAAATCAAACTTCAAATTTGGGACACAGCCGGGCAGGAGAGATTCAGAACTATCACAACGGCTTATTACCGCGGTGCTATGGGAATCATTCTGGTGTACGATATCACCAACGAGCGATCATTTGAAAACATCAAAAACTGGATTCGCAATATAGAAGAGCATGCGGCTTCGGATGTCGAGAGGATGATCATCGGCAACAAATGTGATATTGAAGAACGCCGTGAAGTGTCGCGGGACCGTGGAGAGCAGCTCGCAATCGAGTACGGCACGAAATTCCTGGAAACGTCGGCGAAGGCCAATCTGAATATTGATGAAGCTTTCTTCACACTGGCTCGTGATATCAAGAGCAAGATGGAGCAGAACGAGATGCGTGCCGGTGGATCAGTTTCGAATACGGGTCGTGTGAACGTCGGTGGATCGGGGACACAGAAGAAGAGCTTCTTCAGCAACTGGAGCTGCAATTTGCTTTACCCAGCTTTCTTGTACAAAGTTGGCATTATAAGAAAGCATTGCTTATCAATTTGTTGCAACGAACAGGTCACTATCAGTCAAAATAAAATCCATTATTTGCCATCCCAGCTGCAGCTCTGGGCCCGTGTCTCAAAAATCTCTGATGTTACATTGCCACAA

>FlOCS_DK26-1_rab-8(DN)

TGATTTTATTTTGACTGATAGTGACCTGTTCGTTGCAACAAATTGATGAGCAATGCTTTTTTATAATGCCAACTTTGTACAAAAAAGCAGGCTTGGCAAAAACTTACGACTACTTGTTTAAGCTCTTGCTTATTGGAGACAGTGGAGTCGGCAAGAATTGCGTACTGTTCCGGTTCTCAGATGATTCGTTCAATAACTCGTTCATTTCAACAATTGGAATCGACTTCAAAATCCGTACGATTGAGCTCGATGGGAAGAAAATCAAACTTCAAATTTGGGACACAGCCGGGCAGGAGAGATTCAGAACTATCACAACGGCTTATTACCGCGGTGCTATGGGAATCATTCTGGTGTACGATATCACCAACGAGCGATCATTTGAAAACATCAAAAACTGGATTCGCAATATAGAAGAGCATGCGGCTTCGGATGTCGAGAGGATGATCATCGGCAACAAATGTGATATTGAAGAACGCCGTGAAGTGTCGCGGGACCGTGGAGAGCAGCTCGCAATCGAGTACGGCACGAAATTCCTGGAAACGTCGGCGAAGGCCAATCTGAATATTGATGAAGCTTTCTTCACACTGGCTCGTGATATCAAGAGCAAGATGGAGCAGAACGAGATGCGTGCCGGTGGATCAGTTTCGAATACGGGTCGTGTGAACGTCGGTGGATCGGGGACACAGAAGAAGAGCTTCTTCAGCAACTGGAGCTGCAATTTGCTTTACCCAGCTTTCTTGTACAAAGTTGGCATTATAAGAAAGCATTGCTTATCAATTTGTTGCAACGAACAGGTCACTATCAGTCAAAATAAAATCATTATTTGCCATCCAGCTGCAGCTCTGGCCCGTGTCTCAAAATCTCTGATGTTACATTGCACAAGATAAAAATATATCATCATGAACAATAAAACTGTCTGCTTACATAAACAGTAATACAAGGGGTGTTATGAGCCATATTCAACGGGAAACGTCGAGGCCGCGATTAAATTCCAACATGGATGCTGATTTATATGGG

>FlOCS_MB38-1_rab-8(CA)

TGATTTTATTTTGACTGATAGTGACCTGTTCGTTGCAACAAATTGATGAGCAATGCTTTTTTATAATGCCAACTTTGTACAAAAAAGCAGGCTTGGCAAAAACTTACGACTACTTGTTTAAGCTCTTGCTTATTGGAGACAGTGGAGTCGGCAAGACGTGCGTACTGTTCCGGTTCTCAGATGATTCGTTCAATAACTCGTTCATTTCAACAATTGGAATCGACTTCAAAATCCGTACGATTGAGCTCGATGGGAAGAAAATCAAACTTCAAATTTGGGACACAGCCGGCCTGGAGAGATTCAGAACTATCACAACGGCTTATTACCGCGGTGCTATGGGAATCATTCTGGTGTACGATATCACCAACGAGCGATCATTTGAAAACATCAAAAACTGGATTCGCAATATAGAAGAGCATGCGGCTTCGGATGTCGAGAGGATGATCATCGGCAACAAATGTGATATTGAAGAACGCCGTGAAGTGTCGCGGGACCGTGGAGAGCAGCTCGCAATCGAGTACGGCACGAAATTCCTGGAAACGTCGGCGAAGGCCAATCTGAATATTGATGAAGCTTTCTTCACACTGGCTCGTGATATCAAGAGCAAGATGGAGCAGAACGAGATGCGTGCCGGTGGATCAGTTTCGAATACGGGTCGTGTGAACGTCGGTGGATCGGGGACACAGAAGAAGAGCTTCTTCAGCAACTGGAGCTGCAATTTGCTTTACCCAGCTTTCTTGTACAAAGTTGGCATTATAAGAAAGCATTGCTTATCAATTTGTTGCAACGAACAGGTCACTATCAGTCAAAATAAAATCATTATTTGCCATCCAGCTGCAGCTCTGGCCCGTGTCTCAAAATCTCTGATGTTACATTGCACAAGATAAAAATATATCATCATGAACAATAAAACTGTCTGCTTACATAAACAGTAATAC

>FlOCS_MB10-1_rab-10(WT)

GATTTTATTTTGACTGATAGTGACCTGTTCGTTGCAACAAATTGATGAGCAATGCTTTTTTATAATGCCAACTTTGTACAAAAAAGCAGGCTTGGCTCGCCGACCGTATGACATGCTCTTCAAATTGCTGCTAATAGGCGACTCAGGAGTTGGAAAAACTTGCATTCTGTACAGATTTTCGGATGATGCCTTTAACACCACATTCATCTCAACTATTGGAATCGACTTCAAAATTAAAACTATCGAATTAAAAGGAAAAAAGATTAAATTACAAATCTGGGACACAGCTGGACAAGAACGATTCCACACAATCACAACATCATATTACCGCGGAGCAATGGGAATTATGCTGGTTTACGATATCACAAATGCGAAAAGTTTTGACAATATTGCAAAGTGGTTGCGCAATATTGACGAACACGCATCGGAAGATGTTGTTAAAATGATATTGGGAAATAAATGTGATATGAGTGATAGACGAGTTGTGAGCAGAGAACGAGGAGAGAAGATCGCACAAGATCATGGTATTAGCTTCCACGAGACTTCAGCTAAATTGAATGTTCACGTCGATACAGCGTTTTACGATTTAGCAGAAGCAATCCTGGCTAAGATGCCTGATTCCACTGACGAGCAATCCCGCGATACGGTGAATCCAGTGCAACCACAGAGGCAGAGTAGCAGTGGAGGATGCTGCCACCCAGCTTTCTTGTACAAAGTTGGCATTATAAGAAAGCATTGCTTATCAATTTGTTGCAACGAACAGGTCACTATCAGTCAAAATAAAATCATTATTTGCCATCCAGCTGCAGCTCTGGCCCGTGTCTCAAAATCTCTGATGTTACATTGCACAAGATAAAAATATATCATCATGAACAATAAAACTGTCTGCTTACATAAACAGTAATAC

>FlOCS_SBA42-1_rab-10(DN)

TGATTTTATTTTGACTGATAGTGACCTGTTCGTTGCAACAAATTGATGAGCAATGCTTTTTTATAATGCCAACTTTGTACAAAAAAGCAGGCTTGGCTCGCCGACCGTATGACATGCTCTTCAAATTGCTGCTAATAGGCGACTCAGGAGTTGGAAAAAATTGCATTCTGTACAGATTTTCGGATGATGCCTTTAACACCACATTCATCTCAACTATTGGAATCGACTTCAAAATTAAAACTATCGAATTAAAAGGAAAAAAGATTAAATTACAAATCTGGGACACAGCTGGACAAGAACGATTCCACACAATCACAACATCATATTACCGCGGAGCAATGGGAATTATGCTGGTTTACGATATCACAAATGCGAAAAGTTTTGACAATATTGCAAAGTGGTTGCGCAATATTGACGAACACGCATCGGAAGATGTTGTTAAAATGATATTGGGAAATAAATGTGATATGAGTGATAGACGAGTTGTGAGCAGAGAACGAGGAGAGAAGATCGCACAAGATCATGGTATTAGCTTCCACGAGACTTCAGCTAAATTGAATGTTCACGTCGATACAGCGTTTTACGATTTAGCAGAAGCAATCCTGGCTAAGATGCCTGATTCCACTGACGAGCAATCCCGCGATACGGTGAATCCAGTGCAACCACAGAGGCAGAGTAGCAGTGGAGGATGCTGCCACCCAGCTTTCTTGTACAAAGTTGGCATTATAAGAAAGCATTGCTTATCAATTTGTTGCAACGAACAGGGTCACTATCAGTCAAAATAAAATCATTATTTGCCATCCAGCTGCAGCTCTGGCCCGTGTCTCAAAATCTCTGATGTTACATTGCACAAGATAAAAATATATCATCATGAACAATAAAACTGTCTGCTTACATAAACAGTAATA

>FlOCS_CN28-1_rab-10(CA)

AATGATTTTATTTTGACTGATAGTGACCTGTTCGTTGCAACAAATTGATGAGCAATGCTTTTTTATAATGCCAACTTTGTACAAAAAAGCAGGCTTGGCTCGCCGACCGTATGACATGCTCTTCAAATTGCTGCTAATAGGCGACTCAGGAGTTGGAAAAACTTGCATTCTGTACAGATTTTCGGATGATGCCTTTAACACCACATTCATCTCAACTATTGGAATCGACTTCAAAATTAAAACTATCGAATTAAAAGGAAAAAAGATTAAATTACAAATCTGGGACACAGCTGGACTCGAACGATTCCACACAATCACAACATCATATTACCGCGGAGCAATGGGAATTATGCTGGTTTACGATATCACAAATGCGAAAAGTTTTGACAATATTGCAAAGTGGTTGCGCAATATTGACGAACACGCATCGGAAGATGTTGTTAAAATGATATTGGGAAATAAATGTGATATGAGTGATAGACGAGTTGTGAGCAGAGAACGAGGAGAGAAGATCGCACAAGATCATGGTATTAGCTTCCACGAGACTTCAGCTAAATTGAATGTTCACGTCGATACAGCGTTTTACGATTTAGCAGAAGCAATCCTGGCTAAGATGCCTGATTCCACTGACGAGCAATCCCGCGATACGGTGAATCCAGTGCAACCACAGAGGCAGAGTAGCAGTGGAGGATGCTGCCACCCAGCTTTCTTGTACAAAGTTGGCATTATAAGAAAGCATTGCTTATCAATTTGTTGCAACGAACAGGTCACTATCAGTCAAAATAAAATCATTATTTGCCATCCAGCTGCAGCTCTGGCCCGTGTCTCAAAATCTCTGATGTTACATTGCACAAGATAAAAATATATCATCATGAACAATAAAACTGTCTGCTTACATAAACAGTAATA

>FlOCS_ZY2-2_rab-11.1(WT)

GATTTTATTTTGACTGATAGTGACCTGTTCGTTGCAACAAATTGATGAGCAATGCTTTTTTATAATGCCAACTTTGTACAAAAAAGCAGGCTTGGGCTCTCGTGACGATGAATACGACTACTTGTTCAAGGTTGTTCTGATTGGAGACTCAGGCGTCGGAAAGTCGAATCTCCTGTCTCGTTTCACAAGAAATGAGTTCAACTTGGAATCAAAATCAACAATCGGAGTCGAGTTTGCCACGAGAAGCATCTCGGTAGAAGGCAAGACAGTGAAGGCTCAAATTTGGGATACTGCTGGACAGGAACGTTACCGTGCCATCACATCCGCTTACTATCGTGGGGCTGTCGGAGCTCTCCTAGTCTACGACATCGCTAAGCATGTGACGTACGAGAATGTTGAGCGATGGTTGAAGGAGCTTCGTGATCACGCCGATCAGAACATTGTGATTATGTTGGTCGGAAACAAGAGCGACTTGCGCCATTTGCGTGCAGTTCCAACAGACGAGGCCAAGATCTACGCCGAAAGAAATCAATTGTCGTTTATTGAAACATCTGCCCTCGACAGCACCAACGTTGAAGCAGCTTTCACTAATATCCTGACGGAAATCTACAAATCAGTATCCAACAAGCATGTAGGAACTGACAGACAAGGATATGGCGGTGGCAGTGGTACAATCATTCCTTCGCCAGCGTCCGACCCACCAAAGAAGCAGTGTTGCATCCCACACCCAGCTTTCTTGTACAAAGTTGGCATTATAAGAAAGCATTGCTTATCAATTTGTTGCAACGAACAGGTCACTATCAGTCAAAATAAAATCATTATTTGCCATCCAGCTGCAGCTCTGGCCCGTGTCTCAAAATCTCTGATGTTACATTGCACAAGATAAAAATATATCATCATGAACAATAAAACTGTCTGCTTTACATAAACAGTAATAC

>FlOCS_PRI8-7_rab-11.1(DN)

TATGATTTTATTTTGACTGATAGTGACCTGTTCGTTGCAACAAATTGATGAGCAATGCTTTTTTATAATGCCAACTTTGTACAAAAAAGCAGGCTTGGGCTCTCGTGACGATGAATACGACTACTTGTTCAAGGTTGTTCTGATTGGAGACTCAGGCGTCGGAAAGAATAATCTCCTGTCTCGTTTCACAAGAAATGAGTTCAACTTGGAATCAAAATCAACAATCGGAGTCGAGTTTGCCACGAGAAGCATCTCGGTAGAAGGCAAGACAGTGAAGGCTCAAATTTGGGATACTGCTGGACAGGAACGTTACCGTGCCATCACATCCGCTTACTATCGTGGGGCTGTCGGAGCTCTCCTAGTCTACGACATCGCTAAGCATGTGACGTACGAGAATGTTGAGCGATGGTTGAAGGAGCTTCGTGATCACGCCGATCAGAACATTGTGATTATGTTGGTCGGAAACAAGAGCGACTTGCGCCATTTGCGTGCAGTTCCAACAGACGAGGCCAAGATCTACGCCGAAAGAAATCAATTGTCGTTTATTGAAACATCTGCCCTCGACAGCACCAACGTTGAAGCAGCTTTCACTAATATCCTGACGGAAATCTACAAATCAGTATCCAACAAGCATGTAGGAACTGACAGACAAGGATATGGCGGTGGCAGTGGTACAATCATTCCTTCGCCAGCGTCCGACCCACCAAAGAAGCAGTGTTGCATCCCACACCCAGCTTTCTTGTACAAAGTTGGCATTATAAGAAAGCATTGCTTATCAATTTGTTGCAACGAACAGGTCACTATCAGTCAAAATAAAATCATTATTTGCCATCCAGCTGCAGCTCTGGCCCGTGTCTCAAAATCTCTGATGTTACATTGCACAAGATAAAAATATATCATCATGAACAATAAAACTGTCTGCTTACATAA

>FlOCS_ZY10-1_rab-11.1(CA)

TTCATGGATGATATATTTTTATCTTGGTGCAATGTAACATCAGAGATTTTGAGACACGGGCCAGAGCTGCAGCTGGATGGCAAATAATGATTTTATTTTGACTGATAGTGACCTGTTCGTTGCAACAAATTGATAAGCAATGCTTTCTTATAATGCCAACTTTGTACAAGAAAGCTGGGTGTGGGATGCAACACTGCTTCTTTGGTGGGTCGGACGCTGGCGAAGGAATGATTGTACCACTGCCACCGCCATATCCTTGTCTGTCAGTTCCTACATGCTTGTTGGATACTGATTTGTAGATTTCCGTCAGGATATTAGTGAAAGCTGCTTCAACGTTGGTGCTGTCGAGGGCAGATGTTTCAATAAACGACAATTGATTTCTTTCGGCGTAGATCTTGGCCTCGTCTGTTGGAACTGCACGCAAATGGCGCAAGTCGCTCTTGTTTCCGACCAACATAATCACAATGTTCTGATCGGCGTGATCACGAAGCTCCTTCAACCATCGCTCAACATTCTCGTACGTCACATGCTTAGCGATGTCGTAGACTAGGAGAGCTCCGACAGCCCCACGATAGTAAGCGGATGTGATGGCACGGTAACGTTCCAGTCCAGCAGTATCCCAAATTTGAGCCTTCACTGTCTTGCCTTCTACCGAGATGCTTCTCGTGGCAAACTCGACTCCGATTGTTGATTTTGATTCCAAGTTGAACTCATTTCTTGTGAAACGAGACAGGAGATTCGACTTTCCGACGCCTGAGTCTCCAATCAGAACAACCTTGAACAAGTAGTCGTATTCATCGTCACGAGAGCCCAAGCCTGCTTTTTTGTACAAAGTTGGCATTATAAAAAAGCATGGCTCATCAATTTGTTGCAACGAACAGGTCACTATCAGTCAAATAAATCATTATTTGGGGGCCCGAAATCCATGCTA

>FlOCS_SP6-1_rab-14(WT)

ATGATTTTATTTTGACTGATAGTGACCTGTTCGTTGCAACAAATTGATGAGCAATGCTTTTTTATAATGCCAACTTTGTACAAAAAAGCAGGCTTGACGGCTGCTCCTTACAATTATTCGTATATTTTCAAATATATTATTATTGGTGATATGGGTGTCGGAAAATCGTGTCTTCTTCATCAGTTCACAGAAAAGAAGTTCATGGCCGACTGCCCGCACACAATCGGCGTGGAATTCGGCACCAGGATTATTGAGGTCAGCGGACAGAAGATCAAGCTTCAAATCTGGGACACGGCGGGCCAGGAGCGATTCCGCGCTGTGACGCGATCATATTATCGTGGAGCAGCCGGAGCCTTGATGGTCTACGACATCACTCGCCGTTCTACCTACAATCATTTGAGCAGCTGGTTGGCCGACGCAAAAAGCTTGACCAATCCAAATACGGCCATCTTTCTTATCGGAAACAAAGCCGACCTCGAAGATCAACGTGACGTGCCATACGAGGAAGCAAAAGCTTTCGCCGAAGAAAACGGGCTCACTTTCTTGGAGTGCAGTGCGAAGACTGGCAGTAACGTCGAAGACGCTTTCCTCGAAACGGCCAAGCAAATCTATCAGAATATCCAAGACGGAAGTTTGGACTTGAACGCCGCCGACACTGGCGTCCAGCCAAAACAGAACTTGCCACGTGCAGCTGAAAATAATGGGAAGAAGGACTGCAACTGCTACCCAGCTTTCTTGTACAAAGTTGGCATTATAAGAAAGCATTGCTTATCAATTTGTTGCAACGAACAGGTCACTATCAGTCAAAATAAAATCATTATTTGCCATCCAGCTGCAGCTCTGGCCCGTGTCTCAAAATCTCTGATGTTTACATTGCACAAGATAAAAATATATCATCATGAAC

>FlOCS_MHB43-1_rab-14(DN)

GATTTTATTTTGACTGATAGTGACCTGTTCGTTGCAACAAATTGATGAGCAATGCTTTTTTATAATGCCAACTTTGTACAAAAAAGCAGGCTTGACGGCTGCTCCTTACAATTATTCGTATATTTTCAAATATATTATTATTGGTGATATGGGTGTCGGAAAAAACTGTCTTCTTCATCAGTTCACAGAAAAGAAGTTCATGGCCGACTGCCCGCACACAATCGGCGTGGAATTCGGCACCAGGATTATTGAGGTCAGCGGACAGAAGATCAAGCTTCAAATCTGGGACACGGCGGGCCAGGAGCGATTCCGCGCTGTGACGCGATCATATTATCGTGGAGCAGCCGGAGCCTTGATGGTCTACGACATCACTCGCCGTTCTACCTACAATCATTTGAGCAGCTGGTTGGCCGACGCAAAAAGCTTGACCAATCCAAATACGGCCATCTTTCTTATCGGAAACAAAGCCGACCTCGAAGATCAACGTGACGTGCCATACGAGGAAGCAAAAGCTTTCGCCGAAGAAAACGGGCTCACTTTCTTGGAGTGCAGTGCGAAGACTGGCAGTAACGTCGAAGACGCTTTCCTCGAAACGGCCAAGCAAATCTATCAGAATATCCAAGACGGAAGTTTGGACTTGAACGCCGCCGACACTGGCGTCCAGCCAAAACAGAACTTGCCACGTGCAGCTGAAAATAATGGGAAGAAGGACTGCAACTGCTACCCAGCTTTCTTGTACAAAGTTGGCATTATAAGAAAGCATTGCTTATCAATTTGTTGCAACGAACAGGTCACTATCAGTCAAAATAAAATCATTATTTGCCATCCAGCTGCAGCTCTGGCCCGTGTCTCAAAATCTCTGATG

>FlOCS_SP51-1_rab-14(CA)

GATTTTATTTTGACTGATAGTGACCTGTTCGTTGCAACAAATTGATGAGCAATGCTTTTTTATAATGCCAACTTTGTACAAAAAAGCAGGCTTGACGGCTGCTCCTTACAATTATTCGTATATTTTCAAATATATTATTATTGGTGATATGGGTGTCGGAAAATCGTGTCTTCTTCATCAGTTCACAGAAAAGAAGTTCATGGCCGACTGCCCGCACACAATCGGCGTGGAATTCGGCACCAGGATTATTGAGGTCAGCGGACAGAAGATCAAGCTTCAAATCTGGGACACGGCGGGCCTCGAGCGATTCCGCGCTGTGACGCGATCATATTATCGTGGAGCAGCCGGAGCCTTGATGGTCTACGACATCACTCGCCGTTCTACCTACAATCATTTGAGCAGCTGGTTGGCCGACGCAAAAAGCTTGACCAATCCAAATACGGCCATCTTTCTTATCGGAAACAAAGCCGACCTCGAAGATCAACGTGACGTGCCATACGAGGAAGCAAAAGCTTTCGCCGAAGAAAACGGGCTCACTTTCTTGGAGTGCAGTGCGAAGACTGGCAGTAACGTCGAAGACGCTTTCCTCGAAACGGCCAAGCAAATCTATCAGAATATCCAAGACGGAAGTTTGGACTTGAACGCCGCCGACACTGGCGTCCAGCCAAAACAGAACTTGCCACGTGCAGCTGAAAATAATGGGAAGAAGGACTGCAACTGCTACCCAGCTTTCTTGTACAAAGTTGGCATTATAAGAAAGCATTGCTTATCAATTTGTTGCAACGAACAGGTCACTATCAGTCAAAATAAAATCATTATTTGCCATCCAGCTGCAGCTCTGGCCCGTGTCTCAAAATCTCTGATGTTACATTGCACAAGATAAAAATATATCATCATGAACAATAAAACTGTCTGCTTACATAAACAG

>FlOCS_NM7-2_rab-19(wt)

CGGGCCCAATAATGATTTTATTTTGACTGATAGTGACCTGTTCGTTGCAACAAATTGATGAGCAATGCTTTTTTATAATGCCAACTTTGTACAAAAAAGCAGGCTTGGACAACGATGATGGATTTGACTATTTGTTCAAAATTGTGCTTGTCGGCGATATGGGAGTCGGAAAGACATGTGTAGTTCAACGCTTCAGAAATGGAACATTTGTTGATCGTCAGGGAACCACTATCGGCGTCGATTTCACAATGAAAACTCTTGTCGTTGACGGAAAGCGTGTAAAATTGCAAATCTGGGATACTGGAGGCCAGGAACGATTCCGAACGATTACTCAATCATATTATCGATCTGCTAATGGAATTGTTTTGTGTTATGACATTACTTGCAAGCAATCATTTGGAAGTCTTCAGAGATGGATTGATGATGTTTCAAAGTTTGCAGCTCCGAATGTTGTGAAGCTACTCATTGGTACAAAATGCGATCTAGAAGACCAGAGAGCAATCGAAGCAGAAGAAGCGGAAATGTTGCAAAGAGCTAATGGAATGTTCGCAATGCTCGAGACTAGTGCAAAAGGCAATGTGAACGTGGACAACGCATTCCTCGAGTTAGCAACAATTCTGAAGCGACAGTATGATCAAGGAGTCGTTGAACAAGGCTCAAGTGGTACATTCCAGCTTGGATCCGGTGGCACGACGGCTCTGGGCTCTCCATGGCAACGATGTTGTCAGTACACTTACCCAGCTTTCTTGTACAAAGTTGGCATTATAAGAAAGCATTGCTTATCAATTTGTTGCAACGAACAGGTCACTATCAGTCAAAATAAAATCATTATTTGCCATCCAGCTGCAGCTCTGGCCCGTGTCTCAAAATCTCTGATGTTACATTGCACAAGATAAAAATATATCATCATGAACAATAAAACTGTCTGCTTACATAAAC

>FlOCS_KM29-1_rab-19(DN)

GATTTTATTTTGACTGATAGTGACCTGTTCGTTGCAACAAATTGATGAGCAATGCTTTTTTATAATGCCAACTTTGTACAAAAAAGCAGGCTTGGACAACGATGATGGATTTGACTATTTGTTCAAAATTGTGCTTGTCGGCGATATGGGAGTCGGAAAGAACTGTGTAGTTCAACGCTTCAGAAATGGAACATTTGTTGATCGTCAGGGAACCACTATCGGCGTCGATTTCACAATGAAAACTCTTGTCGTTGACGGAAAGCGTGTAAAATTGCAAATCTGGGATACTGGAGGCCAGGAACGATTCCGAACGATTACTCAATCATATTATCGATCTGCTAATGGAATTGTTTTGTGTTATGACATTACTTGCAAGCAATCATTTGGAAGTCTTCAGAGATGGATTGATGATGTTTCAAAGTTTGCAGCTCCGAATGTTGTGAAGCTACTCATTGGTACAAAATGCGATCTAGAAGACCAGAGAGCAATCGAAGCAGAAGAAGCGGAAATGTTGCAAAGAGCTAATGGAATGTTCGCAATGCTCGAGACTAGTGCAAAAGGCAATGTGAACGTGGACAACGCATTCCTCGAGTTAGCAACAATTCTGAAGCGACAGTATGATCAAGGAGTCGTTGAACAAGGCTCAAGTGGTACATTCCAGCTTGGATCCGGTGGCACGACGGCTCTGGGCTCTCCATGGCAACGATGTTGTCAGTACACTTACCCAGCTTTCTTGTACAAAGTTGGCATTATAAGAAAGCATTGCTTATCAATTTGTTGCAACGAACAGGTCACTATCAGTCAAAATAAAATCATTATTTGCCATCCAGCTGCAGCTCTGGCCCGTGTCTCAAAATCTCTGATGTTACATTGCACAAGATAAAAATATATCATCATGAACAATAAAACTGTCTGCTTACATAAACAGTAATACA

>FlOCS_DJM14-1_rab-19(CA)

GTACGAGGTTTATTGTTCATGATGATATATTTTTATCTTGTGCAATGTAACATCAGAGATTTTGAGACACGGGCCAGAGCTGCAGCTGGATGGCAAATAATGATTTTATTTTGACTGATAGTGACCTGTTCGTTGCAACAAATTGATAAGCAATGCTTTCTTATAATGCCAACTTTGTACAAGAAAGCTGGGTAAGTGTACTGACAACATCGTTGCCATGGAGAGCCCAGAGCCGTCGTGCCACCGGATCCAAGCTGGAATGTACCACTTGAGCCTTGTTCAACGACTCCTTGATCATACTGTCGCTTCAGAATTGTTGCTAACTCGAGGAATGCGTTGTCCACGTTCACATTGCCTTTTGCACTAGTCTCGAGCATTGCGAACATTCCATTAGCTCTTTGCAACATTTCCGCTTCTTCTGCTTCGATTGCTCTCTGGTCTTCTAGATCGCATTTTGTACCAATGAGTAGCTTCACAACATTCGGAGCTGCAAACTTTGAAACATCATCAATCCATCTCTGAAGACTTCCAAATGATTGCTTGCAAGTAATGTCATAACACAAAACAATTCCATTAGCAGATCGATAATATGATTGAGTAATCGTTCGGAATCGTTCAAGGCCTCCAGTATCCCAGATTTGCAATTTTACACGCTTTCCGTCAACGACAAGAGTTTTCATTGTGAAATCGACGCCGATAGTGGTTCCCTGACGATCAACAAATGTTCCATTTCTGAAGCGTTGAACTACACATGTCTTTCCGACTCCCATATCGCCGACAAGCACAATTTTGAACAAATAGTCAAATCCATCATCGTTGTCCAAGCCTGCTTTTTTGTACAAAGTTGGCATTATAAAAAAGCATTGCTCATCAATTTGTTGCAACGAACAGGTCACTATCAGTCAAAATAAAATCATTATTTGGGGCCCGAAATCCATGCTAGCGTTA

>FlOCS_LAK5-1_rab-21(WT)

CGGGCCAATAATGATTTTATTTTGACTGATAGTGACCTGTTCGTTGCAACAAATTGATGAGCAATGCTTTTTTATAATGCCAACTTTGTACAAAAAAGCAGGCTTGCTCGAAACCAACGTGGATCAGAAGTCGTTCAAATTCAAGATCGTGTTGCTCGGCGAAGGATGTGTTGGTAAATCATCACTTGTGCTTCGATTCGTTGAAAATAAATTCAGCTGCAAACACCTGTCGACGATTCAGGCAAGCTTCCAAAACAAAACTGTGAATGTAGAAGACTGCCAAGCTGACCTACACATCTGGGATACCGCTGGACAGGAGAAATACCACGCACTCGGTCCAATCTACTATCGAGGAAGCAATGGCGTGCTACTGGTTTTCGATATCACTGATAGAAAGTCATTTGAAAAGGTTAAGAACTGGGTTCTCGAAATAAAAACATGCCTGGGAAATACTGCAGAGATTCTGATAGTCGGGAACAAGATTGATCTTGAAGAAGAACGGCAGGTCACTCGACAGGATGCGGAAGCATACGCAGAATCAGAAGGGGCATTGTATATGGAGACTTCTGCTCAGGACAATGTTGGAATCTCTGATGCTTTTGAATCTTTAACTGCGAAAATGATCGAACATTCGCGTACTCGTTCGACAGAACCACCATCTACGAATCGATCCATCCGATTAATTGACAACGACGAAGCAGAAAGGAGTAAAAAGTGCTGTCGCTACCCAGCTTTCTTGTACAAAGTTGGCATTATAAGAAAGCATTGCTTATCAATTTGTTGCAACGAACAGGTCACTATCAGTCAAAATAAAATCATTATTTGCCATCCAGCTGCAGCTCTGGCCCGTGTCTCAAAATCTCTGATGTTACATTGCACAAGATAAAAATATATCATCATGAACAATAAAACTGTCTGCTTACATAACCAGTAATACAAGGGGTGTTATGAGCCATATTCCAACGGG

>FlOCS_JS31-4_rab-21(DN)

GATTTTATTTTGACTGATAGTGACCTGTTCGTTGCAACAAATTGATGAGCAATGCTTTTTTATAATGCCAACTTTGTACAAAAAAGCAGGCTTGCTCGAAACCAACGTGGATCAGAAGTCGTTCAAATTCAAGATCGTGTTGCTCGGCGAAGGATGTGTTGGTAAGAATTCACTTGTGCTTCGATTCGTTGAAAATAAATTCAGCTGCAAACACCTGTCGACGATTCAGGCAAGCTTCCAAAACAAAACTGTGAATGTAGAAGACTGCCAAGCTGACCTACACATCTGGGATACCGCTGGACAGGAGAAATACCACGCACTCGGTCCAATCTACTATCGAGGAAGCAATGGCGTGCTACTGGTTTTCGATATCACTGATAGAAAGTCATTTGAAAAGGTTAAGAACTGGGTTCTCGAAATAAAAACATGCCTGGGAAATACTGCAGAGATTCTGATAGTCGGGAACAAGATTGATCTTGAAGAAGAACGGCAGGTCACTCGACAGGATGCGGAAGCATACGCAGAATCAGAAGGGGCATTGTATATGGAGACTTCTGCTCAGGACAATGTTGGAATCTCTGATGCTTTTGAATCTTTAACTGCGAAAATGATCGAACATTCGCGTACTCGTTCGACAGAACCACCATCTACGAATCGATCCATCCGATTAATTGACAACGACGAAGCAGAAAGGAGTAAAAAGTGCTGTCGCTACCCAGCTTTCTTGTACAAAGTTGGCATTATAAGAAAGCATTGCTTATCAATTTGTTGCAACGAACAGGTCACTATCAGTCAAAATAAAATCATTATTTGCCATCCAGCTGCAGCTCTGGCCCGTGTCTCAAAATCTCTGATGTTACATTGCACAAGATAAAAATATATCATCATGAACAATAAAACTGTCTGCTTACATAAACAGTAATACA

>FlOCS_DS25-1_rab-21(CA)

ATATGATTTTATTTTGACTGATAGTGACCTGTTCGTTGCAACAAATTGATGAGCAATGCTTTTTTATAATGCCAACTTTGTACAAAAAAGCAGGCTTGCTCGAAACCAACGTGGATCAGAAGTCGTTCAAATTCAAGATCGTGTTGCTCGGCGAAGGATGTGTTGGTAAATCATCACTTGTGCTTCGATTCGTTGAAAATAAATTCAGCTGCAAACACCTGTCGACGATTCAGGCAAGCTTCCAAAACAAAACTGTGAATGTAGAAGACTGCCAAGCTGACCTACACATCTGGGATACCGCTGGACTCGAGAAATACCACGCACTCGGTCCAATCTACTATCGAGGAAGCAATGGCGTGCTACTGGTTTTCGATATCACTGATAGAAAGTCATTTGAAAAGGTTAAGAACTGGGTTCTCGAAATAAAAACATGCCTGGGAAATACTGCAGAGATTCTGATAGTCGGGAACAAGATTGATCTTGAAGAAGAACGGCAGGTCACTCGACAGGATGCGGAAGCATACGCAGAATCAGAAGGGGCATTGTATATGGAGACTTCTGCTCAGGACAATGTTGGAATCTCTGATGCTTTTGAATCTTTAACTGCGAAAATGATCGAACATTCGCGTACTCGTTCGACAGAACCACCATCTACGAATCGATCCATCCGATTAATTGACAACGACGAAGCAGAAAGGAGTAAAAAGTGCTGTCGCTACCCAGCTTTCTTGTACAAAGTTGGCATTATAAGAAAGCATTGCTTATCAATTTGTTGCAACGAACAGGTCACTATCAGTCAAAATAAAATCATTATTTGCCATCCAGCTGCAGCTCTGGCCCGTGTCTCAAAATCTCTGATGTTACATTGCACAAGATAAAAATATATCATCATGAACAATAAAACTGTCTGCTTACATAAACAGTAATACAA

>FlOCS_JP6-1_rab-27(WT)

CGGGGCCAATAATGATTTTATTTTGACTGATAGTGACCTGGTTCGTTGCAACAAATTGATGAGCAATGCTTTTTTATAATGCCAACTTTGTACAAAAAAGCAGGCTTGGGTGACTACGACTATCTCATCAAATTTCTCGCGCTCGGCGATTCGGGAGTCGGAAAAACGTCGTTTTTGCATCGTTACACGGATAACACGTTCACCGGACAATTCATTTCCACTGTTGGAATTGATTTTAAAGAGAAAAAAGTGGTCTACAAGAGTTCACGTGGCGGATTCGGTGGTCGTGGTCAACGAGTTTTACTTCAGTTATGGGACACTGCCGGACAAGAGAGATTCCGTTCTCTAACAACAGCGTTCTTCCGAGATGCAATGGGATTTATATTGATCTTCGATATTACAAATGAGCAATCATTTCTCAATATTCGAGACTGGCTATCACAATTGAAGGTACATGCCTATTGCGAGCAACCTGACATTATCATATGCGGTAACAAAGCAGACCTCGAAAATCGGCGTCAAGTCAGTACCGCCCGTGCAAAGCAGCTGGCTGATCAATTGGGGCTGCCGTACTTCGAGACGTCCGCCTGCACATCTACCAATGTGGAGAAATCTGTGGATTGTCTACTCGATTTGGTTATGCAAAGAATTCAACAATCTGTTGAGACGTCGTCTCTTCCGTTGTCAGAATGCCGCGGAGTCAGCTTGGACGGAGACCCATCGGCCGCTTCTTCCTATTGTGCAAATTGCTACCCAGCTTTCTTGTACAAAGTTGGCATTATAAGAAAGCATTGCTTATCAATTTGTTGCAACGAACAGGTCACTATCAGTCAAAATAAAATCATTATTTGCCATCCAGCTGCAGCTCTGGCCCGTGTCTCAAAATCTCTGATGTTACATTGCACAAGATAAAAATATATCATCATGAAC

>FlOCS_JP38-1_rab-27(DN)

GATTTTATTTTGACTGATAGTGACCTGTTCGTTGCAACAAATTGATGAGCAATGCTTTTTTATAATGCCAACTTTGTACAAAAAAGCAGGCTTGGGTGACTACGACTATCTCATCAAATTTCTCGCGCTCGGCGATTCGGGAGTCGGAAAAAACTCGTTTTTGCATCGTTACACGGATAACACGTTCACCGGACAATTCATTTCCACTGTTGGAATTGATTTTAAAGAGAAAAAAGTGGTCTACAAGAGTTCACGTGGCGGATTCGGTGGTCGTGGTCAACGAGTTTTACTTCAGTTATGGGACACTGCCGGACAAGAGAGATTCCGTTCTCTAACAACAGCGTTCTTCCGAGATGCAATGGGATTTATATTGATCTTCGATATTACAAATGAGCAATCATTTCTCAATATTCGAGACTGGCTATCACAATTGAAGGTACATGCCTATTGCGAGCAACCTGACATTATCATATGCGGTAACAAAGCAGACCTCGAAAATCGGCGTCAAGTCAGTACCGCCCGTGCAAAGCAGCTGGCTGATCAATTGGGGCTGCCGTACTTCGAGACGTCCGCCTGCACATCTACCAATGTGGAGAAATCTGTGGATTGTCTACTCGATTTGGTTATGCAAAGAATTCAACAATCTGTTGAGACGTCGTCTCTTCCGTTGTCAGAATGCCGCGGAGTCAGCTTGGACGGAGACCCATCGGCCGCTTCTTCCTATTGTGCAAATTGCTACCCAGCTTTCTTGTACAAAGTTGGCATTATAAGAAAGCATTGCTTATCAATTTGTTGCAACGAACAGGTCACTATCAGTCAAAATAAAATCATTATTTGCCATCCAGCTGCAGCTCTGGCCCGTGTCTCAAAATCTCTGATG

>FlOCS_SAN46-1_rab-27(CA)

GATTTTATTTTGACTGATAGTGACCTGTTCGTTGCAACAAATTGATGAGCAATGCTTTTTTATAATGCCAACTTTGTACAAAAAAGCAGGCTTGGGTGACTACGACTATCTCATCAAATTTCTCGCGCTCGGCGATTCGGGAGTCGGAAAAACGTCGTTTTTGCATCGTTACACGGATAACACGTTCACCGGACAATTCATTTCCACTGTTGGAATTGATTTTAAAGAGAAAAAAGTGGTCTACAAGAGTTCACGTGGCGGATTCGGTGGTCGTGGTCAACGAGTTTTACTTCAGTTATGGGACACTGCCGGTCTAGAGAGATTCCGTTCTCTAACAACAGCGTTCTTCCGAGATGCAATGGGATTTATATTGATCTTCGATATTACAAATGAGCAATCATTTCTCAATATTCGAGACTGGCTATCACAATTGAAGGTACATGCCTATTGCGAGCAACCTGACATTATCATATGCGGTAACAAAGCAGACCTCGAAAATCGGCGTCAAGTCAGTACCGCCCGTGCAAAGCAGCTGGCTGATCAATTGGGGCTGCCGTACTTCGAGACGTCCGCCTGCACATCTACCAATGTGGAGAAATCTGTGGATTGTCTACTCGATTTGGTTATGCAAAGAATTCAACAATCTGTTGAGACGTCGTCTCTTCCGTTGTCAGAATGCCGCGGAGTCAGCTTGGACGGAGACCCATCGGCCGCTTCTTCCTATTGTGCAAATTGCTACCCAGCTTTCTTGTACAAAGTTGGCATTATAAGAAAGCATTGCTTATCAATTTGTTGCAACGAACAGGTCACTATCAGTCAAAATAAAATCATTATTTGCCATCCAGCTGCAGCTCTGGCCCGTGTCTCAAAATCTCTGATGTTACATTGCACAA

>FlOCS_SVP6-1_rab-28(WT)

CGGGCCCAATATGATTTTATTTTGACTGATAGTGACCTGTTCGTTGCAACAAATTGATGAGCAATGCTTTTTTATAATGCCAACTTTGTACAAAAAAGCAGGCTTGACAACAATGGGAGAAGACGAGGCTCCAGCTTTGCCAAAGAAATCACCATTACCTGAGAAAATTGACGAAGCTGATGTGGATGATGATCCGGATGATAAAGTTATCAAGATAGTGGTAGTCGGAGATGGAGCAAGTGGAAAGACATCAATTTGCCAGAGATTTGCAAAAGAATCTTTTGATAAATCTTATCACCAAACTCTTGGTTTGGATTTCTTCTCTCGTCGAATCACGCTTCCACACGAAATGCAGGTGCTCGTGCAGGTATGGGATATTGGAGGACAAAGTATTGCTGGTGAAATGATCGATAAGTACTTGACTGGAGCCAACATCGTTTTTCTCGTTTATGATGTCACCAACTCAAAATCCTTTGAAAATGCTGTCGATTGGTTGTCAGTTGTCAAGAAAAACACGAAATCCAGTGAAACACCGGTGAAACTAGTATTGATGGGAAATAAGACGGATCTTGAGGAGCGACGTGTGGTTTCAGTTGAAGCACACAAGAATTTTGCGACTTCCAACGACATGATGCCAACTTACGTTTCTGCAAAAACTGGAGATACTGTATTTCTCACATTCAGACAAGCCGTTGCTGAAGTACTCAATGTGGGTCTTTCGAGAGCTGAAGTTGAAGCTGACATCGAAATTGTTCAAGGATCTGTTATTGAACAGCCTAAGCAATCAGACGCCTCATATGCTCGCCGTTCGGATCAAAGTCGCAGTACATCTGTATGCTCCATTACTTACCCAGCTTTCTTGTACAAAGTTGGCATTATAAGAAAGCATTGCTTATCAATTTGTTGCAACGAACAGGTCACTATCAGTCAAAATAAAATCATTATTTGCCATCCA

>FlOCS_ST21-1_rab-28(DN)

GATTTTATTTTGACTGATAGTGACCTGTTCGTTGCAACAAATTGATGAGCAATGCTTTTTTATAATGCCAACTTTGTACAAAAAAGCAGGCTTGACAACAATGGGAGAAGACGAGGCTCCAGCTTTGCCAAAGAAATCACCATTACCTGAGAAAATTGACGAAGCTGATGTGGATGATGATCCGGATGATAAAGTTATCAAGATAGTGGTAGTCGGAGATGGAGCAAGTGGAAAGAATTCAATTTGCCAGAGATTTGCAAAAGAATCTTTTGATAAATCTTATCACCAAACTCTTGGTTTGGATTTCTTCTCTCGTCGAATCACGCTTCCACACGAAATGCAGGTGCTCGTGCAGGTATGGGATATTGGAGGACAAAGTATTGCTGGTGAAATGATCGATAAGTACTTGACTGGAGCCAACATCGTTTTTCTCGTTTATGATGTCACCAACTCAAAATCCTTTGAAAATGCTGTCGATTGGTTGTCAGTTGTCAAGAAAAACACGAAATCCAGTGAAACACCGGTGAAACTAGTATTGATGGGAAATAAGACGGATCTTGAGGAGCGACGTGTGGTTTCAGTTGAAGCACACAAGAATTTTGCGACTTCCAACGACATGATGCCAACTTACGTTTCTGCAAAAACTGGAGATACTGTATTTCTCACATTCAGACAAGCCGTTGCTGAAGTACTCAATGTGGGTCTTTCGAGAGCTGAAGTTGAAGCTGACATCGAAATTGTTCAAGGATCTGTTATTGAACAGCCTAAGCAATCAGACGCCTCATATGCTCGCCGTTCGGATCAAAGTCGCAGTACATCTGTATGCTCCATTACTTACCCAGCTTTCTTGTACAAAGTTGGCATTATAAGAAAGCATTGCTTATCAATTTGTTGCAACGAACAGGTCACTATCAGTCAAAATAAAATCATTATTTGCCATCCAGCTGCAGCTCTGGCCCGTGTCTCAAAATCTCTGA

>FlOCS_SVP57-2_rab-28(CA)

ATAATGATTTTATTTTGACTGATAGTGACCTGTTCGTTGCAACAAATTGATGAGCAATGCTTTTTTATAATGCCAACTTTGTACAAAAAAGCAGGCTTGACAACAATGGGAGAAGACGAGGCTCCAGCTTTGCCAAAGAAATCACCATTACCTGAGAAAATTGACGAAGCTGATGTGGATGATGATCCGGATGATAAAGTTATCAAGATAGTGGTAGTCGGAGATGGAGCAAGTGGAAAGACATCAATTTGCCAGAGATTTGCAAAAGAATCTTTTGATAAATCTTATCACCAAACTCTTGGTTTGGATTTCTTCTCTCGTCGAATCACGCTTCCACACGAAATGCAGGTGCTCGTGCAGGTATGGGATATTGGAGGACTAAGTATTGCTGGTGAAATGATCGATAAGTACTTGACTGGAGCCAACATCGTTTTTCTCGTTTATGATGTCACCAACTCAAAATCCTTTGAAAATGCTGTCGATTGGTTGTCAGTTGTCAAGAAAAACACGAAATCCAGTGAAACACCGGTGAAACTAGTATTGATGGGAAATAAGACGGATCTTGAGGAGCGACGTGTGGTTTCAGTTGAAGCACACAAGAATTTTGCGACTTCCAACGACATGATGCCAACTTACGTTTCTGCAAAAACTGGAGATACTGTATTTCTCACATTCAGACAAGCCGTTGCTGAAGTACTCAATGTGGGTCTTTCGAGAGCTGAAGTTGAAGCTGACATCGAAATTGTTCAAGGATCTGTTATTGAACAGCCTAAGCAATCAGACGCCTCATATGCTCGCCGTTCGGATCAAAGTCGCAGTACATCTGTATGCTCCATTACTTACCCAGCTTTCTTGTACAAAGTTGGCATTATAAGAAAGCATTGCTTATCAATTTGTTGCAACGAACAGGTCACTATCAGTCAAAATAAAATCATTATTTGC

>FlOCS_MEL4-1_rab-30(WT)

ATGATTTTATTTTGACTGATAGTGACCTGTTCGTTGCAACAAATTGATGAGCAATGCTTTTTTATAATGCCAACTTTGTACAAAAAAGCAGGCTTGGAGGATTACAAGTATCTATTCAAAGTGGTACTTGTGGGAAATGCGGGCGTTGGGAAAACATGTCTAGTCAGAAAGTTCACACAGGGTATATTTCCTCCTGGTCAAAGTGCAACAATCGGCGTCGATTTTATGATAAAAACTGTTAAAGTAGGGAACGATAAAATTAAGCTTCAAATTTGGGATACGGCAGGTCAGGAGAGGTTTCGATCGATTACGCAGAGTTATTACAGAAGCGCCCACGCAATAGTTCTAGTGTACGATGTATCGTGTCAACCATCATTTGATTGTCTTCCAGAATGGCTTGGTGAAATCGAAAGTTATGCAAATCGGCGAGTTTTGAAAATTCTTGTTGGAAACAAAGTCGATAAGGGAGACGAACGGGAAGTTCCCGAACGAATTGGAAGAGATTTTTCTGATGTCAACCAATTCGACTATTTTCTTGAAACTTCGGCACTTGACGCGACAAATGTAGATCAATTGTTCGAACAAGTCGCCACAAGGTTAACAAATGATATGAAACTCACTGATGAACGGGTACACCAATTCCGAGCAGACGCGACGAACTCCTCTTCCAGCACGGGCGGTCCCATCAAATTAATCGATCGAGCGCAGACACAACTGAATTCCTGTTGTACTCGACAATCTTACCCAGCTTTCTTGTACAAAGTTGGCATTATAAGAAAGCATTGCTTATCAATTTGTTGCAACGAACAGGTCACTATCAGTCAAAATAAAATCATTATTTGCCATCCAGCTGCAGCTCTGGCCCGTGTCTCAAAATCTCTGAT

>FlOCS_TT18-1_rab-30(DN)

TTTGACTGATAGTGACCTGTTCGTTGCAACAAATTGATGAGCAATGCTTTTTTATAATGCCAACTTTGTACAAAAAAGCAGGCTTGGAGGATTACAAGTATCTATTCAAAGTGGTACTTGTGGGAAATGCGGGCGTTGGGAAAAACTGTCTAGTCAGAAAGTTCACACAGGGTATATTTCCTCCTGGTCAAAGTGCAACAATCGGCGTCGATTTTATGATAAAAACTGTTAAAGTAGGGAACGATAAAATTAAGCTTCAAATTTGGGATACGGCAGGTCAGGAGAGGTTTCGATCGATTACGCAGAGTTATTACAGAAGCGCCCACGCAATAGTTCTAGTGTACGATGTATCGTGTCAACCATCATTTGATTGTCTTCCAGAATGGCTTGGTGAAATCGAAAGTTATGCAAATCGGCGAGTTTTGAAAATTCTTGTTGGAAACAAAGTCGATAAGGGAGACGAACGGGAAGTTCCCGAACGAATTGGAAGAGATTTTTCTGATGTCAACCAATTCGACTATTTTCTTGAAACTTCGGCACTTGACGCGACAAATGTAGATCAATTGTTCGAACAAGTCGCCACAAGGTTAACAAATGATATGAAACTCACTGATGAACGGGTACACCAATTCCGAGCAGACGCGACGAACTCCTCTTCCAGCACGGGCGGTCCCATCAAATTAATCGATCGAGCGCAGACACAACTGAATTCCTGTTGTACTCGACAATCTTACCCAGCTTTCTTGTACAAAGTTGGCATTATAAGAAAGCATTGCTTATCAATTTGTTGCAACGAACAGGTCACTATCAGTCAAAATAAAATCATTATTTGCCATCCAGCTGCAGCTCTGGCCCGTGTCTCAAA

>FlOCS_SMJ11-1_rab-30(CA)

GATTTTATTTTGACTGATAGTGACCTGTTCGTTGCAACAAATTGATGAGCAATGCTTTTTTATAATGCCAACTTTGTACAAAAAAGCAGGCTTGGAGGATTACAAGTATCTATTCAAAGTGGTACTTGTGGGAAATGCGGGCGTTGGGAAAACATGTCTAGTCAGAAAGTTCACACAGGGTATATTTCCTCCTGGTCAAAGTGCAACAATCGGCGTCGATTTTATGATAAAAACTGTTAAAGTAGGGAACGATAAAATTAAGCTTCAAATTTGGGATACGGCAGGTCTCGAGAGGTTTCGATCGATTACGCAGAGTTATTACAGAAGCGCCCACGCAATAGTTCTAGTGTACGATGTATCGTGTCAACCATCATTTGATTGTCTTCCAGAATGGCTTGGTGAAATCGAAAGTTATGCAAATCGGCGAGTTTTGAAAATTCTTGTTGGAAACAAAGTCGATAAGGGAGACGAACGGGAAGTTCCCGAACGAATTGGAAGAGATTTTTCTGATGTCAACCAATTCGACTATTTTCTTGAAACTTCGGCACTTGACGCGACAAATGTAGATCAATTGTTCGAACAAGTCGCCACAAGGTTAACAAATGATATGAAACTCACTGATGAACGGGTACACCAATTCCGAGCAGACGCGACGAACTCCTCTTCCAGCACGGGCGGTCCCATCAAATTAATCGATCGAGCGCAGACACAACTGAATTCCTGTTGTACTCGACAATCTTACCCAGCTTTCTTGTACAAAGTTGGCATTATAAGAAAGCATTGCTTATCAATTTGTTGCAACGAACAGGTCACTATCAGTCAAAATAAAATCATTATTTGCCATCCAGCTGCAGCTCTGGCCCGTGTCTCAAAATCTCTGATGTTACATTGCACAAGATAAAAATATATCATCATGAACAATAAAACTGTCTGCTTACATAAACAG

>FlOCS_TR6-1_rab-33(WT)

TATGATTTTATTTTGACTGATAGTGACCTGTTCGTTGCAACAAATTGATGAGCAATGCTTTTTTATAATGCCAACTTTGTACAAAAAAGCAGGCTTGTCGGAGCATCATGTGAACATCCCGGCTCCTCAGTTCTCCACGTATGCTACTGTAATTGATCCACCGAAACACGTCACTGCAACTCATCCTGATGAGCTGACAACAGCATCACATCCCCAACCAACACATCCATCAGCACCACAAGATCCTTCACCAGCAGTACCATCAACTCCAGTTCGTGTTCCATATCCAACAGCTCCTCCACCAATACCTCCGGCTCCAGAAGCAGTGACAGCTGGGCCGAAGAAAATGGCGTTGGCTCCGTCCTCAACGAAAACATACAAACAGAAGAGAACTTTCAAGGTAATCATAGTGGGCAACGCGGCCGTCGGAAAAACGTGTCTTTCATTCCGTTTTTGTTGTGGAAGATTCCCCGAACATACTGAAGCCACAATCGGAGTGGATTTTCGAGAAAGAAGTTGTGTAATTGAAAATGAACTTCTTCGTGTTCAGCTATGGGATACTGCTGGACAGGAAAGATATCGACAATCGATTGTTGCTCATTATTATAGAAATGTGAATGCTGTCGTTTTTGTGTATGATGTCACATGCCGTGAGTCATTCAACGATTTGGCACTTTGGATAAAAGAATGTGAAAAGCATGGTCTCGTTGGGGATAGTGAAGTTCCGAGGATCCTGATCGGCAACAAGTGTGACGTGGAATGCACAAATCGAGTGTCCACTGATGAAGCTCAGATGTTCGCCGACCGTAACAACATGGCATTATTCGAAACATCAGCCAAATTAGCATCAGAAGCAGATCATGTCGAATCAATTTTTCTCACACTGTTACACAAACTACAACAAAGTAAACCAATGCACGTACAATCACAAGATGAACGACATCAAAAAGAGCAAGAACGGTTGATTTTGAAGGCAAATGAGACGGAAAATGTGGAAGAAGAGGGATTCTGCTGCTACCCAGCTTTCTTGTACAAAGTTGGCATTATAAGAAAGCATTGCTTATCAATTTGTTGCAACGAACAGGTCACTATCAGTCAAAATAAAATCATTATTTGCCATCCAGCTGCAGCTCTGGCCCGTGTCTCAAAATCTCTGATGTTACATTGCACAAGATAAAAATATATCATCATGAACAAT

>FlOCS_TR34-1_rab-33(DN)

ATATGATTTTATTTTGACTGATAGTGACCTGTTCGTTGCAACAAATTGATGAGCAATGCTTTTTTATAATGCCAACTTTGTACAAAAAAGCAGGCTTGTCGGAGCATCATGTGAACATCCCGGCTCCTCAGTTCTCCACGTATGCTACTGTAATTGATCCACCGAAACACGTCACTGCAACTCATCCTGATGAGCTGACAACAGCATCACATCCCCAACCAACACATCCATCAGCACCACAAGATCCTTCACCAGCAGTACCATCAACTCCAGTTCGTGTTCCATATCCAACAGCTCCTCCACCAATACCTCCGGCTCCAGAAGCAGTGACAGCTGGGCCGAAGAAAATGGCGTTGGCTCCGTCCTCAACGAAAACATACAAACAGAAGAGAACTTTCAAGGTAATCATAGTGGGCAACGCGGCCGTCGGAAAAAACTGTCTTTCATTCCGTTTTTGTTGTGGAAGATTCCCCGAACATACTGAAGCCACAATCGGAGTGGATTTTCGAGAAAGAAGTTGTGTAATTGAAAATGAACTTCTTCGTGTTCAGCTATGGGATACTGCTGGACAGGAAAGATATCGACAATCGATTGTTGCTCATTATTATAGAAATGTGAATGCTGTCGTTTTTGTGTATGATGTCACATGCCGTGAGTCATTCAACGATTTGGCACTTTGGATAAAAGAATGTGAAAAGCATGGTCTCGTTGGGGATAGTGAAGTTCCGAGGATCCTGATCGGCAACAAGTGTGACGTGGAATGCACAAATCGAGTGTCCACTGATGAAGCTCAGATGTTCGCCGACCGTAACAACATGGCATTATTCGAAACATCAGCCAAATTAGCATCAGAAGCAGATCATGTCGAATCAATTTTTCTCACACTGTTACACAAACTACAACAAAGTAAACCAATGCACGTACAATCACAAGATGAACGACATCAAAAAGAGCAAGAACGGTTGATTTTGAAGGCAAATGAGACGGAAAATGTGGAAGAAGAGGGATTCTGCTGCTACCCAGCTTTCTTGTACAAAGTTGGCATTATAAGAAAGCATTGCTTATCAATTTGTTGCAACGAACAGGTCACTATCAGTCAAAATAAAATCATTATTTGCCATCCAGCTGCAGCTCT

>FlOCS_MAH25-1_rab-33(CA)

GATTTTATTTTGACTGATAGTGACCTGTTCGTTGCAACAAATTGATGAGCAATGCTTTTTTATAATGCCAACTTTGTACAAAAAAGCAGGCTTGTCGGAGCATCATGTGAACATCCCGGCTCCTCAGTTCTCCACGTATGCTACTGTAATTGATCCACCGAAACACGTCACTGCAACTCATCCTGATGAGCTGACAACAGCATCACATCCCCAACCAACACATCCATCAGCACCACAAGATCCTTCACCAGCAGTACCATCAACTCCAGTTCGTGTTCCATATCCAACAGCTCCTCCACCAATACCTCCGGCTCCAGAAGCAGTGACAGCTGGGCCGAAGAAAATGGCGTTGGCTCCGTCCTCAACGAAAACATACAAACAGAAGAGAACTTTCAAGGTAATCATAGTGGGCAACGCGGCCGTCGGAAAAACGTGTCTTTCATTCCGTTTTTGTTGTGGAAGATTCCCCGAACATACTGAAGCCACAATCGGAGTGGATTTTCGAGAAAGAAGTTGTGTAATTGAAAATGAACTTCTTCGTGTTCAGCTATGGGATACTGCTGGACTGGAAAGATATCGACAATCGATTGTTGCTCATTATTATAGAAATGTGAATGCTGTCGTTTTTGTGTATGATGTCACATGCCGTGAGTCATTCAACGATTTGGCACTTTGGATAAAAGAATGTGAAAAGCATGGTCTCGTTGGGGATAGTGAAGTTCCGAGGATCCTGATCGGCAACAAGTGTGACGTGGAATGCACAAATCGAGTGTCCACTGATGAAGCTCAGATGTTCGCCGACCGTAACAACATGGCATTATTCGAAACATCAGCCAAATTAGCATCAGAAGCAGATCATGTCGAATCAATTTTTCTCACACTGTTACACAAACTACAACAAAGTAAACCAATGCACGTACAATCACAAGATGAACGACATCAAAAAGAGCAAGAACGGTTGATTTTGAAGGCAAATGAGACGGAAAATGTGGAAGAAGAGGGATTCTGCTGCTACCCAGCTTTCTTGTACAAAGTTGGCATTATAAGAAAGCATTGCTTATCAATTTGTTGCAACGAACAGGTCACTATCAGTCAAAATAAAATCATTATTTGCCATCCAGCTGCAGCTCTGGCCCGTGTCTCAAAATCTCTGATGTTACATTGCACAAGATAAAAATAT

>FlOCS_RV3-1_rab-37(WT)

AATAATGATTTTATTTTGACTGATAGTGACCTGTTCGTTGCAACAAATTGATGAGCAATGCTTTTTTATAATGCCAACTTTGTACAAAAAAGCAGGCTTGTTTTTAAAGGTTATGCTACTTGGTGACAGCTGCACTGGAAAAACGTGCCTTCTGATTCGATACAAAGATGGAGCTTTTCTAAATAACAATTTCATTTCAACAGTTGGAATTGATTACAGAAATAAACTAATCACAATGGGGGATAAGAAGGTCAAACTTCAGATTTGGGACACCGCGGGACAAGAACGTTTTCGATCAGTGACCACATCATACTACCGCGACGCCGATGCTCTTCTCTTGGTCTACGACATCGCCAATCGTGCAAGCTTTGAGAACTGCCGGAACTGGCTCTCTCAAATCAAAGAATATGGGAAAGAGGCGGTACAAGTGACACTTGTTGGCAACAAATGTGATTTGCCAAGAGCAGTACCGACGGATGAGGGAAAACGTCTTGCTGAGGCCTATCAAATTCCATTCATGGAGACGAGCGCCAAAACTGGGTTCAATGTAGATAGAGCATTCCTCGGATTGGCAGAACGAATGCTCAAGTTGAAATACGGTTTTGTTCCCGGAGGTGAAATGGCGGACACGATTTCCGTCGCCGACACCAAGAAGCCGGAAATCGCCAGATGTTGCACGTTTAATTACCCAGCTTTCTTGTACAAAGTTGGCATTATAAGAAAGCATTGCTTATCAATTTGTTGCAACGAACAGGTCACTATCAGTCAAAATAAAATCATTATTTGCCATCCAGCTGCAGCTCTGGCCCGTGTCTCAAAATCTCTGATGTTACATTGCACAAGATAAAAATATATCATCATGAACAATAAAACTGTCTGCTTACATAAACAGTAATACAAGGGGTGTTATGAGCCATATTCAACGGGAAACGTCGAGGCCGCGATTAAATTCCAACATGG

>FlOCS_ZW10-1_rab-37(DN)

GATGATATATTTTTATCTTGTGCAATGTAACATCAGAGATTTTGAGACACGGGCCAGAGCTGCAGCTGGATGGCAAATAATGATTTTATTTTGACTGATAGTGACCTGTTCGTTGCAACAAATTGATAAGCAATGCTTTCTTATAATGCCAACTTTGTACAAGAAAGCTGGGTAATTAAACGTGCAACATCTGGCGATTTCCGGCTTCTTGGTGTCGGCGACGGAAATCGTGTCCGCCATTTCACCTCCGGGAACAAAACCGTATTTCAACTTGAGCATTCGTTCTGCCAATCCGAGGAATGCTCTATCTACATTGAACCCAGTTTTGGCGCTCGTCTCCATGAATGGAATTTGATAGGCCTCAGCAAGACGTTTTCCCTCATCCGTCGGTACTGCTCTTGGCAAATCACATTTGTTGCCAACAAGTGTCACTTGTACCGCCTCTTTCCCATATTCTTTGATTTGAGAGAGCCAGTTCCGGCAGTTCTCAAAGCTTGCACGATTGGCGATGTCGTAGACCAAGAGAAGAGCATCGGCGTCGCGGTAGTATGATGTGGTCACTGATCGAAAACGTTCTTGTCCCGCGGTGTCCCAAATCTGAAGTTTGACCTTCTTATCCCCCATTGTGATTAGTTTATTTCTGTAATCAATTCCAACTGTTGAAATGAAATTGTTATTTAGAAAAGCTCCATCTTTGTATCGAATCAGAAGGCAGTTTTTTCCAGTGCAGCTGTCACCAAGTAGCATAACCTTTAAAAACAAGCCTGCTTTTTTGTACAAAGTTGGCATTATAAAAAAGCATTGCTCATCAATTTGTTGCAACGAACAGGTCACTATCAGTCAAAATAAAATCATTATTTTGGGGCCCGAGATCCATGCTAGCGTTAACGCGAGAGT

>FlOCS_KLD50-1_rab-37(CA)

ATAATGATTTTATTTTGACTGATAGTGACCTGTTCGTTGCAACAAATTGATGAGCAATGCTTTTTTATAATGCCAACTTTGTACAAAAAAGCAGGCTTGTTTTTAAAGGTTATGCTACTTGGTGACAGCTGCACTGGAAAAACGTGCCTTCTGATTCGATACAAAGATGGAGCTTTTCTAAATAACAATTTCATTTCAACAGTTGGAATTGATTACAGAAATAAACTAATCACAATGGGGGATAAGAAGGTCAAACTTCAGATTTGGGACACCGCGGGACTCGAACGTTTTCGATCAGTGACCACATCATACTACCGCGACGCCGATGCTCTTCTCTTGGTCTACGACATCGCCAATCGTGCAAGCTTTGAGAACTGCCGGAACTGGCTCTCTCAAATCAAAGAATATGGGAAAGAGGCGGTACAAGTGACACTTGTTGGCAACAAATGTGATTTGCCAAGAGCAGTACCGACGGATGAGGGAAAACGTCTTGCTGAGGCCTATCAAATTCCATTCATGGAGACGAGCGCCAAAACTGGGTTCAATGTAGATAGAGCATTCCTCGGATTGGCAGAACGAATGCTCAAGTTGAAATACGGTTTTGTTCCCGGAGGTGAAATGGCGGACACGATTTCCGTCGCCGACACCAAGAAGCCGGAAATCGCCAGATGTTGCACGTTTAATTACCCAGCTTTCTTGTACAAAGTTGGCATTATAAGAAAGCATTGCTTATCAATTTGTTGCAACGAACAGGTCACTATCAGTCAAAATAAAATCATTATTTGCCATCCAGCTGCAGCTCTGGCCCGTGTCTCAAAATCTCTGA

>FlOCS_DVD8-2_rabY1(WT)

ATATGATTTTATTTTGACTGATAGTGACCTGTTCGTTGCAACAAATTGATGAGCAATGCTTTTTTATAATGCCAACTTTGTACAAAAAAGCAGGCTTGTCGAAACCCGAGGTGGAGAATCTCTTTTCTCTCTGCGACTCCGAATCCAAGGGATACCTGACGATGGAGGATTTGAGGAAAGTGTGCCCACAGCTTGATGATAATGATTTGCGTTTCATATTCACGGAGTTGGATCAAGATGGTTCTGGAAAAATTGAGAAGTTGGAATTTTTGCGGGGTTTCCAAGACACTGTACAGCACGGAGAAAGTCATGGCCTGAATGGTATGCAGCGTCGAGCATCGGTTGCTGTAGAGGACCCACCAATGTTTCTACGTGATGAACAAATGTTTGACAGTGAAAGTGACTCGACTTCCTCTCGACCAGCGATCCGAGTATTTGACGAAGAACACTATCATTCAGAGTCCGATACAAATATTAACATTGACTTTAGCGTTCCATGCCAAGAAGAAGTATTGGTGCTGTACGAACAACTACAGTCATCTGGTGTTCCTGCATTGTTAAGGAAGTTTGAGAGAGTTGTTGGATCATTTCACAAAGAACTATCAGAGAAGAAGCATGAAAATGAGCGACTTCAACGTATTTATGCAAGTGAACGTGAAATGTACAATCGTAGAATGGAAGAGATGGAAAGCGAAGTTGACCAACAACTGGAGCTCACAGAAATGAAAGCCCGGCAAGAGGAGCGAGACCGTCTGACAAAAGAGAAAGAAGAAATGCGCCAAAGAATGTCGGATGAAATGTCAGAAATGAGAAACAACATAGAAAGACTGCAGAAAATGGAAAAAGCTCTTGAACGGGAAAATGAACGGCTGAACCATCAAAAAGAGTTGTCTGATAAACTGAAAGTTGTAAATGAGGAAAATAACGACTTGCGACAAAATCTTGCTGAAAACCATCTCGAACTGGCGATGATCAAGAGTGAGCTTGCACAAGTCAGATGTGAATTTGATCAGAAGCAGGATGAACTTTCTGCAAGAAGAGATCAAGCTTCCCACGCCACCGAGGAAAGTGAATCTGTCAGGAAACAATTGCAACTCCTATTTGATGCCAATCGCAAATTGCACGAAACCAATGAAAGCCTCCGCGATGCACTGGATAGCAGAGCATCTGTTCTCCGACAGTTCAATTTGAGAACTCCTTCTCCCGGATTGCTCAGCTCGAATCGTAATTCTGTGGAGAACTTTCAAACGTCCACGAACGTGTTCCGATCCGTGCCACTTCATGCGATTAGTACTGAGGAGCAAGTGCCAGAAACATCATTGATTCTAGACGATGCGCATAGCTTGCAAGGGCTCGATACTCCGGGAGATCTTATGGGCCTCAATGATGCTAACGGCCCAGCTGAAAGAACGTTCCGTATCGTGATGTGTGGAGACGCCGCTGTTGGAAAAAGTAGTTTTGTAATGCGGGTGATCCGTCGTCAATTCACAAATCAGCTCCCCAGCACACTTGGCGTAGATTTTCACGTGAAGACTGTCAATGTTGACGGAAGAAACGTTGCACTTCAGCTATGGGACACCGCGGGACAAGAACGTTTCCGATCACTTTGTAAATCATATTTTCGTCGAGCTGATGGAGCAATCCTAGTCTATGACGTCTGTGCGGAGCAGTCGTTTCTCCGTGTTCGCGACTGGATTGAGACTATAAAGGAGTCAACAGAAAGGAGTATTCCGATCATTTTGGTAGGAAACAAGGTTGATATGCGTATTTCGACGCCAGGCTCGGTGGCAAAGACAGATGGTGCAAGTATGGCTGCGGCGATGGGAGTTCTATTCATGGAAACCAGTGCTTTAGACGGAAGTAACATAGATAACGCTATGTTGGCACTAACCAGAGAGCTCATGGCAGTAGAAGATGTTGAAATTCGCTCAACAGGTGTTGTGCTCAATCCAGCAGTTGCAAAGAAGGGTGGATGTTTCTCAAAGTGTCGTGGTTCTTACCCAGCTTTCTTGTACAAAGTTGGCATTATAAGAAAGCATTGCTTATCAATTTGTTGCAACGAACAGGTCACTATCAGTCAAAATAAAATCATTATTTGCCATCCAGCTGCAGCTCTGGCCCGTGTCTCAAAATCTCTGATGTTACATTGCACAAGATAAAAATATATCATC

>FlOCS_DVD36-2_rabY1(DN)

GATTTTATTTTGACTGATAGTGACCTGTTCGTTGCAACAAATTGATGAGCAATGCTTTTTTATAATGCCAACTTTGTACAAAAAAGCAGGCTTGTCGAAACCCGAGGTGGAGAATCTCTTTTCTCTCTGCGACTCCGAATCCAAGGGATACCTGACGATGGAGGATTTGAGGAAAGTGTGCCCACAGCTTGATGATAATGATTTGCGTTTCATATTCACGGAGTTGGATCAAGATGGTTCTGGAAAAATTGAGAAGTTGGAATTTTTGCGGGGTTTCCAAGACACTGTACAGCACGGAGAAAGTCATGGCCTGAATGGTATGCAGCGTCGAGCATCGGTTGCTGTAGAGGACCCACCAATGTTTCTACGTGATGAACAAATGTTTGACAGTGAAAGTGACTCGACTTCCTCTCGACCAGCGATCCGAGTATTTGACGAAGAACACTATCATTCAGAGTCCGATACAAATATTAACATTGACTTTAGCGTTCCATGCCAAGAAGAAGTATTGGTGCTGTACGAACAACTACAGTCATCTGGTGTTCCTGCATTGTTAAGGAAGTTTGAGAGAGTTGTTGGATCATTTCACAAAGAACTATCAGAGAAGAAGCATGAAAATGAGCGACTTCAACGTATTTATGCAAGTGAACGTGAAATGTACAATCGTAGAATGGAAGAGATGGAAAGCGAAGTTGACCAACAACTGGAGCTCACAGAAATGAAAGCCCGGCAAGAGGAGCGAGACCGTCTGACAAAAGAGAAAGAAGAAATGCGCCAAAGAATGTCGGATGAAATGTCAGAAATGAGAAACAACATAGAAAGACTGCAGAAAATGGAAAAAGCTCTTGAACGGGAAAATGAACGGCTGAACCATCAAAAAGAGTTGTCTGATAAACTGAAAGTTGTAAATGAGGAAAATAACGACTTGCGACAAAATCTTGCTGAAAACCATCTCGAACTGGCGATGATCAAGAGTGAGCTTGCACAAGTCAGATGTGAATTTGATCAGAAGCAGGATGAACTTTCTGCAAGAAGAGATCAAGCTTCCCACGCCACCGAGGAAAGTGAATCTGTCAGGAAACAATTGCAACTCCTATTTGATGCCAATCGCAAATTGCACGAAACCAATGAAAGCCTCCGCGATGCACTGGATAGCAGAGCATCTGTTCTCCGACAGTTCAATTTGAGAACTCCTTCTCCCGGATTGCTCAGCTCGAATCGTAATTCTGTGGAGAACTTTCAAACGTCCACGAACGTGTTCCGATCCGTGCCACTTCATGCGATTAGTACTGAGGAGCAAGTGCCAGAAACATCATTGATTCTAGACGATGCGCATAGCTTGCAAGGGCTCGATACTCCGGGAGATCTTATGGGCCTCAATGATGCTAACGGCCCAGCTGAAAGAACGTTCCGTATCGTGATGTGTGGAGACGCCGCTGTTGGAAAAAACAGTTTTGTAATGCGGGTGATCCGTCGTCAATTCACAAATCAGCTCCCCAGCACACTTGGCGTAGATTTTCACGTGAAGACTGTCAATGTTGACGGAAGAAACGTTGCACTTCAGCTATGGGACACCGCGGGACAAGAACGTTTCCGATCACTTTGTAAATCATATTTTCGTCGAGCTGATGGAGCAATCCTAGTCTATGACGTCTGTGCGGAGCAGTCGTTTCTCCGTGTTCGCGACTGGATTGAGACTATAAAGGAGTCAACAGAAAGGAGTATTCCGATCATTTTGGTAGGAAACAAGGTTGATATGCGTATTTCGACGCCAGGCTCGGTGGCAAAGACAGATGGTGCAAGTATGGCTGCGGCGATGGGAGTTCTATTCATGGAAACCAGTGCTTTAGACGGAAGTAACATAGATAACGCTATGTTGGCACTAACCAGAGAGCTCATGGCAGTAGAAGATGTTGAAATTCGCTCAACAGGTGTTGTGCTCAATCCAGCAGTTGCAAAGAAGGGTGGATGTTTCTCAAAGTGTCGTGGTTCTTACCCAGCTTTCTTGTACAAAGTTGGCATTATAAGAAAGCATTGCTTATCAATTTGTTGCAACGAACAGGTCACTATCAGTCAAAATAAAATCATTATTTGCCATCCAGCTGCAGCTCTGGCCCGTGTCTCAAAATCTCTGATGTTACATTGCACAAGATAAAAATATATCATC

>FlOCS_AMT92-13_CeRabY1(CA)

TTGTCGAAACCCGAGGTGGAGAATCTCTTTTCTCTCTGCGACTCCGAATCCAAGGGATACCTGACGATGGAGGATTTGAGGAAAGTGTGCCCACAGCTTGATGATAATGATTTGCGTTTCATATTCACGGAGTTGGATCAAGATGGTTCTGGAAAAATTGAGAAGTTGGAATTTTTGCGGGGTTTCCAAGACACTGTACAGCACGGAGAAAGTCATGGCCTGAATGGTATGCAGCGTCGAGCATCGGTTGCTGTAGAGGACCCACCAATGTTTCTACGTGATGAACAAATGTTTGACAGTGAAAGTGACTCGACTTCCTCTCGACCAGCGATCCGAGTATTTGACGAAGAACACTATCATTCAGAGTCCGATACAAATATTAACATTGACTTTAGCGTTCCATGCCAAGAAGAAGTATTGGTGCTGTACGAACAACTACAGTCATCTGGTGTTCCTGCATTGTTAAGGAAGTTTGAGAGAGTTGTTGGATCATTTCACAAAGAACTATCAGAGAAGAAGCATGAAAATGAGCGACTTCAACGTATTTATGCAAGTGAACGTGAAATGTACAATCGTAGAATGGAAGAGATGGAAAGCGAAGTTGACCAACAACTGGAGCTCACAGAAATGAAAGCCCGGCAAGAGGAGCGAGACCGTCTGACAAAAGAGAAAGAAGAAATGCGCCAAAGAATGTCGGATGAAATGTCAGAAATGAGAAACAACATAGAAAGACTGCAGAAAATGGAAAAAGCTCTTGAACGGGAAAATGAACGGCTGAACCATCAAAAAGAGTTGTCTGATAAACTGAAAGTTGTAAATGAGGAAAATAACGACTTGCGACAAAATCTTGCTGAAAACCATCTCGAACTGGCGATGATCAAGAGTGAGCTTGCACAAGTCAGATGTGAATTTGATCAGAAGCAGGATGAACTTTCTGCAAGAAGAGATCAAGCTTCCCACGCCACCGAGGAAAGTGAATCTGTCAGGAAACAATTGCAACTCCTATTTGATGCCAATCGCAAATTGCACGAAACCAATGAAAGCCTCCGCGATGCACTGGATAGCAGAGCATCTGTTCTCCGACAGTTCAATTTGAGAACTCCTTCTCCCGGATTGCTCAGCTCGAATCGTAATTCTGTGGAGAACTTTCAAACGTCCACGAACGTGTTCCGATCCGTGCCACTTCATGCGATTAGTACTGAGGAGCAAGTGCCAGAAACATCATTGATTCTAGACGATGCGCATAGCTTGCAAGGGCTCGATACTCCGGGAGATCTTATGGGCCTCAATGATGCTAACGGCCCAGCTGAAAGAACGTTCCGTATCGTGATGTGTGGAGACGCCGCTGTTGGAAAAAGTAGTTTTGTAATGCGGGTGATCCGTCGTCAATTCACAAATCAGCTCCCCAGCACACTTGGCGTAGATTTTCACGTGAAGACTGTCAATGTTGACGGAAGAAACGTTGCACTTCAGCTATGGGACACCGCGGGCTTAGAACGTTTCCGATCACTTTGTAAATCATATTTTCGTCGAGCTGATGGAGCAATCCTAGTCTATGACGTCTGTGCGGAGCAGTCGTTTCTCCGTGTTCGCGACTGGATTGAGACTATAAAGGAGTCAACAGAAAGGAGTATTCCGATCATTTTGGTAGGAAACAAGGTTGATATGCGTATTTCGACGCCAGGCTCGGTGGCAAAGACAGATGGTGCAAGTATGGCTGCGGCGATGGGAGTTCTATTCATGGAAACCAGTGCTTTAGACGGAAGTAACATAGATAACGCTATGTTGGCACTAACCAGAGAGCTCATGGCAGTAGAAGATGTTGAAATTCGCTCAACAGGTGTTGTGCTCAATCCAGCAGTTGCAAAGAAGGGTGGATGTTTCTCAAAGTGTCGTGGTTCTT

>FlOCS_LGK3-2_RabY2(WT)

TGATTTTATTTTGACTGATAGTGACCTGTTCGTTGCAACAAATTGATGAGCAATGCTTTTTTATAATGCCAACTTTGTACAAAAAAGCAGGCTTGGAAGTAGAGTCGGCGACGAATTCGTCATCCGAGGAGTCTATTTTCCATGAACAAACACGAAAATTATTCCGCCTATGTGACACTAAGCACATTGGACTAATTGGGCAGTCAGATCTTGAAACACTCGTTGACCTAATCCCGCAAGACGATCTTCACAAAATTGCACGATTTATTGGTGACCAGAAAGTGAACGAGCAAGCATTTTGTCGCATTCTGAAAGCTATCGTCAATCAGTCACTCCAACAAAATATGGCCAAAAATGAAGTTGAGATTCCTTGCATTCTTGATAAAAGTCAATATTTGGAAGAGTCGAGTTTGGAGGATGAAATGCGGGAAATTGAACAAAACAAATCATACCTGGATGATCCATTGACCAAAATTTTGAAGAAAGAGTTAGAAGAGATTAAAAACTATGAAGATTTTCAAGTTAGAAATGAACAAGTACTTGATAACATAATCATCAAAAAACCGCTGTATCGTCCAATTCAACCAGAGCAGAGTATTCCAAAAGTATCGTCATTAGCTGAAGAACTGAATGCAATTGGAAAAAAAGTTCTACAAAAAGAAGAAATTGAAGAGGAAGTTACACAACCGGATCGAATCTTCAAAGTCGTTTTTGTTGGTGATTCGGCTGTTGGAAAAACTTGTTTTCTACATAGATTTTGTCACAACCGTTTCAAGCCTTTATTCAACGCTACCATTGGTGTTGATTTCACTGTGAAAACTATGAAAATTCCACCAAATAGAGCAATTGCAATGCAGTTATGGGATACAGCAGGGCAAGAAAGATTCCGCTCAATCACAAAACAATATTTCCGGAAGGCAGATGGAGTTGTGCTAATGTTTGATGTAACGTCAGAACAGAGTTTTCTAAATGTTAGAAATTGGATAGATTCCGTGAGAGCTGGAGTTGACGATGCAACTGTAATGTGCCTGGTCGGAAATAAAATGGATCTTTTTGGAAGTGACATAGCTAGGAGTGCCGTTTATCGAGCTGCTGAAAAATTGGCTGTGGAATTTAAAATACCGTTTTTCGAAACAAGTGCGTACACTGGGTTTGGAATTGACACTTGCATGCGTCAGATGGCAGAAAACCTTCAGCGGCGCGAAGACAACCATTTAGAAGAAGCGTTGAAGTTGGACATTAATTCGAACTATAAAAAAAGGAGCTGGTGCTGTATTTACCCAGCTTTCTTGTACAAAGTTGGCATTATAAGAAAGCATTGCTTATCAATTTGTTGCAACGAACAGGTCACTATCAGTCAAAATAAAATCATTATTTGCCATCCAGCTGCAGCTCTGGCCCGTGTCTCAAAATCTCTGATGTTACATTGCACAAGATAAAAATATATCATC

>FlOCS_LGK29-1_CeRabY2(DN)

ATAATGATTTTATTTTGACTGATAGTGACCTGTTCGTTGCAACAAATTGATGAGCAATGCTTTTTTATAATGCCAACTTTGTACAAAAAAGCAGGCTTGGAAGTAGAGTCGGCGACGAATTCGTCATCCGAGGAGTCTATTTTCCATGAACAAACACGAAAATTATTCCGCCTATGTGACACTAAGCACATTGGACTAATTGGGCAGTCAGATCTTGAAACACTCGTTGACCTAATCCCGCAAGACGATCTTCACAAAATTGCACGATTTATTGGTGACCAGAAAGTGAACGAGCAAGCATTTTGTCGCATTCTGAAAGCTATCGTCAATCAGTCACTCCAACAAAATATGGCCAAAAATGAAGTTGAGATTCCTTGCATTCTTGATAAAAGTCAATATTTGGAAGAGTCGAGTTTGGAGGATGAAATGCGGGAAATTGAACAAAACAAATCATACCTGGATGATCCATTGACCAAAATTTTGAAGAAAGAGTTAGAAGAGATTAAAAACTATGAAGATTTTCAAGTTAGAAATGAACAAGTACTTGATAACATAATCATCAAAAAACCGCTGTATCGTCCAATTCAACCAGAGCAGAGTATTCCAAAAGTATCGTCATTAGCTGAAGAACTGAATGCAATTGGAAAAAAAGTTCTACAAAAAGAAGAAATTGAAGAGGAAGTTACACAACCGGATCGAATCTTCAAAGTCGTTTTTGTTGGTGATTCGGCTGTTGGAAAAAATTGTTTTCTACATAGATTTTGTCACAACCGTTTCAAGCCTTTATTCAACGCTACCATTGGTGTTGATTTCACTGTGAAAACTATGAAAATTCCACCAAATAGAGCAATTGCAATGCAGTTATGGGATACAGCAGGGCAAGAAAGATTCCGCTCAATCACAAAACAATATTTCCGGAAGGCAGATGGAGTTGTGCTAATGTTTGATGTAACGTCAGAACAGAGTTTTCTAAATGTTAGAAATTGGATAGATTCCGTGAGAGCTGGAGTTGACGATGCAACTGTAATGTGCCTGGTCGGAAATAAAATGGATCTTTTTGGAAGTGACATAGCTAGGAGTGCCGTTTATCGAGCTGCTGAAAAATTGGCTGTGGAATTTAAAATACCGTTTTTCGAAACAAGTGCGTACACTGGGTTTGGAATTGACACTTGCATGCGTCAGATGGCAGAAAACCTTCAGCGGCGCGAAGACAACCATTTAGAAGAAGCGTTGAAGTTGGACATTAATTCGAACTATAAAAAAAGGAGCTGGTGCTGTATTTACCCAGCTTTCTTGTACAAAGTTGGCATTATAAGAAAGCATTGCTTATCAATTTGTTGCAACGAACAGGTCACTATCAGTCAAAATAAAATCATTATTTGCCATCCAGCTGCAGCTCTGGCCCGTGTCTCAAAATCTCTGATGTTACATTGCACAAGATAAAAATATATCATCA

>FlOCS_AMT92-5_CeRabY2(CA)

GATTTTATTTTGACTGATAGTGACCTGTTCGTTGCAACAAATTGATGAGCAATGCTTTTTTATAATGCCAACTTTGTACAAAAAAGCAGGCTTGGAAGTAGAGTCGGCGACGAATTCGTCATCCGAGGAGTCTATTTTCCATGAACAAACACGAAAATTATTCCGCCTATGTGACACTAAGCACATTGGACTAATTGGGCAGTCAGATCTTGAAACACTCGTTGACCTAATCCCGCAAGACGATCTTCACAAAATTGCACGATTTATTGGTGACCAGAAAGTGAACGAGCAAGCATTTTGTCGCATTCTGAAAGCTATCGTCAATCAGTCACTCCAACAAAATATGGCCAAAAATGAAGTTGAGATTCCTTGCATTCTTGATAAAAGTCAATATTTGGAAGAGTCGAGTTTGGAGGATGAAATGCGGGAAATTGAACAAAACAAATCATACCTGGATGATCCATTGACCAAAATTTTGAAGAAAGAGTTAGAAGAGATTAAAAACTATGAAGATTTTCAAGTTAGAAATGAACAAGTACTTGATAACATAATCATCAAAAAACCGCTGTATCGTCCAATTCAACCAGAGCAGAGTATTCCAAAAGTATCGTCATTAGCTGAAGAACTGAATGCAATTGGAAAAAAAGTTCTACAAAAAGAAGAAATTGAAGAGGAAGTTACACAACCGGATCGAATCTTCAAAGTCGTTTTTGTTGGTGATTCGGCTGTTGGAAAAACTTGTTTTCTACATAGATTTTGTCACAACCGTTTCAAGCCTTTATTCAACGCTACCATTGGTGTTGATTTCACTGTGAAAACTATGAAAATTCCACCAAATAGAGCAATTGCAATGCAGTTATGGGATACAGCAGGGCTCGAAAGATTCCGCTCAATCACAAAACAATATTTCCGGAAGGCAGATGGAGTTGTGCTAATGTTTGATGTAACGTCAGAACAGAGTTTTCTAAATGTTAGAAATTGGATAGATTCCGTGAGAGCTGGAGTTGACGATGCAACTGTAATGTGCCTGGTCGGAAATAAAATGGATCTTTTTGGAAGTGACATAGCTAGGAGTGCCGTTTATCGAGCTGCTGAAAAATTGGCTGTGGAATTTAAAATACCGTTTTTCGAAACAAGTGCGTACACTGGGTTTGGAATTGACACTTGCATGCGTCAGATGGCAGAAAACCTTCAGCGGCGCGAAGACAACCATTTAGAAGAAGCGTTGAAGTTGGACATTAATTCGAACTATAAAAAAAGGAGCTGGTGCTGTATTTACCCAGCTTTCTTGTACAAAGTTGGCATTATAAGAAAGCATTGCTTATCAATTTGTTGCAACGAACAGGTCACTATCAGTCAAAATAAAATCATTATTTGCCATCCAGCTGCAGCTCTGGCCCGTGTCTCAAAATCTCTGATGTTACATTGCACAAGATAAAAATATATCATCA

>FlOCS_KK9-2_CeRabY4(WT)

AATAATGATTTTATTTTGACTGATAGTGACCTGTTCGTTGCAACAAATTGATGAGCAATGCTTTTTTATAATGCCAACTTTGTACAAAAAAGCAGGCTTGTCATCAGATCATGTGTTCAAATATGTCATAATCGGAGACCGTGGCGTCGGAAAGTCTAACTTGCTGCTGCGATTCATTGGCAAACCTTTCGATTCAATCCATCCGTCGACACTTGGAATTGAGTTTGGTTTTCGAAATCTGGAAATTGACAGGAAAAAGGTGAAGCTTCATGTCTGGGATACGTGCGGCCAGGAGAGATTTCGGTCACTTGTTGGATCATATTATCGGCATGCAATTGGTGCACTTCTTGTCTATGATATTACTAGTCGTGAGTCCTTCTACCATTTGGAGCACTGGCTCACAGATCTCCAACGACTGGGTGATCCCGATATAGTTATAGTGTTGATTGGCAACAAAAGTGATCTAGAAGCAGATCGTGAAGTCAGAAAAGAAGAAGGTGAAGCATTTGCAAGAGAGTTTGGACTTATATTTATGGAAATTTCTGCAAAAACCAATGAATATGTGGAGGAAGCATTTGTCAATTCAGCTCACGAGATTTATAGAAAGCTTAACTTTGGAATTATCAAAGAGATATATGTGAAGAAGAAGAAGAAGAAAATGAATATAATTATACGATCTATATCGGGAAAGGAGAAAGCATGTTGTTACCCAGCTTTCTTGTACAAAGTTGGCATTATAAGAAAGCATTGCTTATCAATTTGTTGCAACGAACAGGTCACTATCAGTCAAAATAAAATCATTATTTGCCATCCAGCTGCAGCTCTGGCCCGTGTCTCAAAATCTCTGATGTTACATTGCACAAGATAAAAATATATCATCATGAACAATAAAACTGTCTGCTTACATAAACAGTAATAC

>FlOCS_AMT92-1_CeRabY4(DN)

GATTTTATTTTGACTGATAGTGACCTGTTCGTTGCAACAAATTGATGAGCAATGCTTTTTTATAATGCCAACTTTGTACAAAAAAGCAGGCTTGTCATCAGATCATGTGTTCAAATATGTCATAATCGGAGACCGTGGCGTCGGAAAGAACAACTTGCTGCTGCGATTCATTGGCAAACCTTTCGATTCAATCCATCCGTCGACACTTGGAATTGAGTTTGGTTTTCGAAATCTGGAAATTGACAGGAAAAAGGTGAAGCTTCATGTCTGGGATACGTGCGGCCAGGAGAGATTTCGGTCACTTGTTGGATCATATTATCGGCATGCAATTGGTGCACTTCTTGTCTATGATATTACTAGTCGTGAGTCCTTCTACCATTTGGAGCACTGGCTCACAGATCTCCAACGACTGGGTGATCCCGATATAGTTATAGTGTTGATTGGCAACAAAAGTGATCTAGAAGCAGATCGTGAAGTCAGAAAAGAAGAAGGTGAAGCATTTGCAAGAGAGTTTGGACTTATATTTATGGAAATTTCTGCAAAAACCAATGAATATGTGGAGGAAGCATTTGTCAATTCAGCTCACGAGATTTATAGAAAGCTTAACTTTGGAATTATCAAAGAGATATATGTGAAGAAGAAGAAGAAGAAAATGAATATAATTATACGATCTATATCGGGAAAGGAGAAAGCATGTTGTTACCCAGCTTTCTTGTACAAAGTTGGCATTATAAGAAAGCATTGCTTATCAATTTGTTGCAACGAACAGGTCACTATCAGTCAAAATAAAATCATTATTTGCCATCCAGCTGCAGCTCTGGCCCGTGTCTCAAAATCTCTGATGTTACATTGCACAAGATAAAAATATATCATCATGAACAATAAAACTGTCTGCTTACATAAACAGTAATACAAGGGGTGTTATGAGCCATATTCAACGGGAAACGTCGAGGCCGCGATTAAATTCCAACATGG

>FlOCS_AMT92-9_CeRabY4(CA)

TGATGATATATTTTTATCTTGTGCAATGTAACATCAGAGATTTTGAGACACGGGCCAGAGCTGCAGCTGGATGGCAAATAATGATTTTATTTTGACTGATAGTGACCTGTTCGTTGCAACAAATTGATAAGCAATGCTTTCTTATAATGCCAACTTTGTACAAGAAAGCTGGGTAACAACATGCTTTCTCCTTTCCCGATATAGATCGTATAATTATATTCATTTTCTTCTTCTTCTTCTTCACATATATCTCTTTGATAATTCCAAAGTTAAGCTTTCTATAAATCTCGTGAGCTGAATTGACAAATGCTTCCTCCACATATTCATTGGTTTTTGCAGAAATTTCCATAAATATAAGTCCAAACTCTCTTGCAAATGCTTCACCTTCTTCTTTTCTGACTTCACGATCTGCTTCTAGATCACTTTTGTTGCCAATCAACACTATAACTATATCGGGATCACCCAGTCGTTGGAGATCTGTGAGCCAGTGCTCCAAATGGTAGAAGGACTCACGACTAGTAATATCATAGACAAGAAGTGCACCAATTGCATGCCGATAATATGATCCAACAAGTGACCGAAATCTCTCGAGTCCGCACGTATCCCAGACATGAAGCTTCACCTTTTTCCTGTCAATTTCCAGATTTCGAAAACCAAACTCAATTCCAAGTGTCGACGGATGGATTGAATCGAAAGGTTTGCCAATGAATCGCAGCAGCAAGTTAGACTTTCCGACGCCACGGTCTCCGATTATGACATATTTGAACACATGATCTGATGACAAGCCTGCTTTTTTGTACAAAGTTGGCATTATAAAAAAGCATTGCTCATCAATTTGTTGCAACGAACAGGTCACTATCAGTCAAAATAAAATCATTATTTGGGGCCCGAGATCCATGCTAGCGTTAACGCGAGAGTAGGGAACTGCCAGGCATCAAATAAAACGAAA

>FlOCS_EB8-2_rab-2-like(WT)

GCGATATTGAGACTAATATGATTTTATTTTGACTGATAGTGACCTGTTCGTTGCAACAAATTGATGAGCAATGCTTTTTTATAATGCCAACTTTGTACAAAAAAGCAGGCTTGTACCCTGATCACATGTTCAAATATGTGATAATCGGAGACGGAGGCGTCGGAAAGTCTAATCTATTGCTGCGATTCACCGACGAACTTTTTGATCCGATTCATACAACGACGCTTGGAGTTGAATTCGGGTATAAGGATCTGCAGATTGACAAATACAAAGTGAGGCTTCGTGTTTGGGATACGTGCGGACAAGAGAACTTTCGGTCAATTATTCGTGCCTACTATCGGAATGCACTTGGAGCACTTCTTGTTTATGATATAACTTGTCGTAAGTCCTTCGTTCACTTGGAGCAGTGGCTCTCTGATCTTCGACAACATGGGCATCCCGAGATGGTGATAATGCTGATTGGCAACAAAAGTGATCTTAAAGCGGTCCGTGACGTCACAACGGAAGAAGGTGAAGCATTTGCTAAAAAGAATGGCCTGACGTTTATGGAAACTTCTGCAAAAGCCAATAAGCATGTGGAGAAGGCATTCGTGAATACAGCTCATGAGATTTATAAAAAGCTTAAGCTTGGAGTGATTGAAGACGACGACGTGAAAAAGAAAAAAATTGGTATAATTTTACGCTCTAGATCCGGAAAAGAGAAAAAATGTTGTTACCCAGCTTTCTTGTACAAAGTTGGCATTATAAGAAAGCATTGCTTATCAATTTGTTGCAACGAACAGGTCACTATCAGTCAAAATAAAATCATTATTTGCCATCCAGCTGCAGCTCTGGGCCGTGTCTCAAATCTCTGATGTTACATTTGCACAGATGCGTATTATTCATCATGACAATAAACCTGTCTGCTACATAAAGGTAATACCAGGGGGGG

>FlOCS_MR49-1_rab-2-like(DN)

ATAATGATTTTATTTTGACTGATAGTGACCTGTTCGTTGCAACAAATTGATGAGCAATGCTTTTTTATAATGCCAACTTTGTACAAAAAAGCAGGCTTGTACCCTGATCACATGTTCAAATATGTGATAATCGGAGACGGAGGCGTCGGAAAGAACAATCTATTGCTGCGATTCACCGACGAACTTTTTGATCCGATTCATACAACGACGCTTGGAGTTGAATTCGGGTATAAGGATCTGCAGATTGACAAATACAAAGTGAGGCTTCGTGTTTGGGATACGTGCGGACAAGAGAACTTTCGGTCAATTATTCGTGCCTACTATCGGAATGCACTTGGAGCACTTCTTGTTTATGATATAACTTGTCGTAAGTCCTTCGTTCACTTGGAGCAGTGGCTCTCTGATCTTCGACAACATGGGCATCCCGAGATGGTGATAATGCTGATTGGCAACAAAAGTGATCTTAAAGCGGTCCGTGACGTCACAACGGAAGAAGGTGAAGCATTTGCTAAAAAGAATGGCCTGACGTTTATGGAAACTTCTGCAAAAGCCAATAAGCATGTGGAGAAGGCATTCGTGAATACAGCTCATGAGATTTATAAAAAGCTTAAGCTTGGAGTGATTGAAGACGACGATGTGAAAAAGAAAAAAATTGGTATAATTTTACGCTCTAGATCCGGAAAAGAGAAAAAATGTTGTTACCCAGCTTTCTTGTACAAAGTTGGCATTATAAGAAAGCATTGCTTATCAATTTGTTGCAACGAACAGGTCACTATCAGTCAAAATAAAATCATTATTTGCCATCCAGCTGCAGCTCTGGCCCGTGTCTCAAAATCTCTGATGTTACATTGC

>FlOCS_PM16-1_rab-2-like(CA)

ATGATTTTATTTTGACTGATAGTGACCTGTTCGTTGCAACAAATTGATGAGCAATGCTTTTTTATAATGCCAACTTTGTACAAAAAAGCAGGCTTGTACCCTGATCACATGTTCAAATATGTGATAATCGGAGACGGAGGCGTCGGAAAGTCTAATCTATTGCTGCGATTCACCGACGAACTTTTTGATCCGATTCATACAACGACGCTTGGAGTTGAATTCGGGTATAAGGATCTGCAGATTGACAAATACAAAGTGAGGCTTCGTGTTTGGGATACGTGCGGACTCGAGAACTTTCGGTCAATTATTCGTGCCTACTATCGGAATGCACTTGGAGCACTTCTTGTTTATGATATAACTTGTCGTAAGTCCTTCGTTCACTTGGAGCAGTGGCTCTCTGATCTTCGACAACATGGGCATCCCGAGATGGTGATAATGCTGATTGGCAACAAAAGTGATCTTAAAGCGGTCCGTGACGTCACAACGGAAGAAGGTGAAGCATTTGCTAAAAAGAATGGCCTGACGTTTATGGAAACTTCTGCAAAAGCCAATAAGCATGTGGAGAAGGCATTCGTGAATACAGCTCATGAGATTTATAAAAAGCTTAAGCTTGGAGTGATTGAAGACGACGATGTGAAAAAGAAAAAAATTGGTATAATTTTACGCTCTAGATCCGGAAAAGAGAAAAAATGTTGTTACCCAGCTTTCTTGTACAAAGTTGGCATTATAAGAAAGCATTGCTTATCAATTTGTTGCAACGAACAGGTCACTATCAGTCAAAATAAAATCATTATTTGCCATCCAGCTGCAGCTCTGGCCCGTGTCTCAAAATCTCTGATGTTACATTGCACAAGATAAAAATATATCATCATGAACAATAAAACTGTCTGCTTA
