# Supplementary material for: The C. elegans Rab Family: Identification, Classification and Toolkit Construction
Source: PLoS One. 2012 Nov 21;7(11):e49387. doi: 10.1371/journal.pone.0049387 (PMC3504004; doi:10.1371/journal.pone.0049387)
Supplement: Table S1 — A list of mutagenic primers (forward only) used in site-directed mutagenesis of mutant Rab forms. The mismatch region is highlighted in bold and all caps. For a definition of mismatch and the equation used to calculate Tm, see methods. Where applicable, the diagnostic enzyme and the form it digests is indicated. m = mutant, wt = wild type. (DOC) [file pone.0049387.s006.doc]

**Supplementary Table 1.**

| Gene Name | Form | Forward Mutagenic Primer Sequence | Diagnostic Enzyme | Len (bp) | Tm (°C) |
| --- | --- | --- | --- | --- | --- |
| *rab-1* | DN | gattctggcgtcggaaaa**AAC**tgcctgttgctgcgtttc | ***none*** | 39 | 84.4 |
| *rab-1* | CA | ctgggacactgctggtc**TC**gaacgtttccgcacaattac | BsmAI (m) | 39 | 85.4 |
| *unc-108/rab-2* | DN | ggatactggagtaggaaaa**AAC**tgcttgctccttcagtttac | ***none*** | 42 | 82.1 |
| *unc-108/rab-2* | CA | caaatttgggacacagccgg**CCT**agaatcattccgctccatc | HaeIII (m) | 42 | 85.2 |
| *rab-3* | DN | ggaaattcatcagttggaaa**GAAT**tcattcctcttccg | EcoRI (m) | 38 | 78.5 |
| *rab-3* | CA | ggataccgccggac**TC**gagaggtacc | HinfI (m) | 26 | 80.7 |
| *rab-5* | DN | gctgtaggcaa**GAAT**tctctcgtattgcgattcg | EcoRI (m) | 34 | 79.5 |
| *rab-5* | CA | gggatactgcaggac**TC**gaaagatatcattcattggc | HinfI (m) | 37 | 81.0 |
| *rab-6.1* | DN | gcgaacagagtgtgggaaa**GAA**ttctatcatcaccagattc | EcoRI (m) | 41 | 82.1 |
| *rab-6.1* | CA | caattgtgggataccgctggc**TTA**gaacgtttccgatc | HaeIII (wt) | 38 | 83.3 |
| *rab-6.2* | DN | ggaacaaagtgtcggaaaaa**A**ctccctaatcactcgc | MnlI (wt) | 37 | 81.0 |
| *rab-6.2* | CA | gggacaccgctggac**T**ggaacgtttccgctc | BsrI (m) | 31 | 86.3 |
| *rab-7* | DN | cgggcgttggaaa**GAAT**tctttgatgaatcaatatg | EcoRI (m) | 36 | 77.1 |
| *rab-7* | CA | gatacagccggc**TTG**gaacgtttccaatc | HaeIII (wt) | 29 | 77.6 |
| *rab-8* | DN | gtggagtcggcaaga**AT**tgcgtactgttcc | HpyCH4IV (wt) | 30 | 80.8 |
| *rab-8* | CA | gggacacagccgg**CCT**ggagagattcagaactatc | HaeIII (m) | 35 | 83.5 |
| *rab-10* | DN | cgactcaggagttggaaaaa**A**ttgcattctgtacag | Tsp509I (m) | 36 | 79.8 |
| *rab-10* | CA | gggacacagctggac**TC**gaacgattccacac | HinfI (m) | 31 | 82.3 |
| *rab-11.1* | DN | gagactcaggcgtcggaaag**AAT**aatctcctgtctcgtttcac | HinfI (wt) | 43 | 86.2 |
| *rab-11.1* | CA | tgggatactgctggac**T**ggaacgttaccgtgcc | BsrI (m) | 33 | 84.8 |
| *rab-14* | DN | gatatgggtgtcggaaaa**AAC**tgtcttcttcatcagttcac | HpyCH4III (m) | 41 | 82.1 |
| *rab-14* | CA | ggacacggcgggcc**TC**gagcgattccgc | XhoI (m) | 28 | 87.1 |
| *rab-19* | DN | cgatatgggagtcggaaaga**AC**tgtgtagttcaacgc | AflIII (wt) | 37 | 82.1 |
| *rab-19* | CA | ctgggatactggaggcc**TT**gaacgattccgaacg | BstNI (wt) | 34 | 84.8 |
| *rab-21* | DN | cgaaggatgtgttggtaa**GAAT**tcacttgtgcttcg | EcoRI (m) | 36 | 79.6 |
| *rab-21* | CA | ggataccgctggac**TC**gagaaataccacgc | Xho I (m) | 30 | 82.3 |
| *aex-6/rab-27* | DN | gattcgggagtcggaaaaa**AC**tcgtttttgcatcgttacac | HpyCH4IV (wt) | 41 | 82.1 |
| *aex-6/rab-27* | CA | gggacactgccgg**TCT**agagagattccgttctctaac | BfaI (m) | 37 | 84.6 |
| *rab-28* | DN | gatggagcaagtggaaaga**AT**tcaatttgccagag | EcoRI (m) | 35 | 79.7 |
| *rab-28* | CA | ggtatgggatattggaggac**T**aagtattgctggtgaaatg | DdeI (m) | 40 | 82.1 |
| *rab-30* | DN | gcgggcgttgggaaaa**AC**tgtctagtcagaaag | AflIII (wt) | 33 | 80.9 |
| *rab-30* | CA | gggatacggcaggtc**TC**gagaggtttcgatcg | TaqI (m) | 32 | 83.6 |
| *rab-33* | DN | cggccgtcggaaaaa**AC**tgtctttcattccg | HpyCH4IV (wt) | 31 | 79.4 |
| *rab-33* | CA | ctatgggatactgctggac**T**ggaaagatatcgacaatcg | BsrI (m) | 39 | 83.2 |
| *rab-37* | DN | cagctgcactggaaaaa**AC**tgccttctgattcg | HpyCH4IV (wt) | 33 | 80.9 |
| *rab-37* | CA | gacaccgcgggac**TC**gaacgttttcgatc | TaqI (m) | 29 | 80.8 |
| *tag-312/CeRAbY1* | DN | gccgctgttggaaaaa**AC**agttttgtaatgcggg | HpyCH4III (m) | 34 | 79.6 |
| *tag-312/CeRAbY1* | CA | ggacaccgcggg**CTT**agaacgtttcc | DdeI (m) | 26 | 78.9 |
| *4R79.2/CaRabY2* | DN | ggtgattcggctgttggaaaaa**A**ttgttttctacatag | Tsp509I (m) | 38 | 78.8 |
| *4R79.2/CaRabY2* | CA | ggatacagcaggg**CTC**gaaagattccgc | BanII (m) | 28 | 79.2 |
| *K02E10.1/CeRabY4* | DN | cgtggcgtcggaaag**AAC**aacttgctgctg | ***none*** | 30 | 80.8 |
| *K02E10.1/CeRabY4* | CA | gatacgtgcgg**ACTC**gagagatttcggtcac | XhoI (m) | 31 | 79.3 |
| *F11A5.3/CeRabY5* | DN | gaggcgtcggaaag**AAC**aatctattgctgcg | ***none*** | 31 | 79.4 |
| *F11A5.3/CeRabY5* | CA | gggatacgtgcggac**TC**gagaactttcgg | XhoI (m) | 29 | 80.8 |
|  |  |  |  |  |  |
|  |  |  | Minimum | 26 | 77.1 |
|  |  |  | Maximum | 43 | 87.1 |
